# Supplementary material for: Sequestration and suppressed synthesis of oncogenic HMGA1 using engineered adenoviruses decreases human pancreatic and breast cancer cell characteristics
Source: PLoS One. 2025 Nov 3;20(11):e0335934. doi: 10.1371/journal.pone.0335934 (PMC12582467; doi:10.1371/journal.pone.0335934)
Supplement: S1 File — This file contains the supplementary methods (S Method 1), supplementary figures (S Fig. 1–51) and supplementary tables (S Tables 1–17). (PDF) [file pone.0335934.s001.pdf]

Supplementary Information for

Sequestration and suppressed synthesis of oncogenic HMGA1 using engineered adenoviruses decreases human pancreatic and breast cancer cell characteristics

Md. Sharif Hasan [1], Shuisong Ni [1], Fatema B. Kamal [2,3], Megan F. Blossey[1], Eian Vargas[1], Margaret B. Bogomolny[1], Trang Dinh[1], Michael A. Kennedy [1]*

[1] Department of Chemistry and Biochemistry, Miami University, Oxford, OH 45056.

[2] Mailman School of Public Health, Columbia University, New York, NY 10032.

[3] ORISE/CDC, National Center for HIV, Viral Hepatitis, STD and TB Prevention.

Corresponding Author:

Michael A. Kennedy

Email: kennedm4@miamioh.edu

This file contains

Supplementary Methods (S Method 1)

Supplementary Methods

To determine the correct statistical tests, data assumptions were checked by performing a Shapiro-Wilk normality test and a Levene's homogeneity of variances test (1). From those tests, it was found that the data were not normally distributed (p-value < 0.05 in normality test), and the sample variances were not equal (p-value < 0.05 in Levene's test). Therefore, the Kruskal Wallis ANOVA followed by a correct post-hoc analysis was used for multiple comparisons. Data assumptions were assessed to ensure the accuracy of the model interpretation and prediction (2). To perform the regression analysis and evaluate the variable relationships, both linear models (UVM) and multilinear models (MVM) were used (with (+)/without (-) interactions) (3). The variance inflation factor (VIF) values were also evaluated to measure any hidden interactions (multicollinearity) among independent variables in multilinear (no interaction) models (4). VIF values greater than 10 indicate significant interactions between independent variables (4). As most of the models had very high VIF values, the beta coefficients were not evaluated because at high VIF, the coefficient values are inflated and not reliable to predict the model outcome (5). From the linear model data, most variables showed statistically significant (p <0.05) effects (**S Table 3-9**), however using a multilinear model, those significant effects disappeared (**S Table 10-17**). For example, in breast epithelial and ZR-75 breast cancer cells, both HMGA1 and NAT showed significant effects (p<0.05) on protein level individually while applying linear regression (**S Table 3** yellow box). However, this significance was lost (p>0.05) in multiple linear regression in which both HMGA1 and NAT effect was considered at the same time (**S Table 10** yellow box). This illustrates that p-values alone are not always robust and do not necessarily provide a meaningful indication of the real impact of the variable effect (6). As we are dealing with biological systems, it is reasonable to assume that the multilinear models provide more meaningful scenarios than linear models, and therefore only the multilinear model results are discussed for further analysis presented below. By using Akaike information criterion (AIC) values, the best multilinear model (lower AIC value) was used for further data interpretation (**S Table 10-17**) (7). The effect sizes were also calculated, which were measures of strength of the impact of each variable, whether the p-values were significant or not (8). In the case of small sample sizes, effect sizes can be more informative than the associated p-values (9). A biological experiment with higher effect size and a p > 0.05 can be more relevant than a small effect size with a p < 0.01 in another experiment (10-12). Recently, scientific communities are raising questions about their study conclusions based solely on p-value assessment and suggesting including effect size analysis along with p-values for the best interpretation of biological studies (13).

Choosing a correct formula for the effect size calculation can be challenging and depends on the type of research, interpretation of the effects and, finally, objective goals for the interpretation of the research results (14). As our goal was to understand how HMGA1 transcripts and NATs (with or without interactions) determined HMGA1 protein levels in both healthy and cancer cells (with/without viral infection) and to predict how the engineered viruses reduced HMGA1 protein levels, a partial eta squared formula was chosen to calculate the effects of HMGA1 and NAT transcripts (with/without interactions) in determining HMGA1 protein levels because the partial eta squared is less biased than eta squared and more intuitive in multilinear regression models, and most importantly, it allows comparison of the effect of same variable in different experiments or studies where other factors or conditions are present (15). This property was necessary in this case because the HMGA1 transcript and NAT levels had to be measured in different cell types and under different experimental treatments. After calculating the effect sizes, the data was interpreted using previously published guidelines (16) to interpret the mechanism of engineered viral vectors in reducing HMGA1 protein level in cancer cells.

Supplementary references

1. Ghasemi A, Zahediasl S. Normality Tests for Statistical Analysis: A Guide for Non-Statisticians. International Journal of Endocrinology and Metabolism/International Journal of Endocrinology and Metabolism. 2012;10(2):486–489. https://doi.org/10.5812/ijem.3505. doi:10.5812/ijem.3505
2. Shatz I. Assumption-checking rather than (just) testing: The importance of visualization and effect size in statistical diagnostics. Behavior Research Methods. 2023;56(2):826–845. https://doi.org/10.3758/s13428-023-02072-x. doi:10.3758/s13428-023-02072-x
3. Muller KE, Lavange LM, Ramey SL, Ramey CT. Power calculations for general linear multivariate models including repeated measures applications. Journal of the American Statistical Association. 1992;87(420):1209–1226. https://doi.org/10.1080/01621459.1992.10476281. doi:10.1080/01621459.1992.10476281
4. Kim JH. Multicollinearity and misleading statistical results. Korean Journal of Anesthesiology. 2019;72(6):558–569. https://doi.org/10.4097/kja.19087. doi:10.4097/kja.19087
5. Frost J. Regression analysis: An Intuitive Guide for Using and Interpreting Linear Models. Statistics By Jim Publishing; 2019.
6. Sullivan GM, Feinn R. Using effect size—or why the P value is not enough. Journal of Graduate Medical Education. 2012;4(3):279–282. https://doi.org/10.4300/jgme-d-12-00156.1. doi:10.4300/jgme-d-12-00156.1
7. McElreath R. Statistical rethinking: A Bayesian Course with Examples in R and Stan. CRC Press; 2018.

8. Dunkler D, Haller M, Oberbauer R, Heinze G. To test or to estimate? P -values versus effect sizes. Transplant International. 2019;33(1):50–55. https://doi.org/10.1111/tri.13535. doi:10.1111/tri.13535
9. Miola AC, Miot HA. P-value and effect-size in clinical and experimental studies. J. Vasc. Bras. 2021;20. https://doi.org/10.1590/1677-5449.210038. doi:10.1590/1677-5449.210038
10. Nuzzo R. Scientific method: Statistical errors. Nature. 2014;506(7487):150–152. https://doi.org/10.1038/506150a. doi:10.1038/506150a
11. Matthews JNS, Altman DG. Statistics Notes: Interaction 2: compare effect sizes not P values. BMJ. British Medical Journal. 1996;313(7060):808. https://doi.org/10.1136/bmj.313.7060.808. doi:10.1136/bmj.313.7060.808
12. Fleischmann M, Vaughan B. Commentary: Statistical significance and clinical significance - A call to consider patient reported outcome measures, effect size, confidence interval and minimal clinically important difference (MCID). Journal of Bodywork and Movement Therapies. 2019;23(4):690–694. https://doi.org/10.1016/j.jbmt.2019.02.009. doi:10.1016/j.jbmt.2019.02.009
13. Ioannidis JPA. What Have We (Not) Learnt from Millions of Scientific Papers with P Values? the American Statistician. 2019;73(sup1):20–25. https://doi.org/10.1080/00031305.2018.1447512. doi:10.1080/00031305.2018.1447512
14. Durlak JA. How to select, Calculate, and interpret effect sizes. Journal of Pediatric Psychology. 2009;34(9):917–928. https://doi.org/10.1093/jpepsy/jsp004. doi:10.1093/jpepsy/jsp004
15. Mordkoff JT. A simple method for removing bias from a popular measure of standardized effect size: adjusted partial ETA squared. Advances in Methods and Practices in Psychological Science. 2019;2(3):228–232. https://doi.org/10.1177/2515245919855053. doi:10.1177/2515245919855053
16. Bakker A, Cai J, English L, Kaiser G, Mesa V, Van Dooren W. Beyond small, medium, or large: points of consideration when interpreting effect sizes. Educational Studies in Mathematics. 2019;102(1):1–8. https://doi.org/10.1007/s10649-019-09908-4. doi:10.1007/s10649-019-09908-4

Supplementary Information for

Sequestration and suppressed synthesis of oncogenic HMGA1 using engineered adenoviruses decreases human pancreatic and breast cancer cell characteristics

Md. Sharif Hasan [1], Shuisong Ni [1], Fatema B. Kamal [2,3], Megan F. Blossey[1], Eian Vargas[1], Margaret B. Bogomolny[1], Trang Dinh[1], Michael A. Kennedy [1]*

[1] Department of Chemistry and Biochemistry, Miami University, Oxford, OH 45056.

[2] Mailman School of Public Health, Columbia University, New York, NY 10032.

[3] ORISE/CDC, National Center for HIV, Viral Hepatitis, STD and TB Prevention.

Corresponding Author:
Michael A. Kennedy
Email: kennedm4@miamioh.edu

This file contains
Supplementary figures (S Fig. 1-51)
Supplementary tables (Table 1-17)

1A

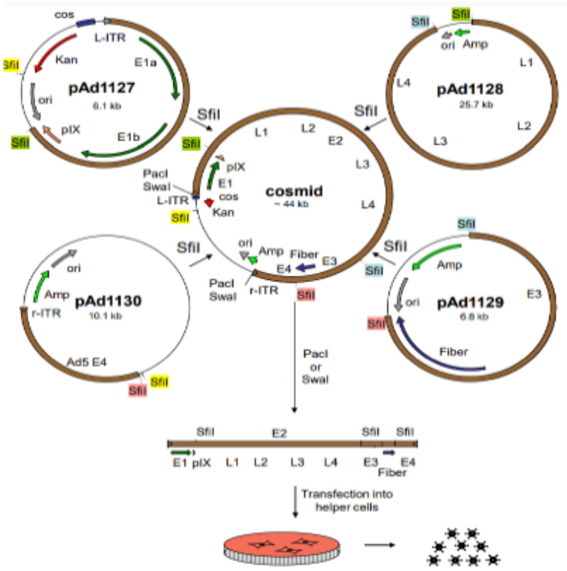

1B

HMGA1 antisense sequence
5'GAATTCTCACTGCTCCTCCTCCGAGGACTCCTGCGAGATGCCCTCCTCTTCCTCCTTCTCC
TTTTTGGGTCTGCCCCTTGGTTTCCTTCCTGGAGTTGTGGTGGTTTTCCGGGTCTTGGCAGC
CTTGTTTTTGCTTCCCTTTGGTCGGCCCCGAGGTCTCTTAGGTGTTGGCACTTCGCTGGGCT
TCTGACTCCCTACCAGCGCTGTCCCGGGACTCACCGGAGGCTGCTTGCGCGGCCTGCCCC
CCCGCTTCTCAGTGCCGTCCTTTTCCTGCTTGGAGGCCAAGGGCTGGCTGGACTTCGAGCT
CTCACTCATTCTAGA-3'

1C

HMGA1 shRNA sequence- 5'-CCGG-CAACTCCAGGAAGGAAACCAA-CTCGAG-TTGGTTTCCTTCCTGGAGTTG-TTTTT-3

Target sequence- CAACTCCAGGAAGGAAACCAA

RNAi consortium website https://portals.broadinstitute.org/gpp/public/gene/details?geneId=3159

S Fig.1: Schematic diagram adenoviral vector generation and the antisense and shRNA sequences of HMGA1 transcript. (A) Flow chart of how the engineered adenoviral vector was generated according to manufacturer's protocol. (B) Antisense sequence of HMGA1 transcript that was inserted into adenoviral vector under CMV promoter to generate AAT virus. (C) Short hairpin RNA sequence of HMGA1 transcript that was inserted into adenoviral vector under CMV promoter to generate shRNA virus. This sequence was taken from RNAi consortium website of Broad Institute.

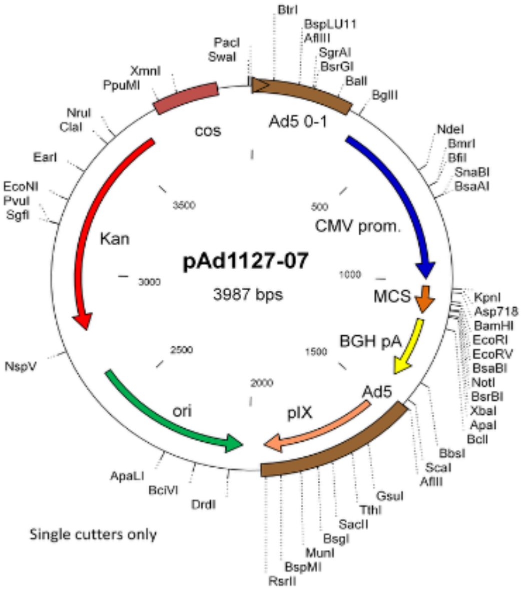

S Fig. 2: One of the four plasmids that contains CMV promoter and MCS site (pAd1127-07). Both antisense sequence and shRNA sequences were inserted separately in the MCS site. We chose EcoRI and XbaI as our restriction sites for inserting the antisense and shRNA sequences.

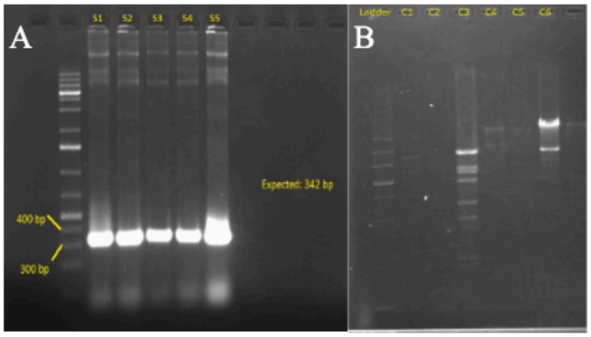

S Fig. 3: (A) PCR amplification of AAT insert sequence from pAd1127-07 plasmids extracted from E.coli. Expected band size of the product was 342 bp. Column S1 to S5 indicate different plasmid samples. (B) Characterization of cosmid plasmid digestion by HindIII restriction enzyme. C1 to C6 indicate different colony types. Only C3 shows corrected band pattern which was confirmed by the manufacturer.

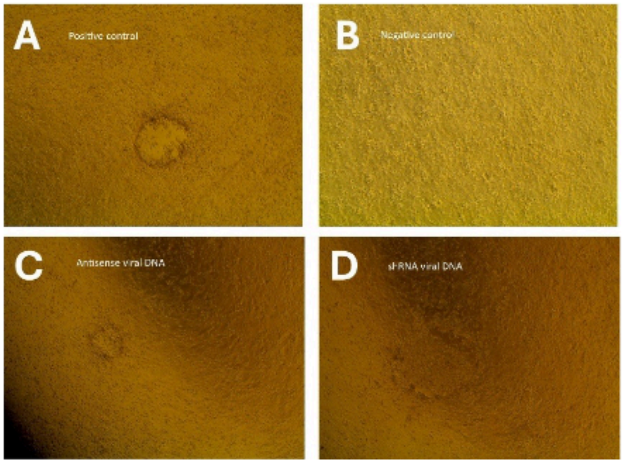

S Fig. 4: Plaque formation after linearized DNA transfection in the Ad293 cells. (A) Plaque formation from linearized DNA supplied by the manufacturer (positive control). (B) No plaque was generated by the addition of vehicle which contains no DNA (negative control). (C) Linearized AAT adenoviral DNA transfected plaque formation. (D) Linearized shRNA adenoviral DNA transfected plaque formation.

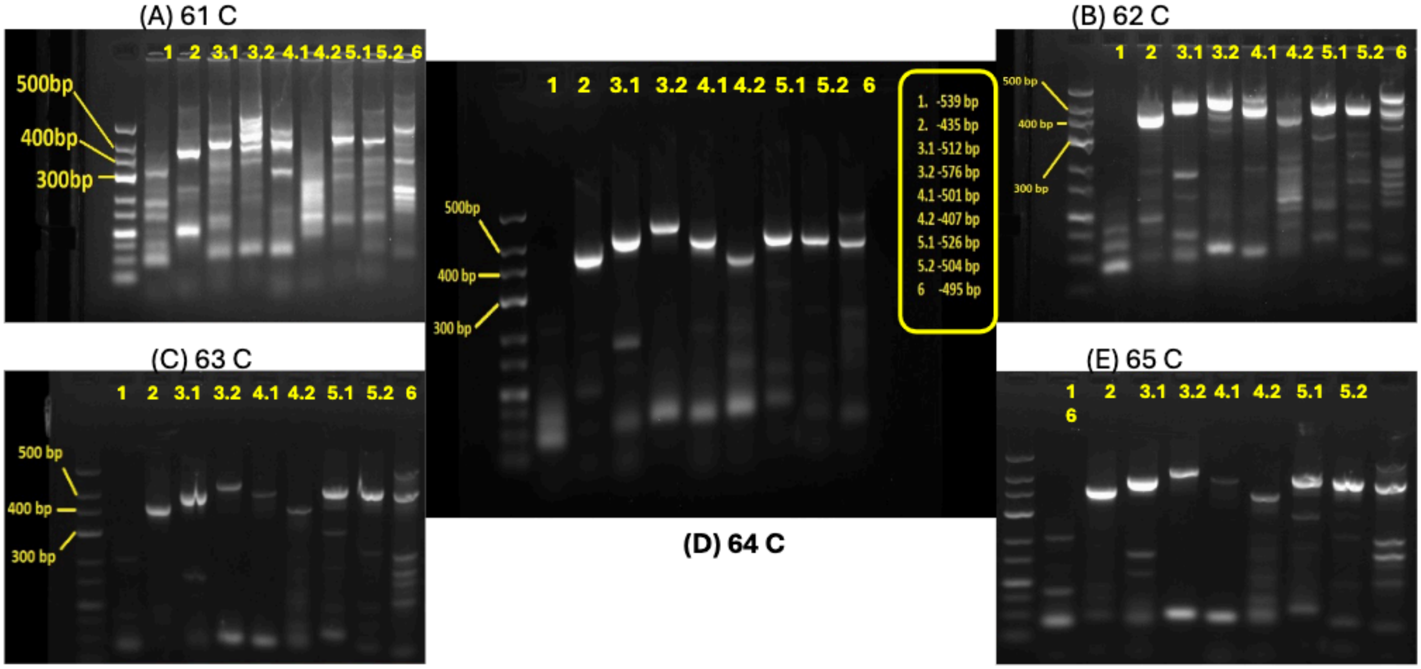

S Fig. 5: Optimization of NAT primers using gDNA as template. Annealing temperature was selected by performing PCR with gradual temperature increase. (A) Annealing temperature was 61C, (B) Annealing temperature was 62C, (C) Annealing temperature was 63C, (D) Annealing temperature was 64C, (E) Annealing temperature was 65C. Data showed that 64C temperature worked best than any other condition. Target band size (in bp) was shown in (D) inset.

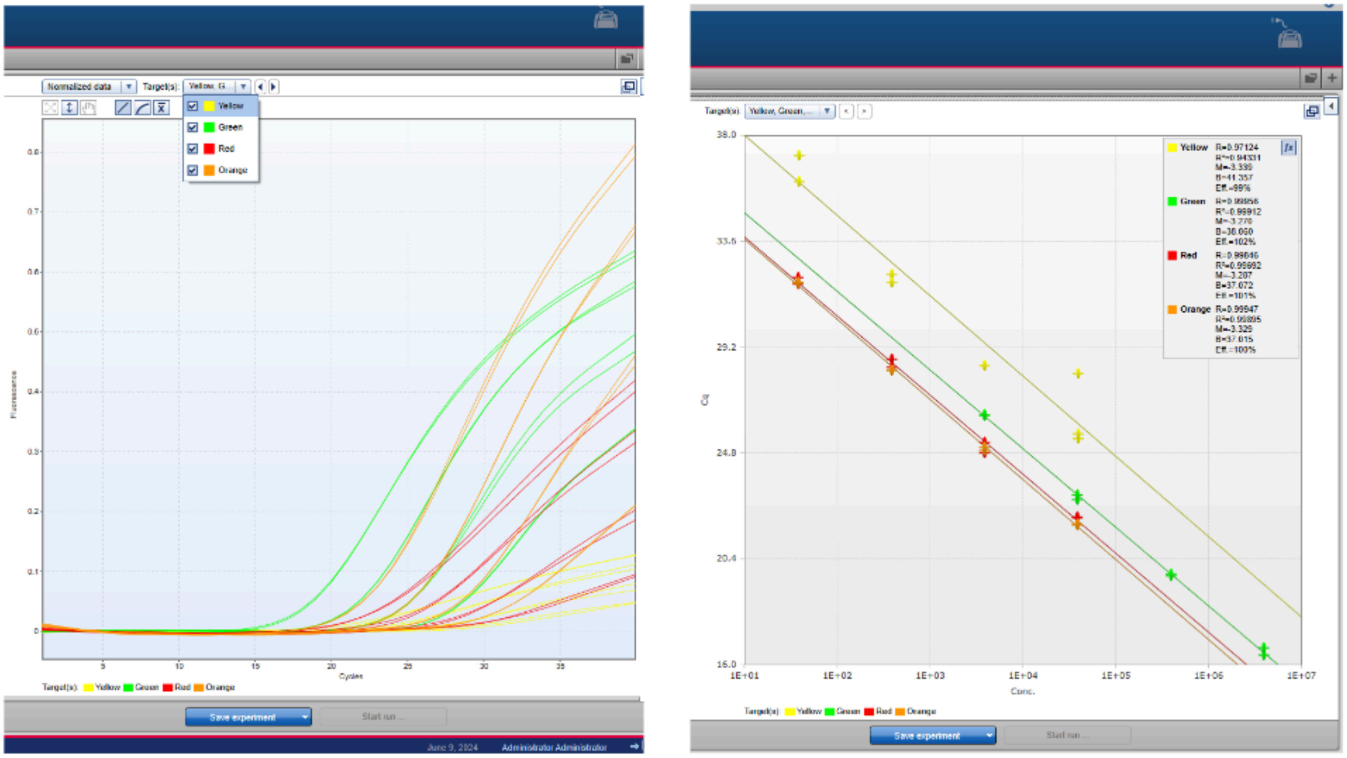

S Fig. 6: Q-Rex software snapshot of qPCR run and calibration curve preparation of qPCR template standards. Synthetic DNA was used as template for copy number calculation. Multiplex qPCR was used for quantifying total 4 targets at the same time (3 for NAT targets and 1 for HMGA1 mRNA target). (Left) qPCR runs of serially diluted standards. (Right) Calibration curves of all 4 templates from the single tube. PCR efficiency was 99-102%. By using y=mx+b equation, concentration (x) was calculated and converted to copy number.

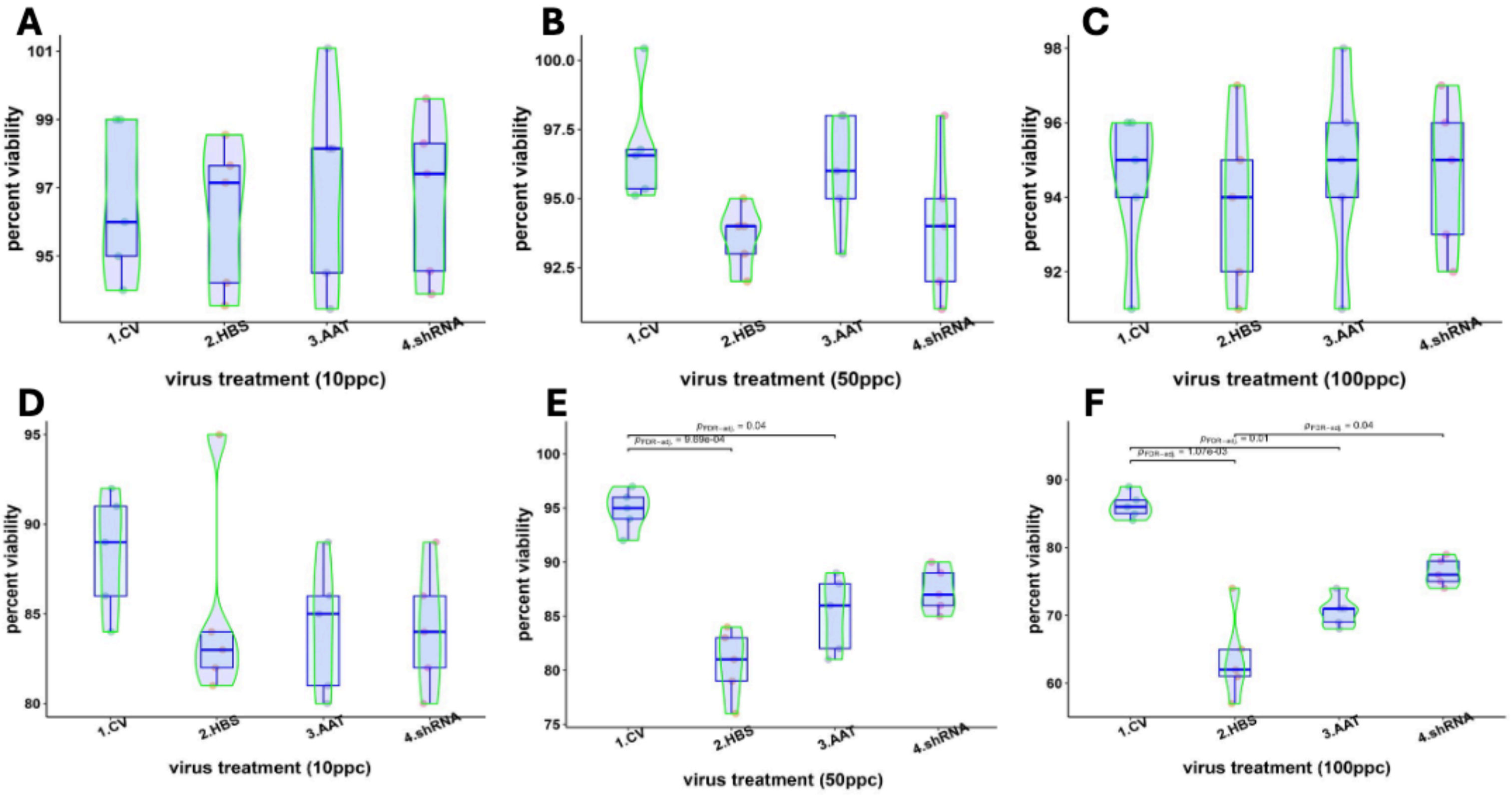

S Fig. 7: Viability assay data of ZR-75 on day 1 and day 2 at 3 different viral doses: (A) 10ppc on day 1, (B) 50ppc on day 1, (C) 100ppc on day 1, (D) 10ppc on day 2, (E) 50ppc on day 2, (F) 100ppc on day 2. There was no difference in viability on day 1 at any viral doses (A,B,C), however, on day 2 only HBS and AAT virus treated cells showed lower viability ($p<0.01$) at higher doses (E,F). CV=Control virus, HBS=Hyper binding containing virus, AAT=AAT transcript containing virus, shRNA= shRNA transcript containing virus.

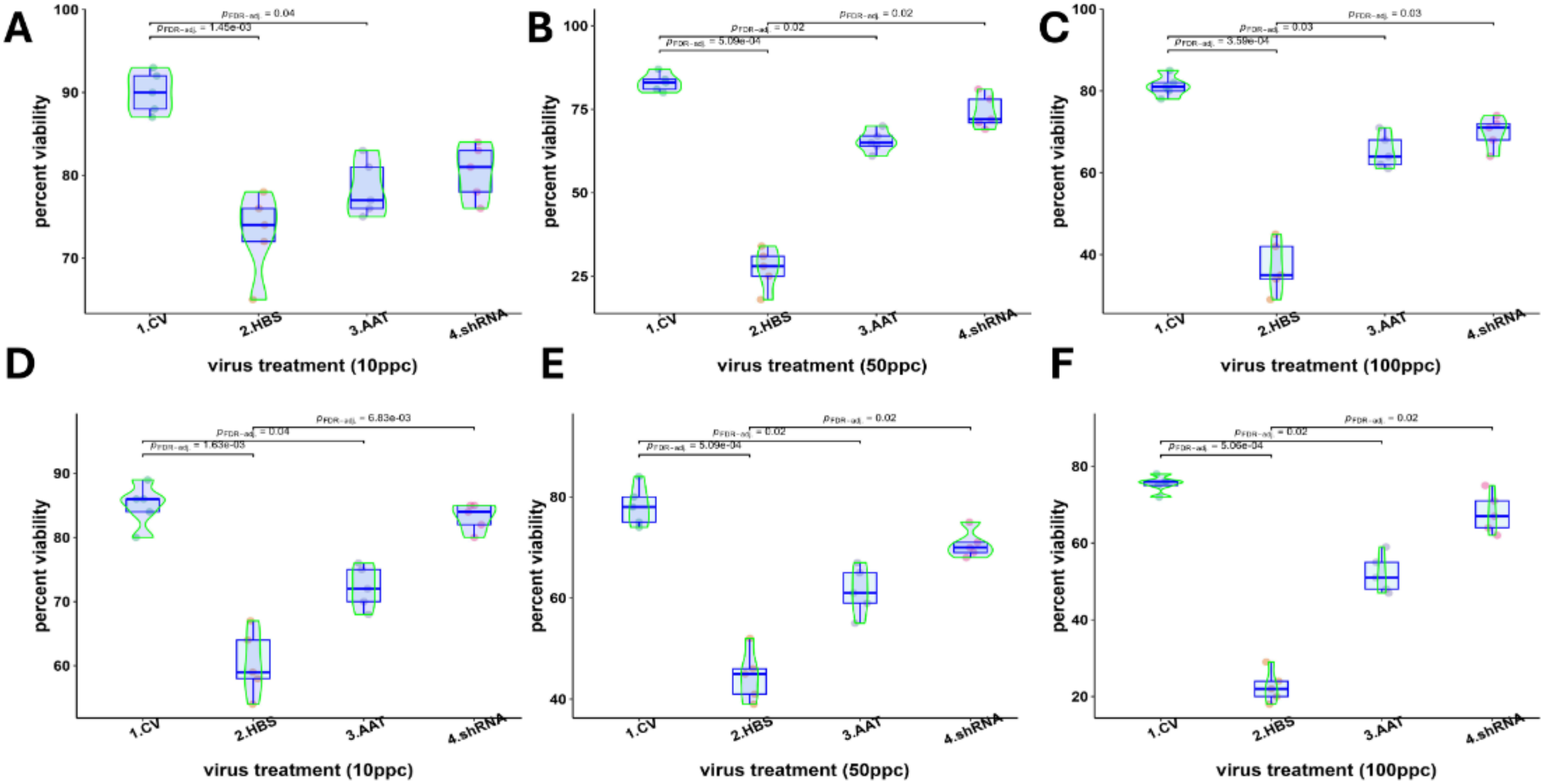

S Fig. 8: Viability assay data of ZR-75 on day 3 and day 4 at 3 different viral doses: (A) 10ppc on day 3, (B) 50ppc on day 3, (C) 100ppc on day 3, (D) 10ppc on day 4, (E) 50ppc on day 4, (F) 100ppc on day 4. On day 3 and 4, both HBS and AAT virus treated cells showed significantly lower viability at all doses (A-F). CV=Control virus, HBS=Hyper binding containing virus, AAT=AAT transcript containing virus, shRNA= shRNA transcript containing virus.

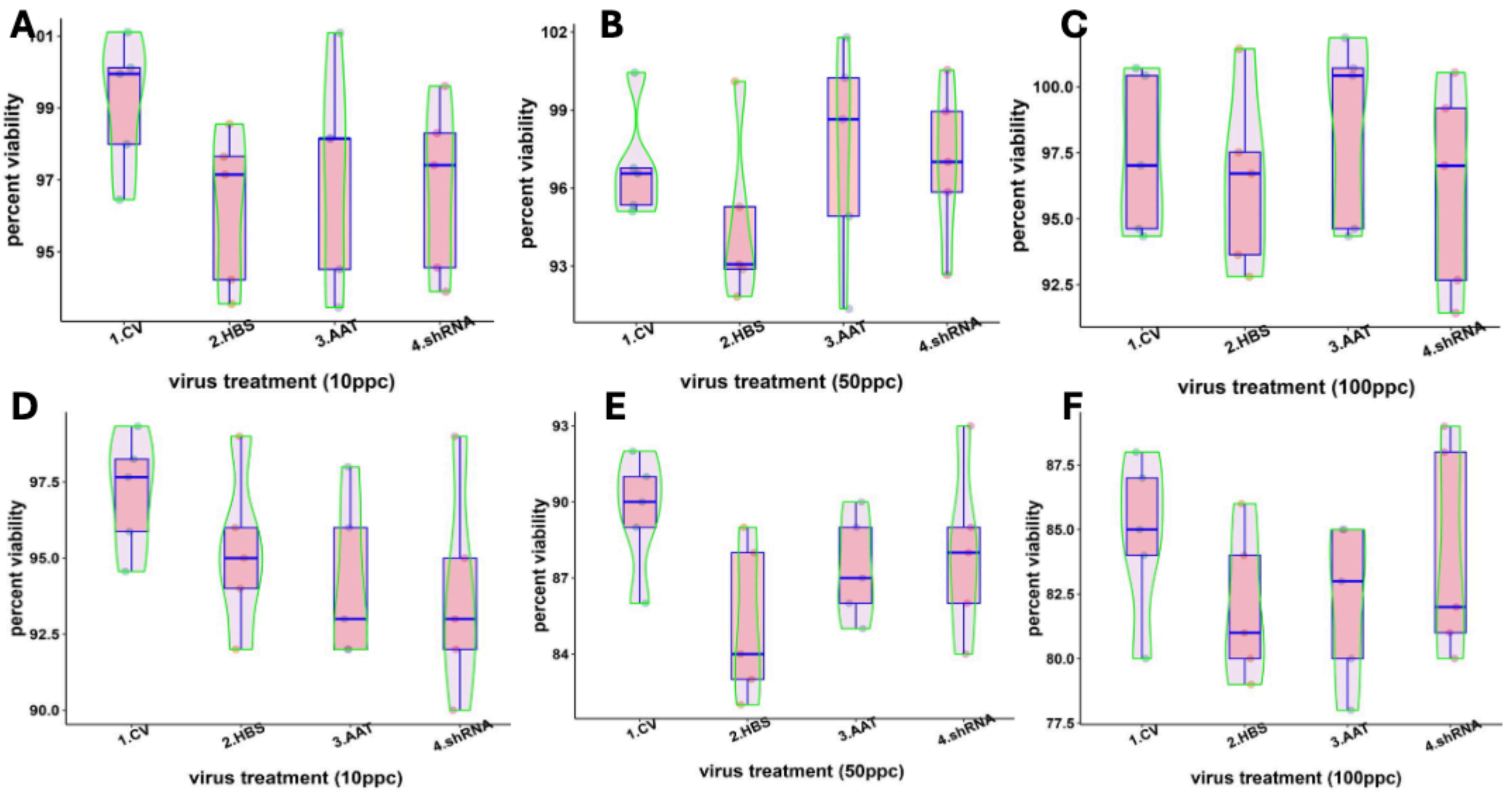

S Fig. 9: Viability assay data of MiaPaCa-2 on day 1 and day 2 at 3 different viral doses: (A) 10ppc on day 1, (B) 50ppc on day 1, (C) 100ppc on day 1, (D) 10ppc on day 2, (E) 50ppc on day 2, (F) 100ppc on day 2. There was no difference in viability on day 1 and day 2 at any viral doses (A-F). CV=Control virus, HBS=Hyper binding containing virus, AAT=AAT transcript containing virus, shRNA= shRNA transcript containing virus.

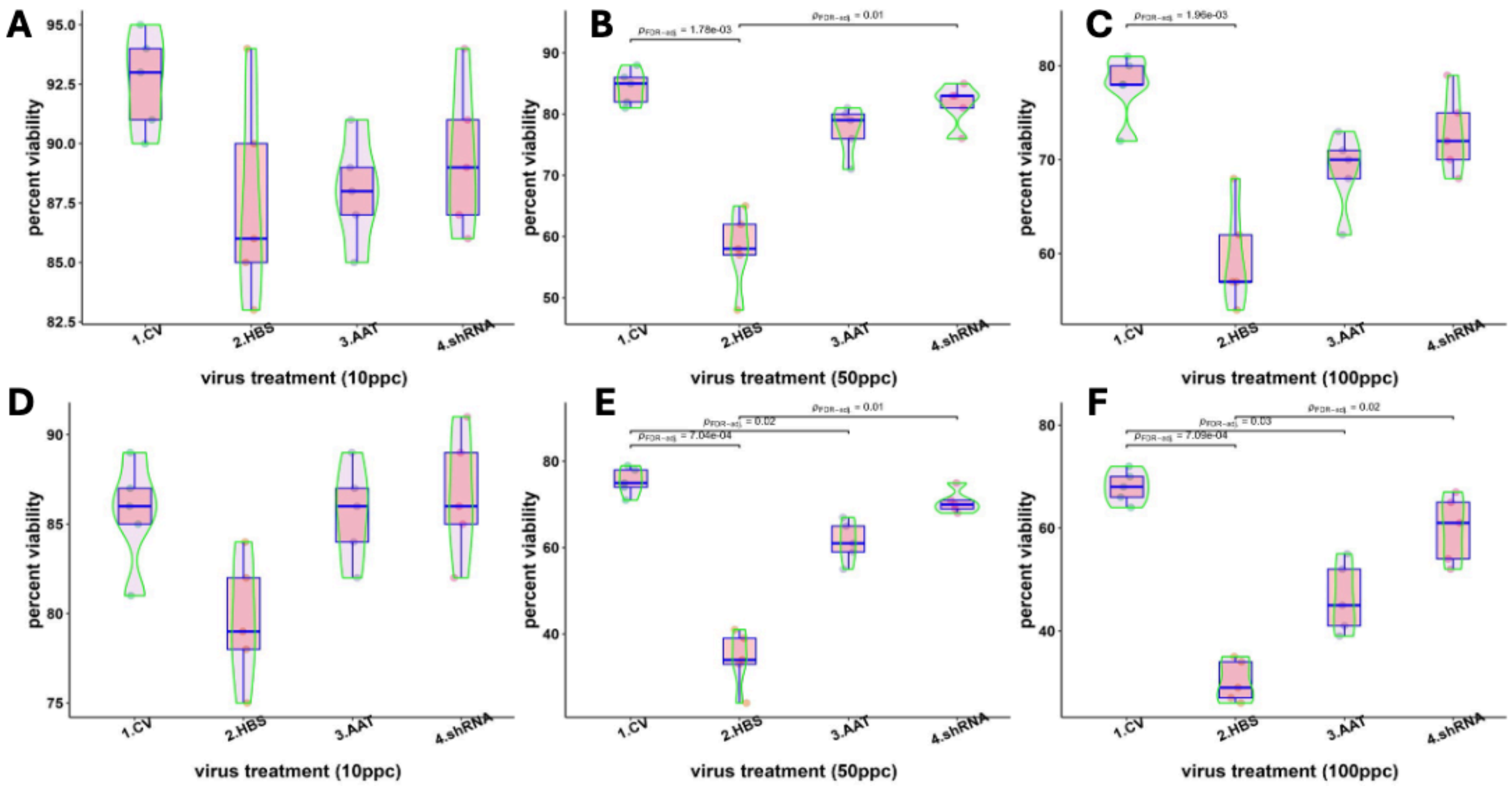

S Fig. 10: Viability assay data of MiaPaCa-2 on day 3 and day 4 at 3 different viral doses: (A) 10ppc on day 3, (B) 50ppc on day 3, (C) 100ppc on day 3, (D) 10ppc on day 4, (E) 50ppc on day 4, (F) 100ppc on day 4. HBS infected cells (day 3,4) and AAT infected cells (day 4) at 50 and 100ppc showed significantly lower viability. CV=Control virus, HBS=Hyper binding containing virus, AAT=AAT transcript containing virus, shRNA= shRNA transcript containing virus.

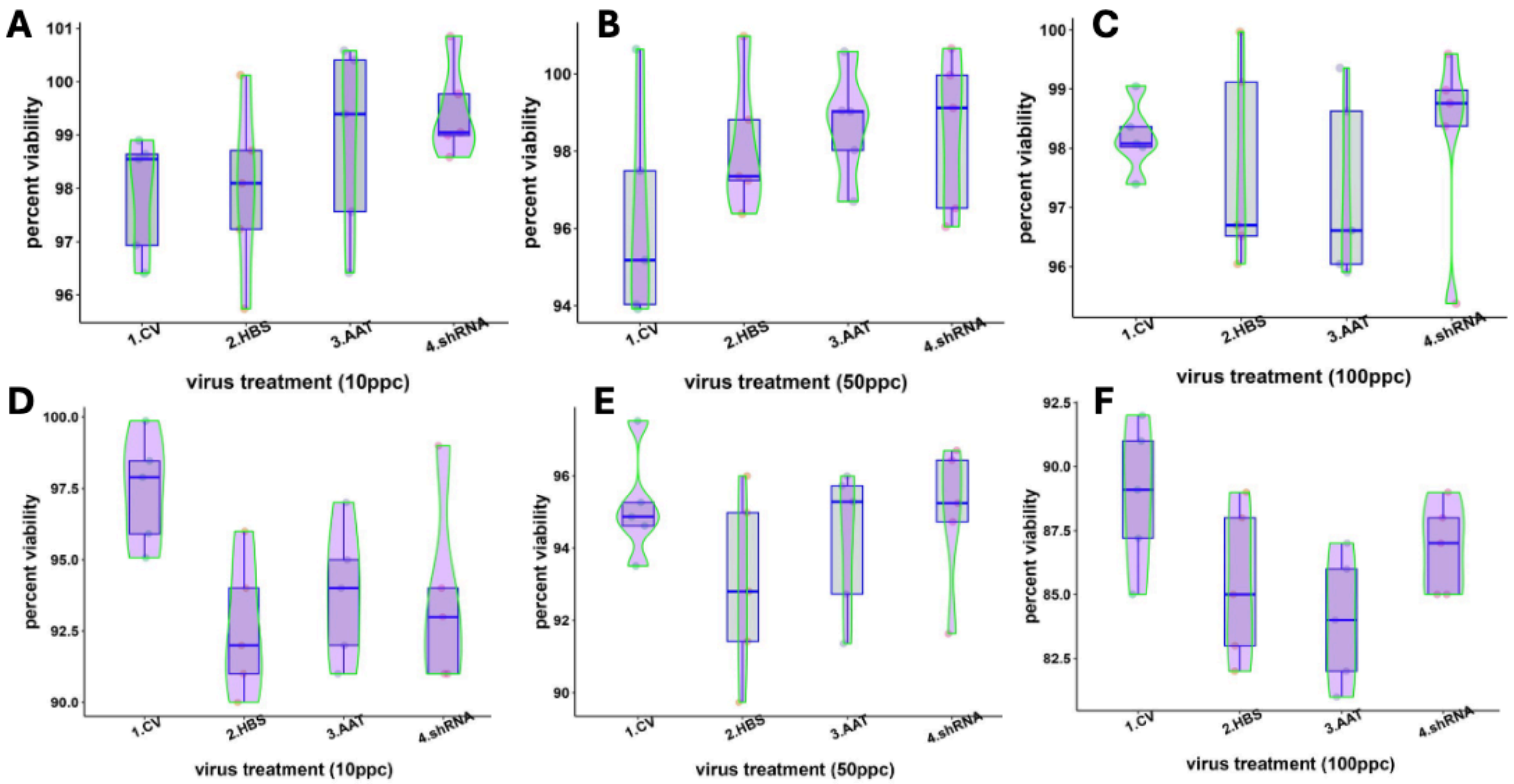

S Fig. 11: Viability assay data of BxPC-3 on day 1 and day 2 at 3 different viral doses: (A) 10ppc on day 1, (B) 50ppc on day 1, (C) 100ppc on day 1, (D) 10ppc on day 2, (E) 50ppc on day 2, (F) 100ppc on day 2. There was no difference in viability on day 1 and day 2 at any viral doses (A-F). CV=Control virus, HBS=Hyper binding containing virus, AAT=AAT transcript containing virus, shRNA= shRNA transcript containing virus.

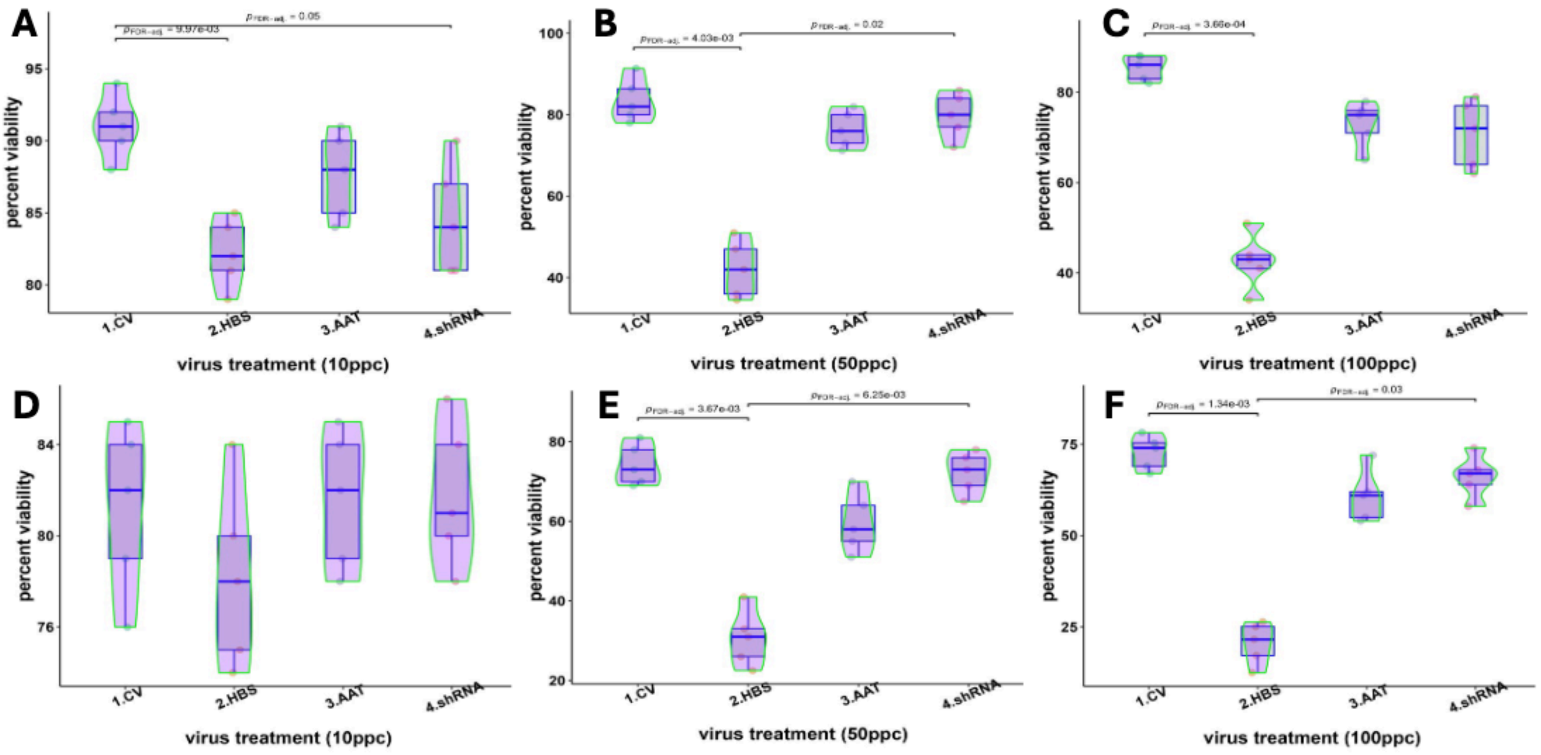

S Fig. 12: Viability assay data of BxPC-3 on day 3 and day 4 at 3 different viral doses: (A) 10ppc on day 3, (B) 50ppc on day 3, (C) 100ppc on day 3, (D) 10ppc on day 4, (E) 50ppc on day 4, (F) 100ppc on day 4. HBS infected cells (day 3,4) and AAT infected cells (day 4) at 50 and 100ppc showed significantly lower viability. CV=Control virus, HBS=Hyper binding containing virus, AAT=AAT transcript containing virus, shRNA= shRNA transcript containing virus.

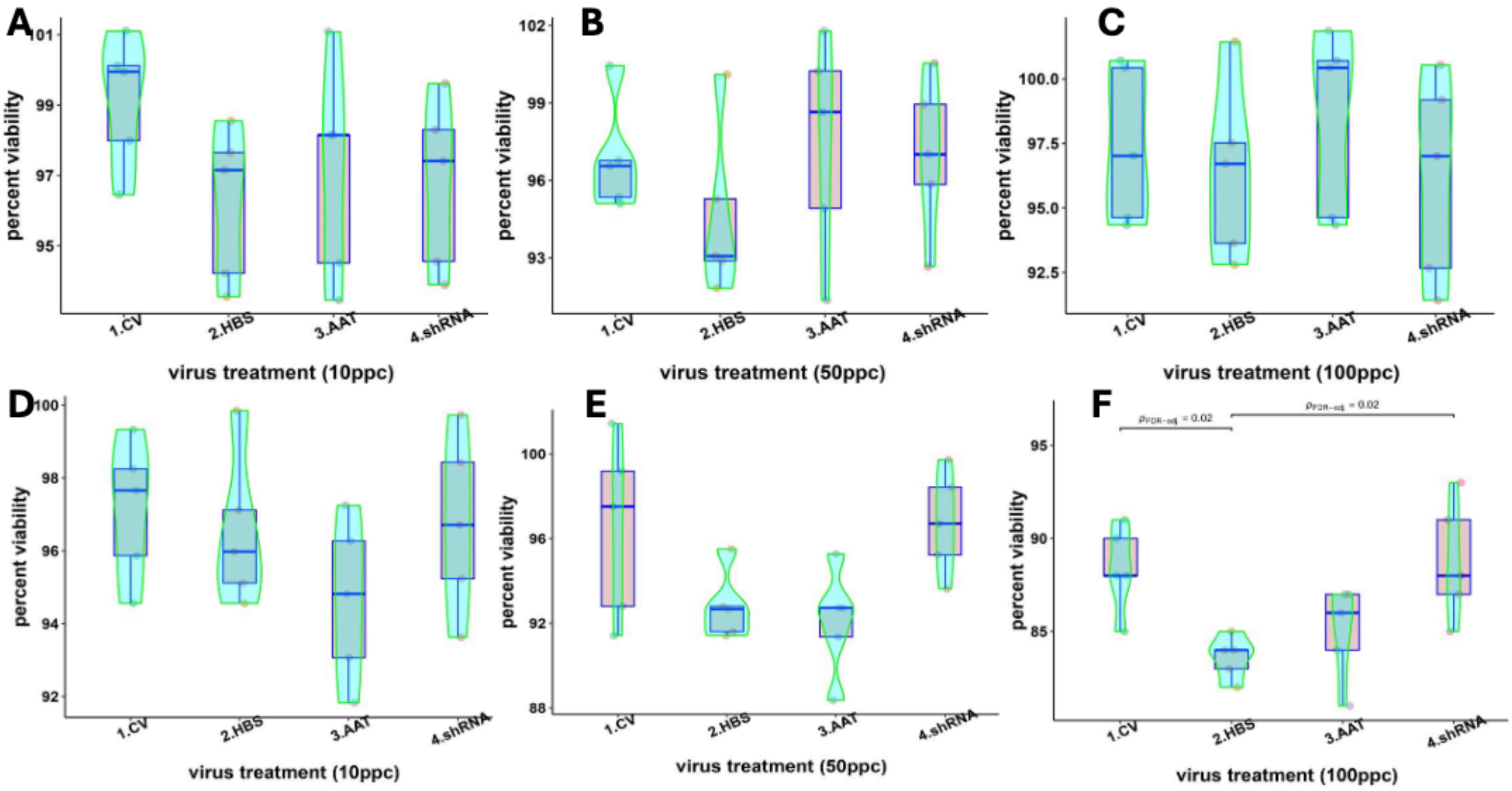

S Fig. 13: Viability assay data of PANC-1 on day 1 and day 2 at 3 different viral doses: (A) 10ppc on day 1, (B) 50ppc on day 1, (C) 100ppc on day 1, (D) 10ppc on day 2, (E) 50ppc on day 2, (F) 100ppc on day 2. HBS treated cells showed significant reduction in viability on day 2 at 100ppc (F). CV=Control virus, HBS=Hyper binding containing virus, AAT=AAT transcript containing virus, shRNA= shRNA transcript containing virus.

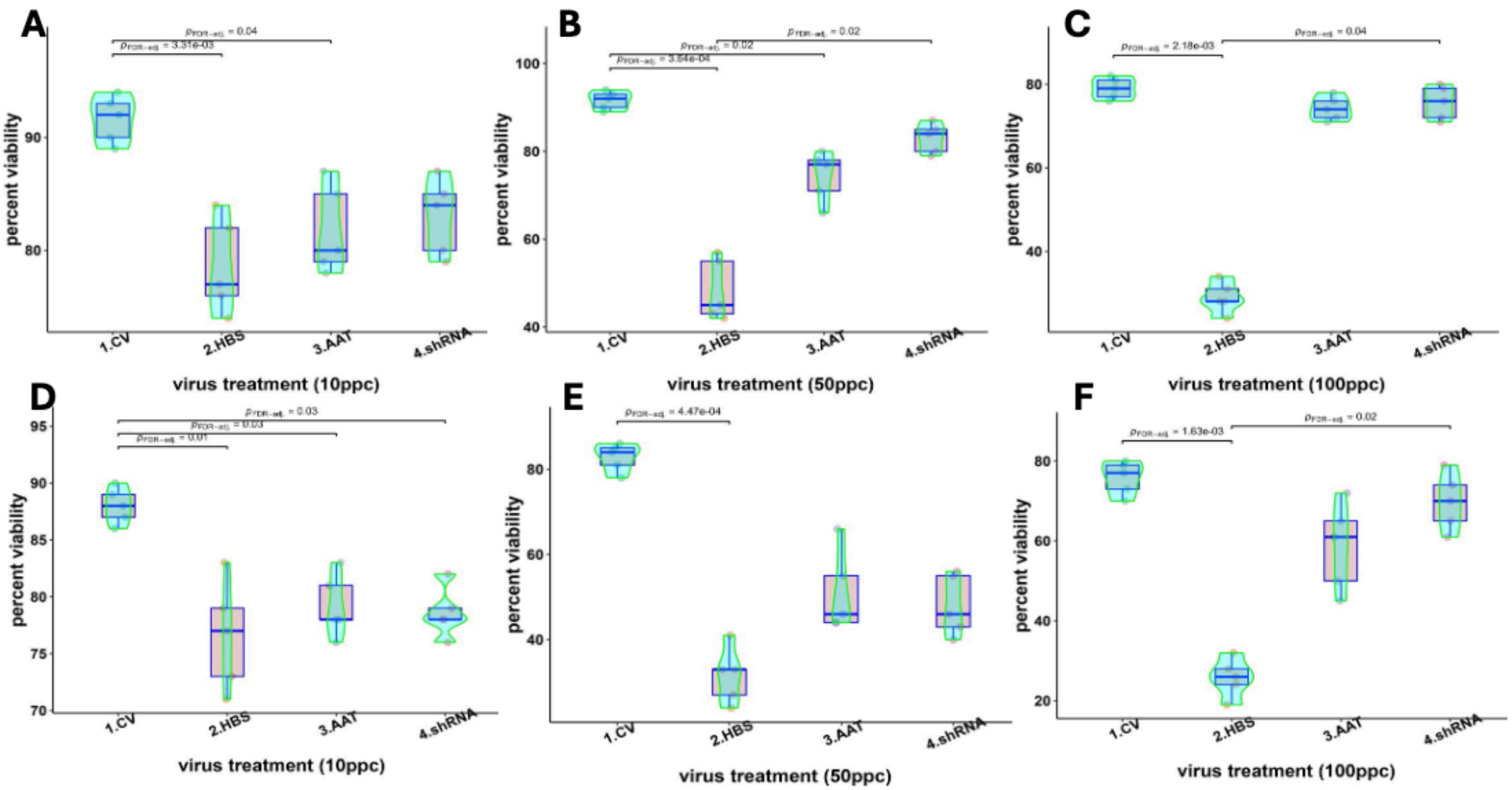

S Fig. 14: Viability assay data of PANC-1 on day 3 and day 4 at 3 different viral doses: (A) 10ppc on day 3, (B) 50ppc on day 3, (C) 100ppc on day 3, (D) 10ppc on day 4, (E) 50ppc on day 4, (F) 100ppc on day 4. HBS infected cells (A-F) and AAT infected cells (A,B,D) showed significantly lower viability at different viral doses.

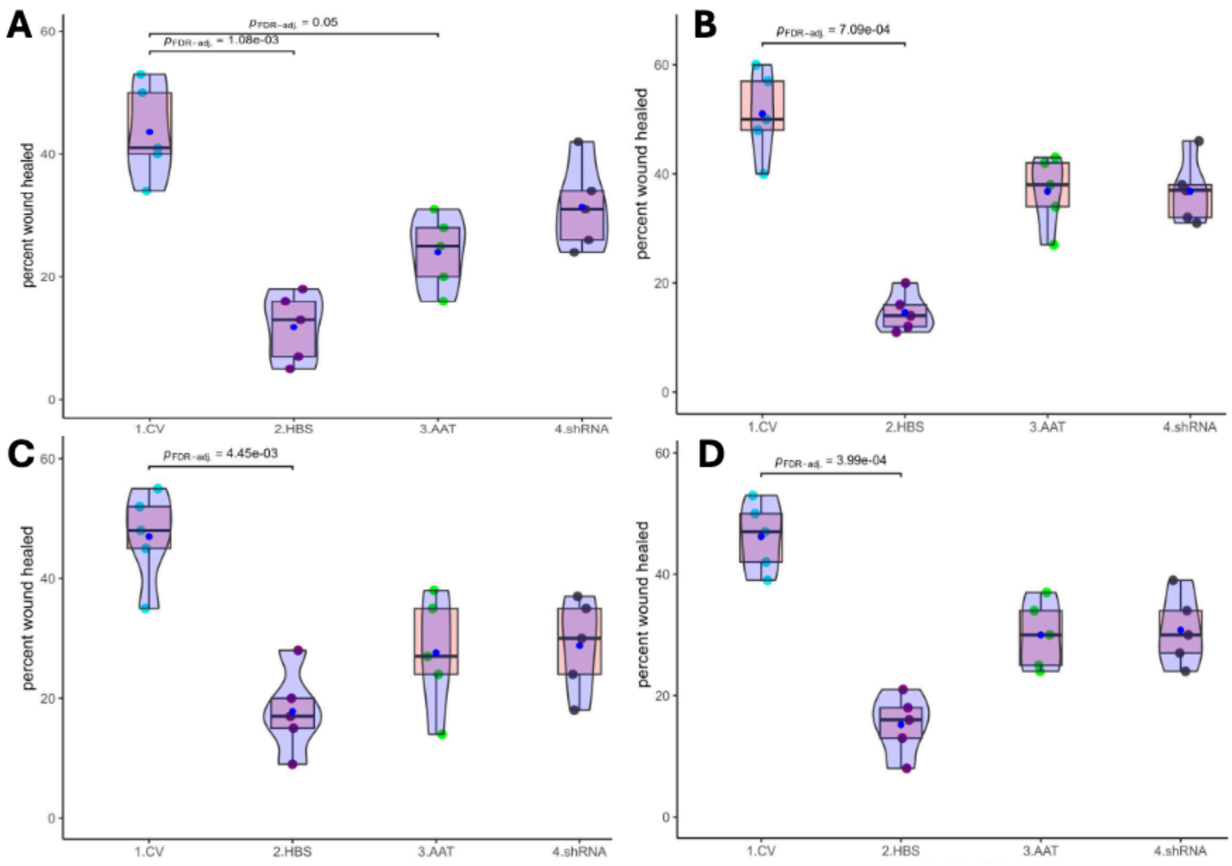

S Fig. 15: Wound healing assay at 24 hour: (A) ZR-75, (B) MiaPaCa-2, (C) BxPC-3, (D) PANC-1 at 50ppc. HBS infected all cell lines (A-D) showed significantly lower healing property (p<0.001) than other viruses. For ZR-75, AAT virus treated cells also showed significant reduction in healing at p=0.05. CV=Control virus, HBS=Hyper binding containing virus, AAT=AAT transcript containing virus, shRNA= shRNA transcript containing virus.

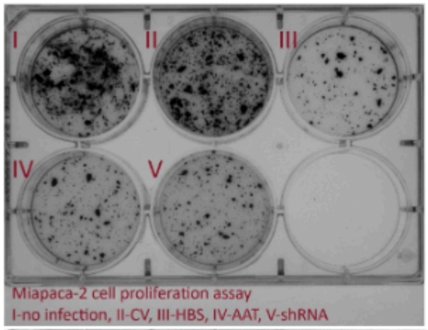

A

S Fig. 16A: Soft-agar cell proliferation assay of MiaPaCa-2 at 50ppc.
I-Cells without viral infection,
II-Control virus infection,
III-HBS virus infection,
IV-AAT virus infection,
V-shRNA virus infection.
Data showed that all engineered adeno viral vectors showed similar effect to prohibit cell proliferation to make colonies.

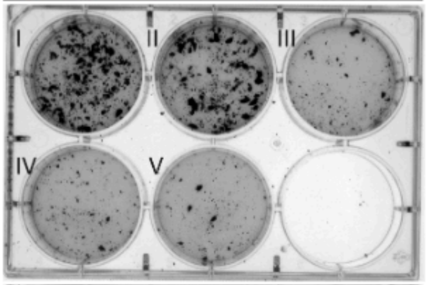

B

S Fig. 16B: Soft-agar cell proliferation assay of BxPC-3 at 50ppc. All engineered viral vectors showed almost equal effect on prohibiting cell proliferation (III,IV,V).

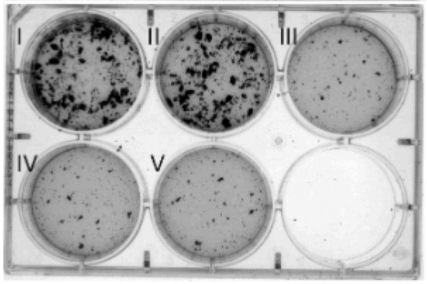

C

S Fig. 16C: Soft-agar cell proliferation assay of PANC-1 at 50ppc. All engineered viral vectors showed almost equal effect on prohibiting cell proliferation (III,IV,V).

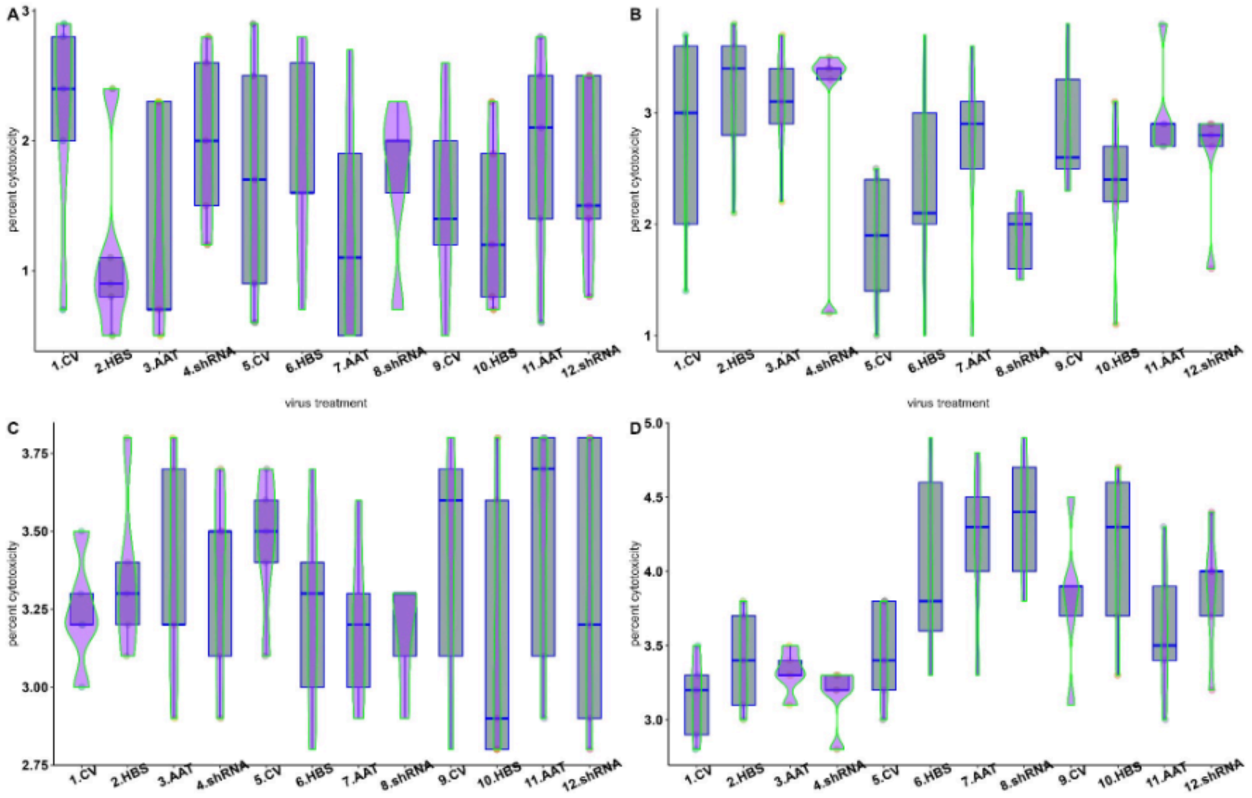

S Fig. 17: Cytotoxicity assay of ZR-75 at different doses: 10ppc (1-4), 50ppc (5-8), 100ppc (9-12) on day 1 (A), day 2(B), day 3 (C), and day 4 (D). Data showed that there was no cytotoxicity effect due to viral infection in any viral dose from day 1 to day 4. CV=Control virus, HBS=Hyper binding containing virus, AAT=AAT transcript containing virus, shRNA= shRNA transcript containing virus.

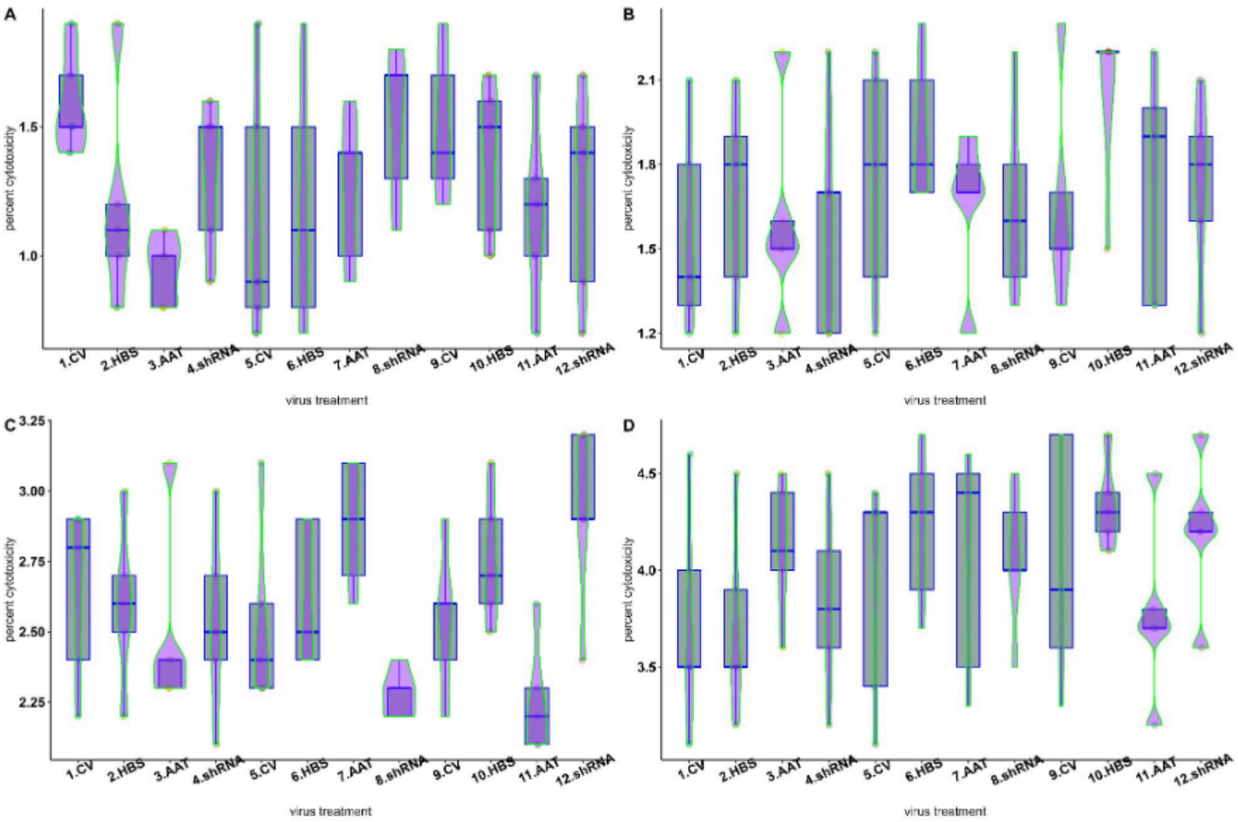

S Fig. 18: Cytotoxicity assay of MiaPaCa-2 at different doses: 10ppc (1-4), 50ppc (5-8), 100ppc (9-12) on day 1 (A), day 2(B), day 3 (C), and day 4 (D). Data showed that there was no cytotoxicity effect due to viral infection in any viral dose from day 1 to day 4.

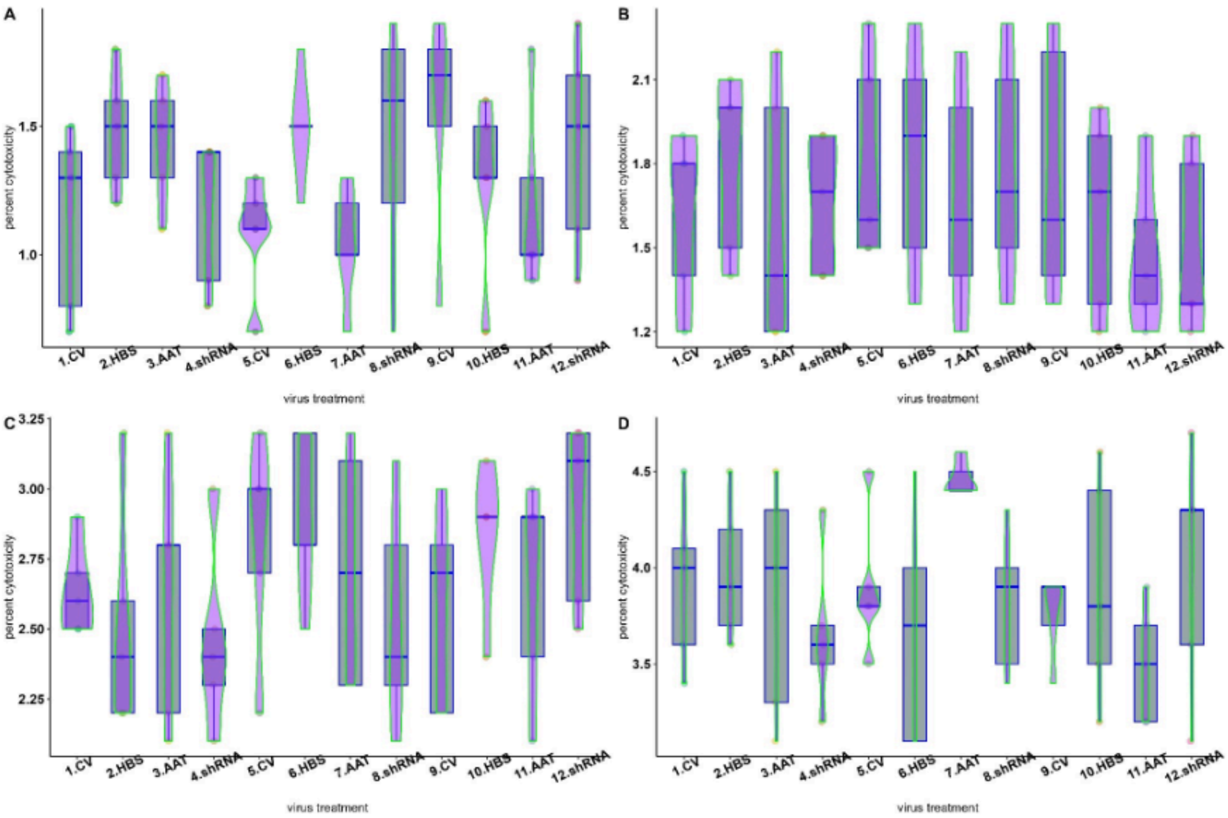

S Fig. 19: Cytotoxicity assay of BxPC-3 at different doses: 10ppc (1-4), 50ppc (5-8), 100ppc (9-12) on day 1 (A), day 2(B), day 3 (C), and day 4 (D). Data showed that there was no cytotoxicity effect due to viral infection in any viral dose from day 1 to day 4.

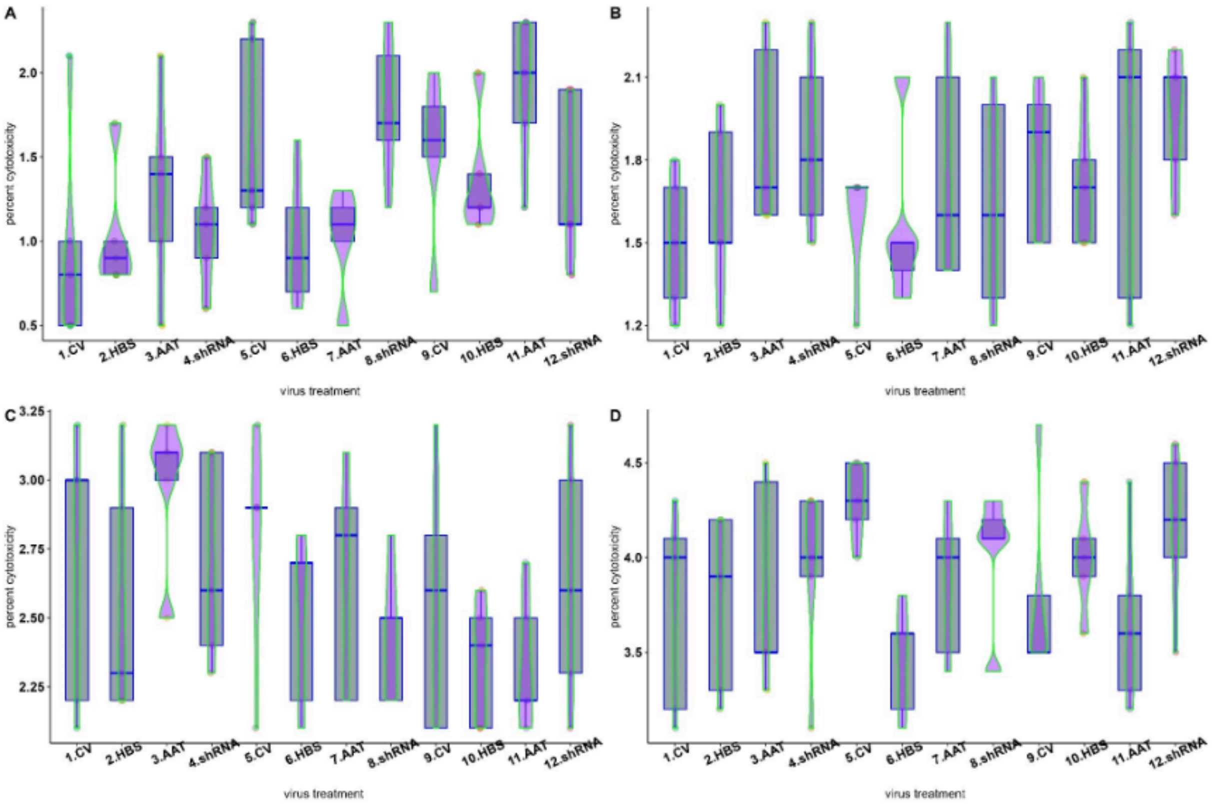

S Fig. 20: Cytotoxicity assay of PANC-1 at different doses: 10ppc (1-4), 50ppc (5-8), 100ppc (9-12) on day 1 (A), day 2(B), day 3 (C), and day 4 (D). Data showed that there was no cytotoxicity effect due to viral infection in any viral dose from day 1 to day 4.

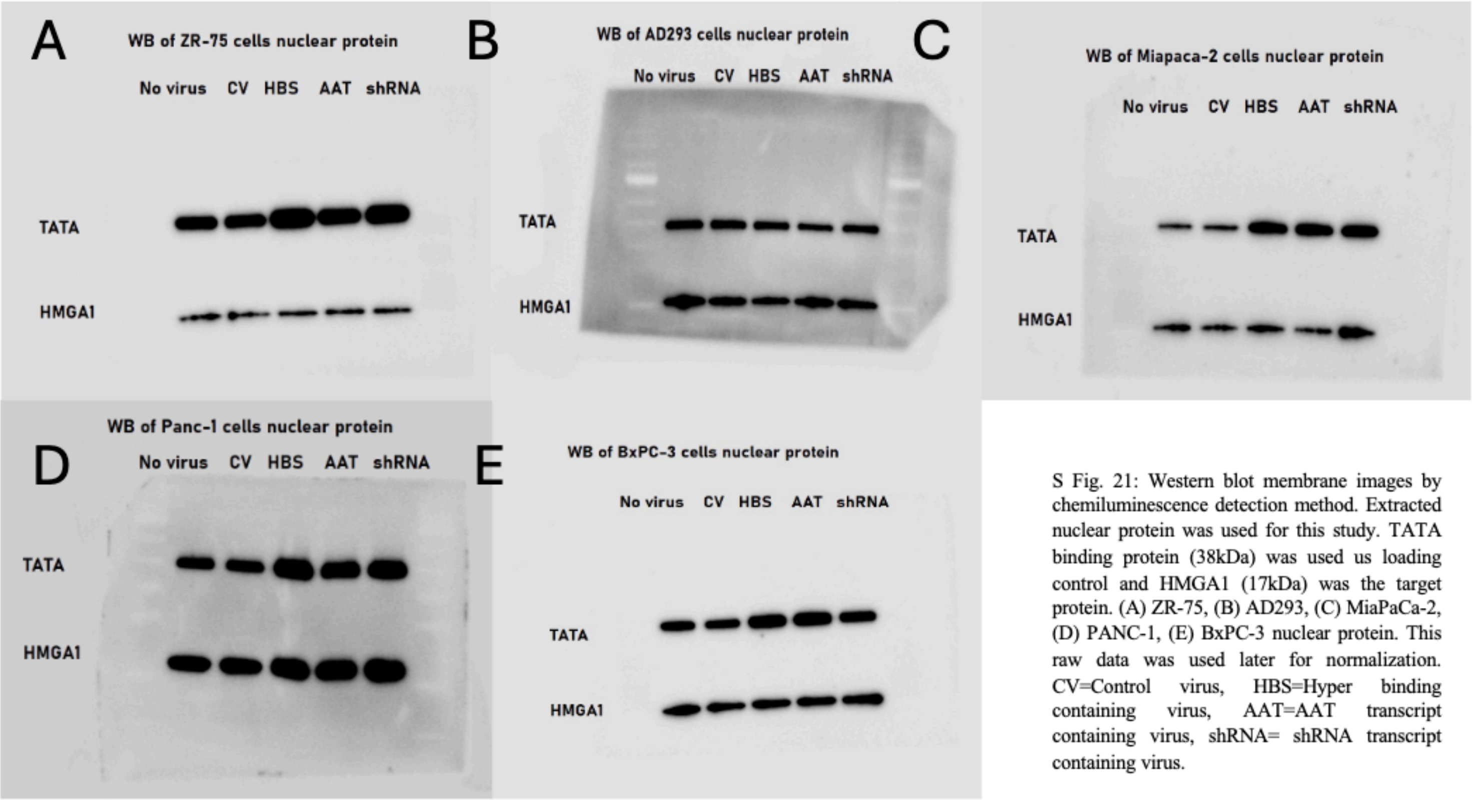

S Fig. 21: Western blot membrane images by chemiluminescence detection method. Extracted nuclear protein was used for this study. TATA binding protein (38kDa) was used us loading control and HMGA1 (17kDa) was the target protein. (A) ZR-75, (B) AD293, (C) MiaPaCa-2, (D) PANC-1, (E) BxPC-3 nuclear protein. This raw data was used later for normalization. CV=Control virus, HBS=Hyper binding containing virus, AAT=AAT transcript containing virus, shRNA= shRNA transcript containing virus.

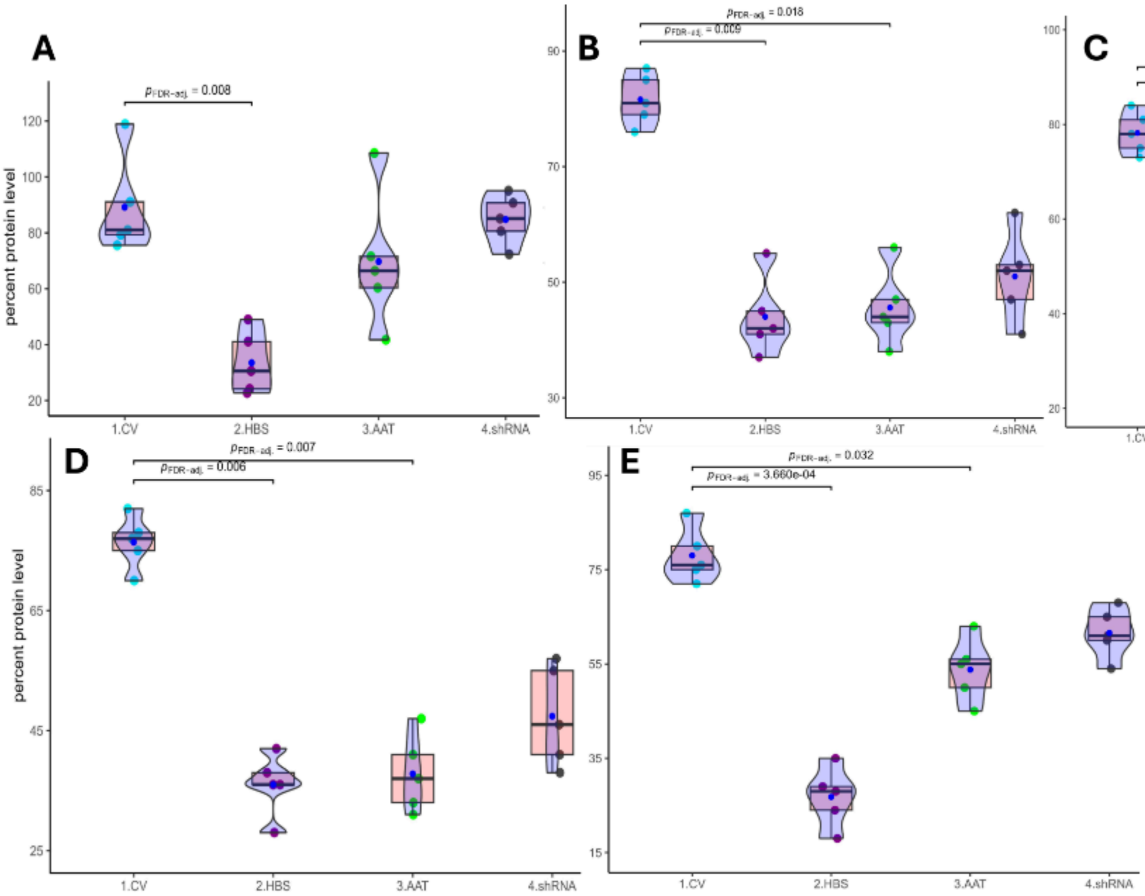

S Fig. 22: Statistical data analysis of normalized percent protein level (Y-axis) in different virus infected cells (X-axis). (A) ZR-75, (B) MiaPaCa-2, (C) BxPC-3, (D) PANC-1, (E) AD293. CV=Control virus, HBS=Hyper binding containing virus, AAT=AAT transcript containing virus, shRNA= shRNA transcript containing virus.

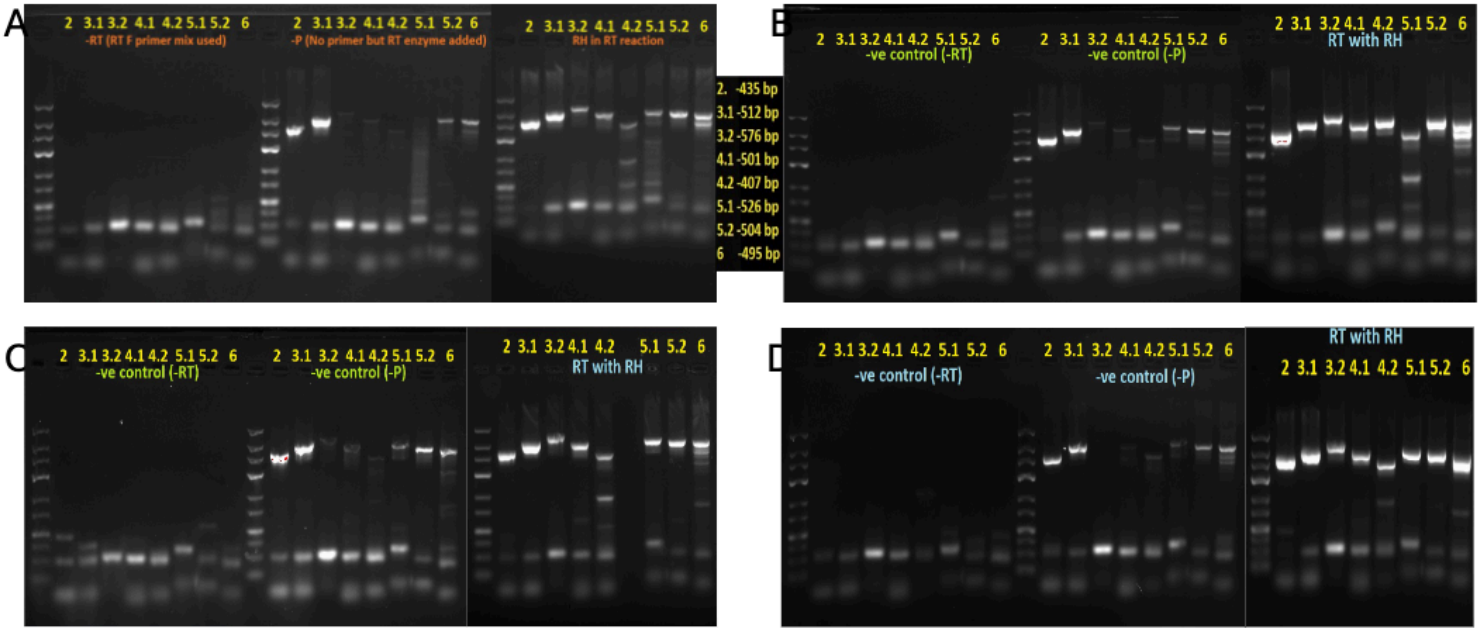

S Fig. 23: Detection of NATs from total RNA samples in different cancer cells and AD293. Total RNA was extracted from the cells followed by cDNA synthesis and PCR amplification with the NAT primers. (A) ZR-75, (B) MiaPaCa-2, (C) BxPC-3, (D) AD293. Column number indicated the position of the exon intron junctions. For all A,B,C,D, the left gel image indicated negative control (no RT enzyme), middle gel image indicated another negative control (no RT primer) and the right gel image indicated samples with all RT components. All target bands were separated by gel cut, purified and sequenced to confirm identity.

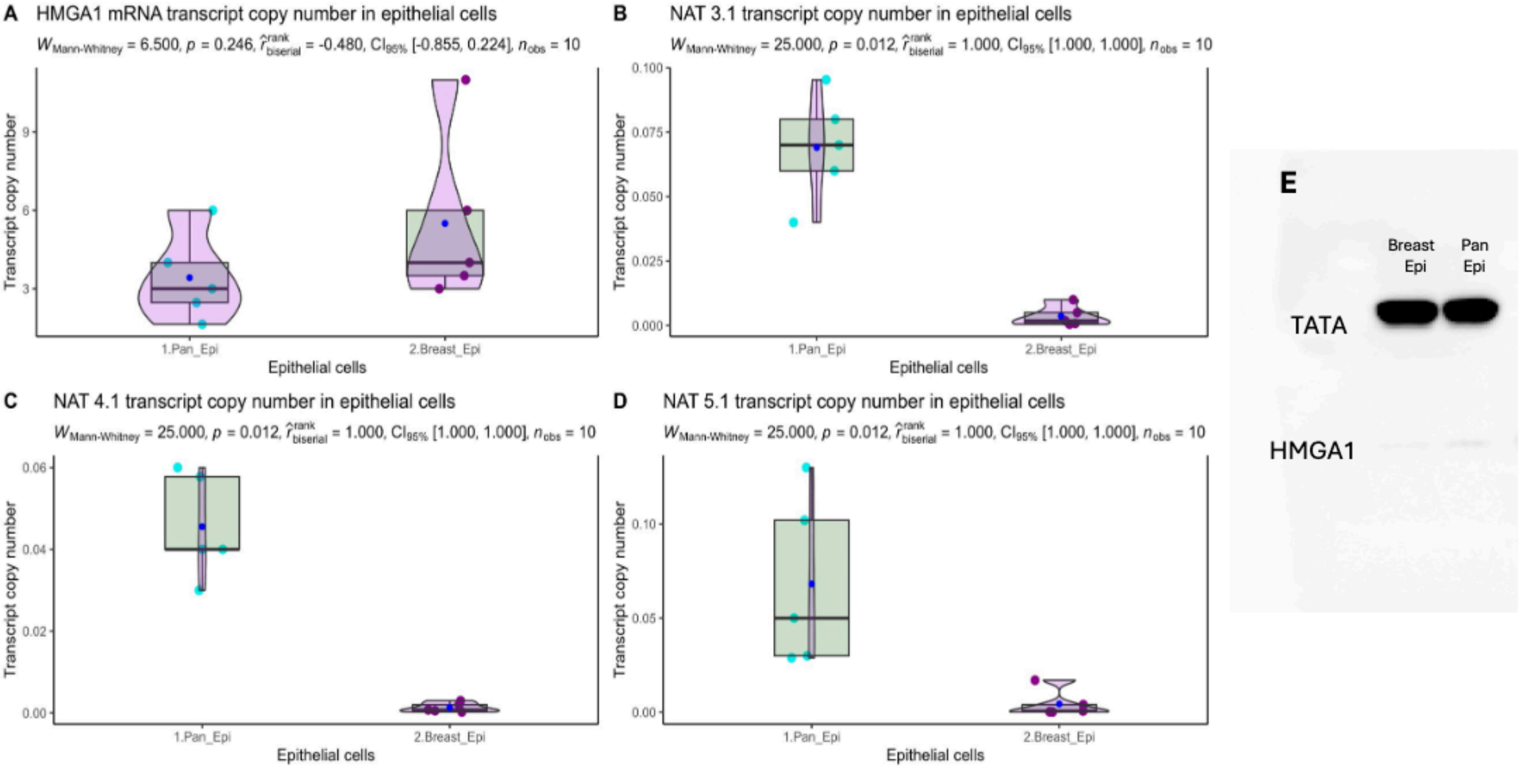

S Fig. 24: HMGA1 mRNA transcript and NAT transcripts copy number of pancreatic and breast epithelial cells. (A) HMGA1 mRNA copy number per cell (~3-6/cell), (B) NAT (3.1 position) copy number per cell (~0.01-0.08/cell), (C) NAT (4.1 position) copy number per cell (~0.01-0.06/cell), (D) NAT (5.1 position) copy number per cell (~0.01-0.15/cell). (E) Western blot data of breast and pancreatic healthy epithelial cells. TATA as loading control protein and HMGA1 protein was hardly detected indicating healthy human cells contain very low level of HMGA1 protein.

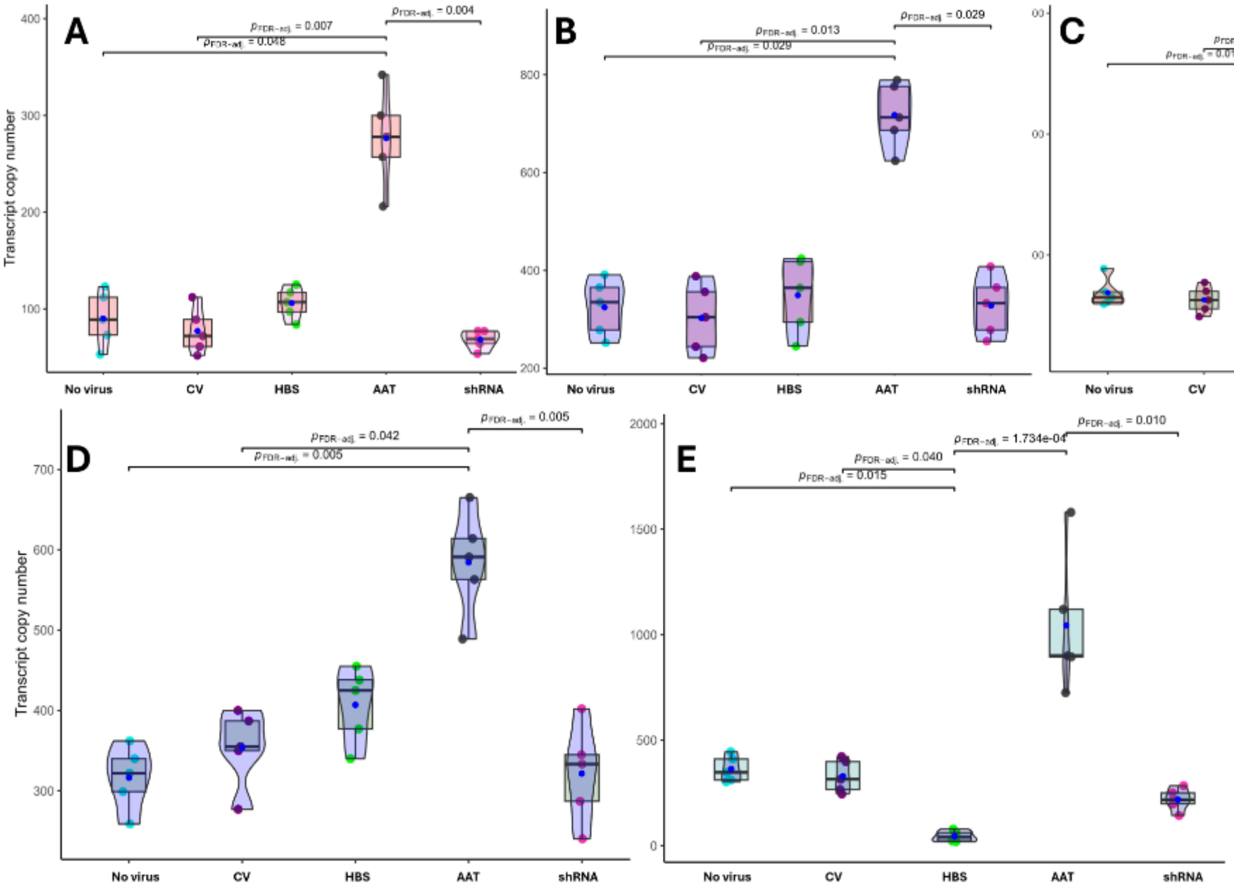

S Fig. 25: HMGA1 mRNA transcripts copy number of all cancer cells. (A) ZR-75, (B) MiaPaCa-2, (C) BxPC-3, (D) PANC-1, (E) AD-293. X axis showed all virus treated cell type (left to right): No virus infection, CV virus infected, HBS virus infected, AAT virus infected, shRNA virus infected. In case of no virus infected cancer cells, ZR-75 showed lowest mRNA transcripts copy number (~90/cell) than other cells (~300-400/cell).

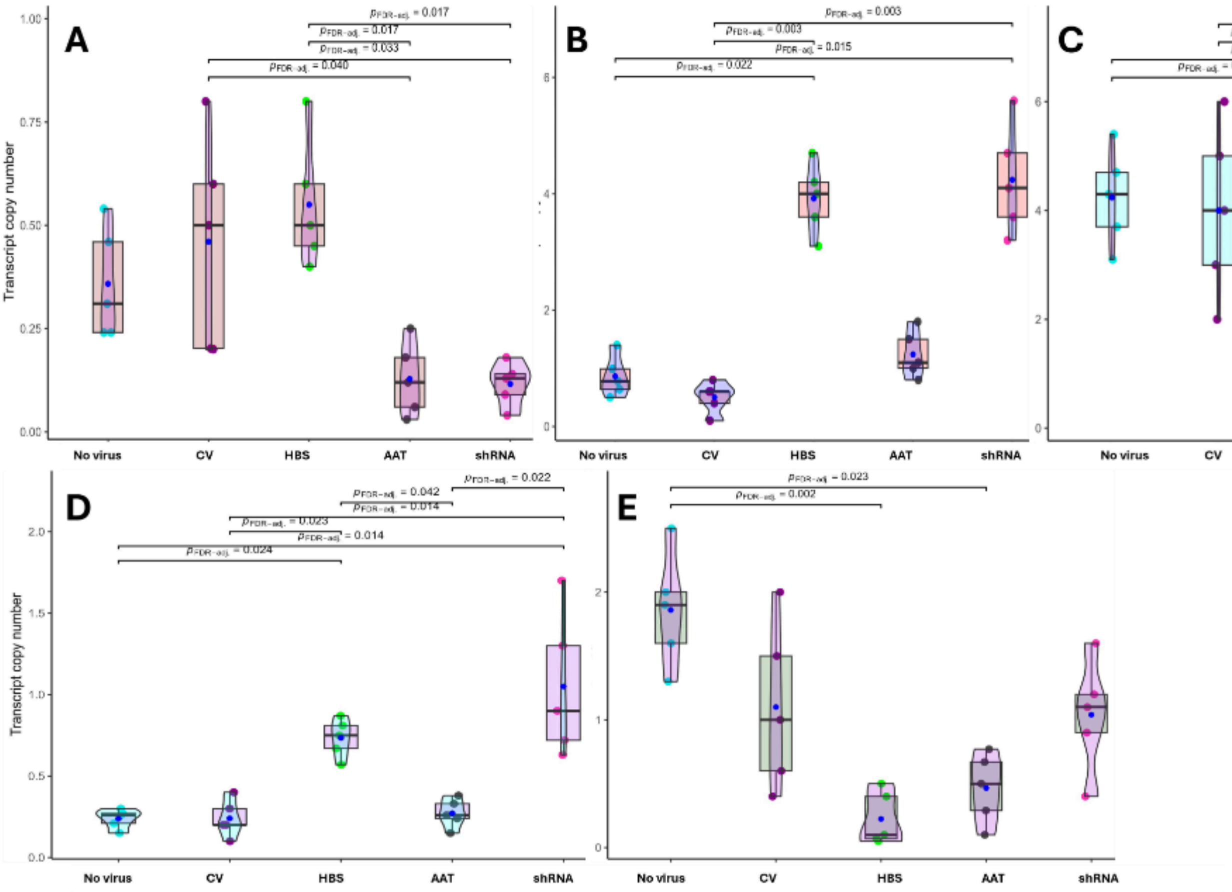

S Fig. 26: NAT transcripts copy number of all cancer cells at 3.1 position. (A) ZR-75, (B) MiaPaCa-2, (C) BxPC-3, (D) PANC-1, (E) AD-293.

X axis showed all virus treated cell type (left to right): No virus infection, CV virus infected, HBS virus infected, AAT virus infected, shRNA virus infected.

In case of no virus infected cancer cells, ZR-75 and PANC-1 showed lower copy number (~0.26/cell) than other cancers (~0.25-5/cell).

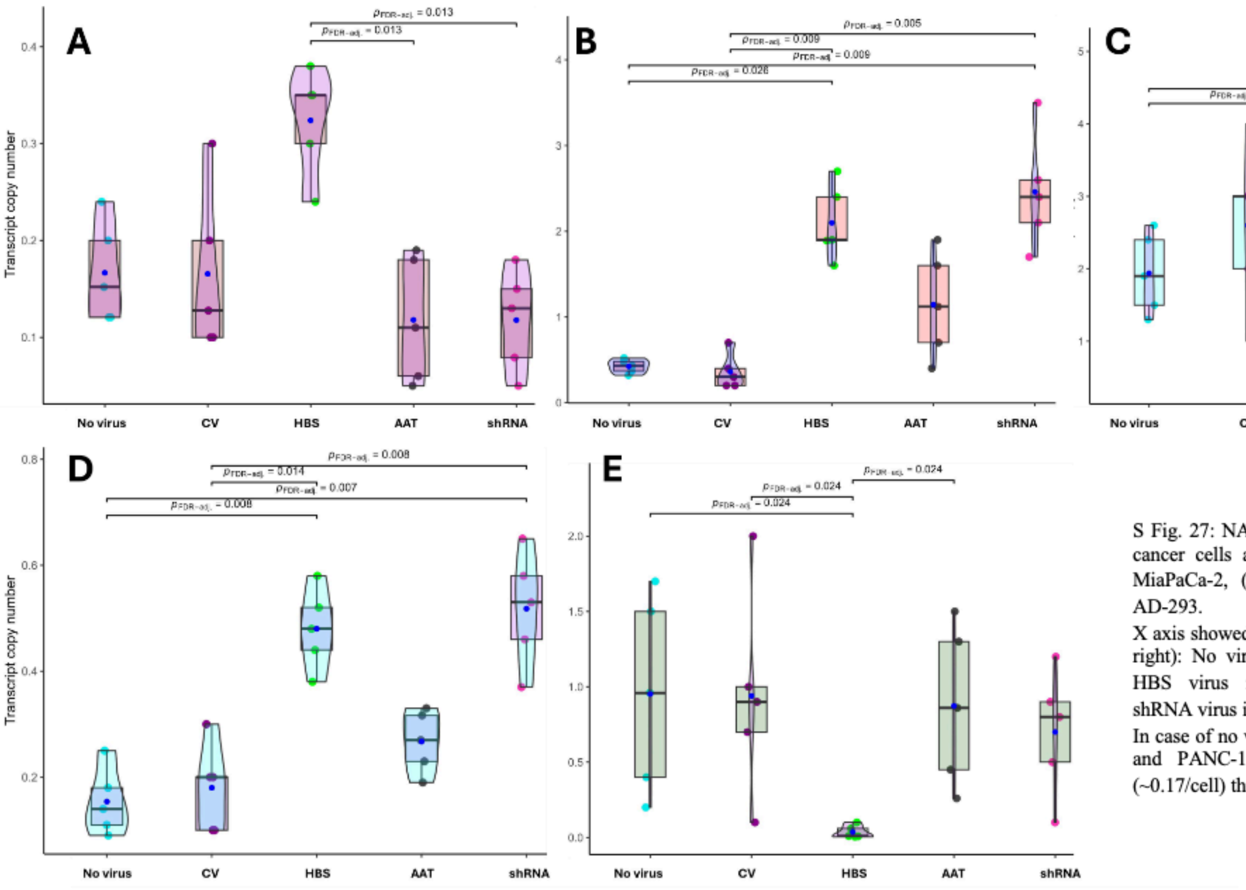

S Fig. 27: NAT transcripts copy number of all cancer cells at 4.1 position. (A) ZR-75, (B) MiaPaCa-2, (C) BxPC-3, (D) PANC-1, (E) AD-293.

X axis showed all virus treated cell type (left to right): No virus infection, CV virus infected, HBS virus infected, AAT virus infected, shRNA virus infected.

In case of no virus infected cancer cells, ZR-75 and PANC-1 showed lower copy number (~0.17/cell) than other cells (~0.5-2/cell).

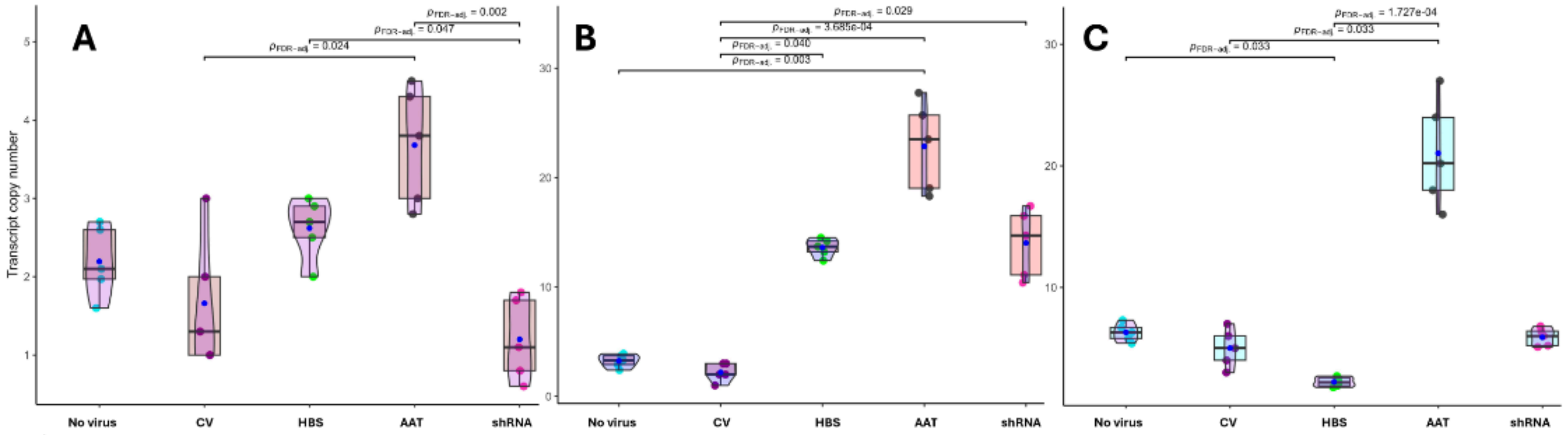

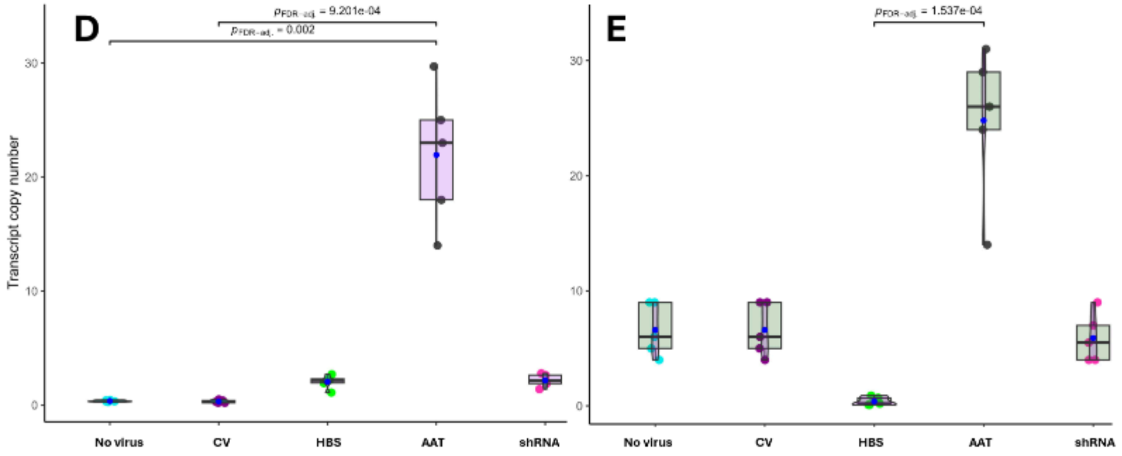

S Fig. 28: NAT transcripts copy number of all cancer cells at 5.1 position. (A) ZR-75, (B) MiaPaCa-2, (C) BxPC-3, (D) PANC-1, (E) AD-293.
X axis showed all virus treated cell type (left to right): No virus infection, CV virus infected, HBS virus infected, AAT virus infected, shRNA virus infected.
In case of no virus infected cancer cells, ZR-75 and PANC-1 showed lower copy number (~1-2/cell) than other cells (~4-8/cell).

S Fig. 29 to 50 contain similar graphical design and analysis, therefore, the common figure legends and description were explained below
(applicable for all figures from 29 to 50)

Regression analysis and effect size calculation using R programing language. (A) Regression analysis of one independent variable (either HMGA or NAT transcripts) presented in the left two columns and multiple independent variables (both HMGA and NAT) presented in the 3rd and 4th column (from left). Multiple variables without interaction was shown in 3rd column and with interaction was shown in 4th column. (B) Effect sizes calculation for without interaction model (3rd column). (C) Effect sizes calculation with interaction model (4th column).

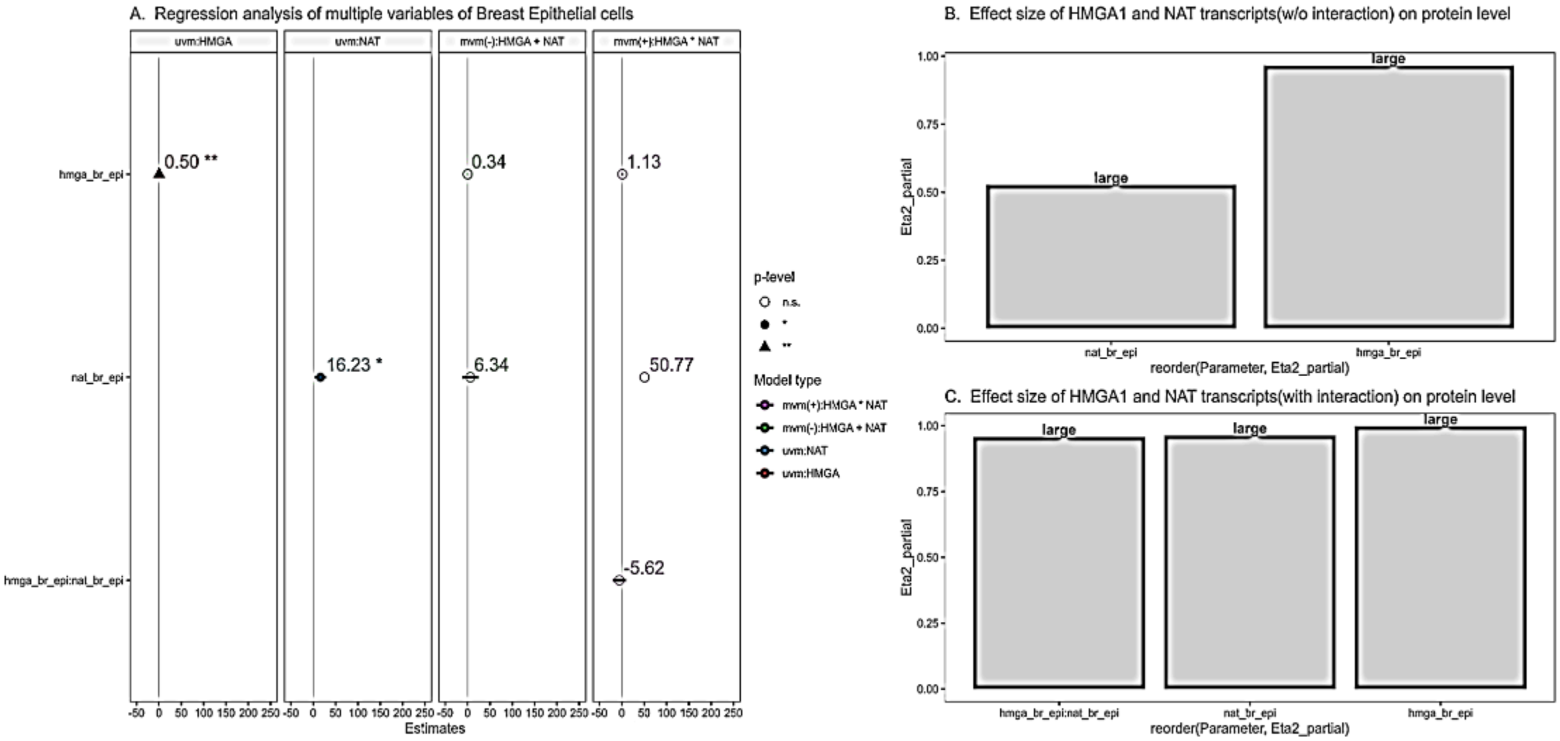

S Fig. 29: Regression analysis and effect size calculation of breast epithelial cells. According to AIC values, with interaction model works best for this cell line. Effect of HMGA1 and NAT transcripts and their interactions showed equal impact on maintaining low protein level (C).

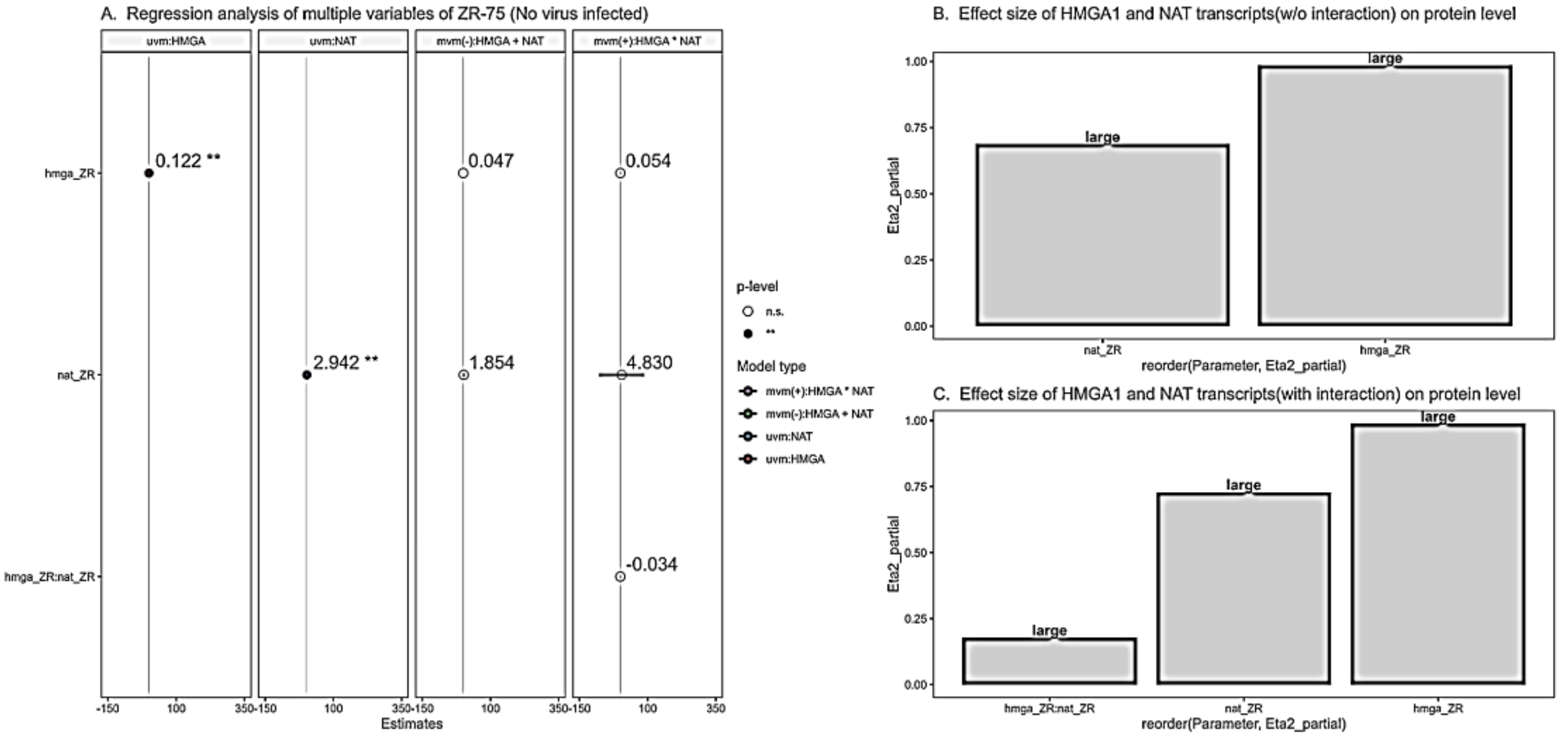

S Fig. 30: Regression analysis and effect size calculation of ZR-75 (no infection). According to AIC values, without interaction model seems better but due to VIF value interaction exists. Effects of HMGA1 and NAT interactions (C) showed much less impact on protein level than breast epithelial cells.

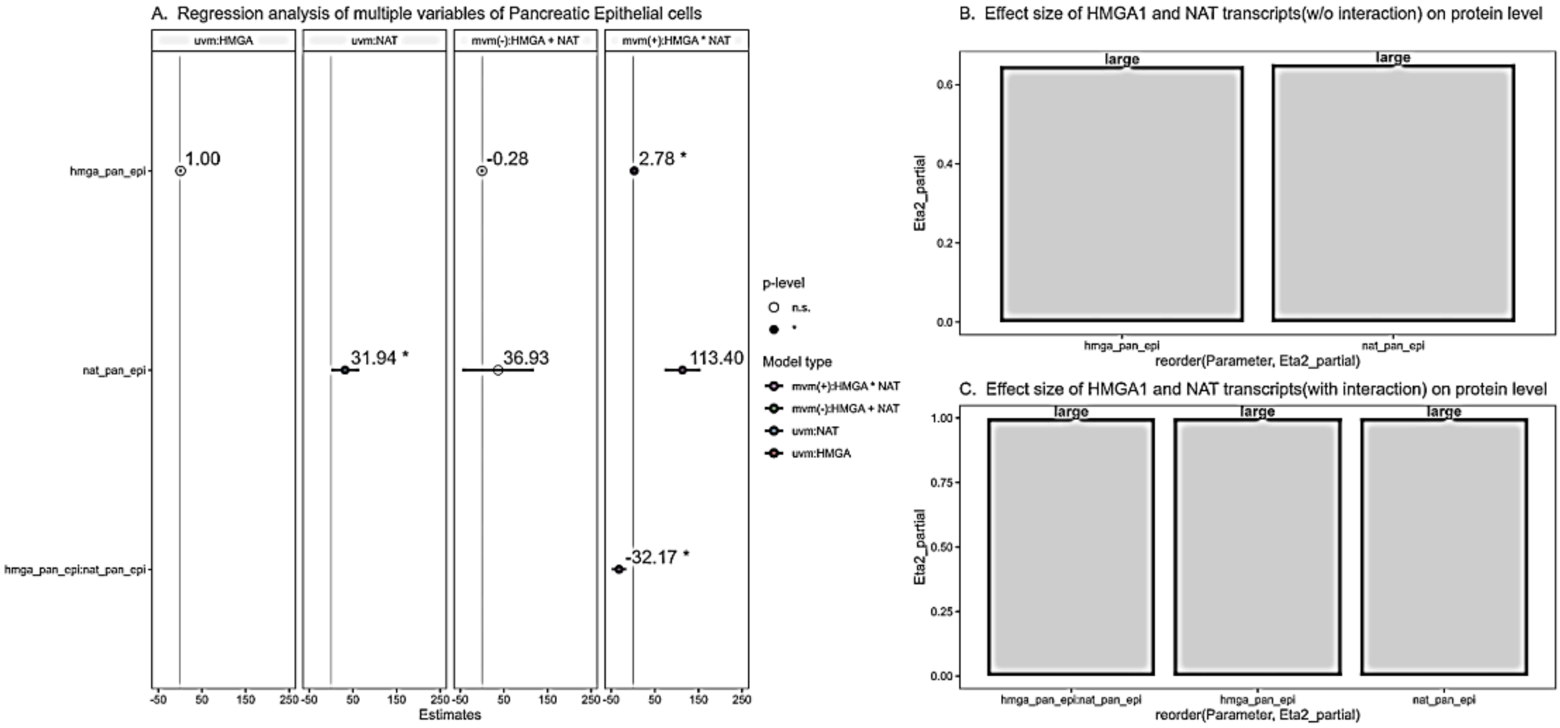

S Fig. 31: Regression analysis and effect size calculation of pancreatic epithelial cells. According to AIC values, with interaction model works best for this cell line. Effect of HMGA1 and NAT transcripts and their interactions showed equal impact on maintaining low protein level (C).

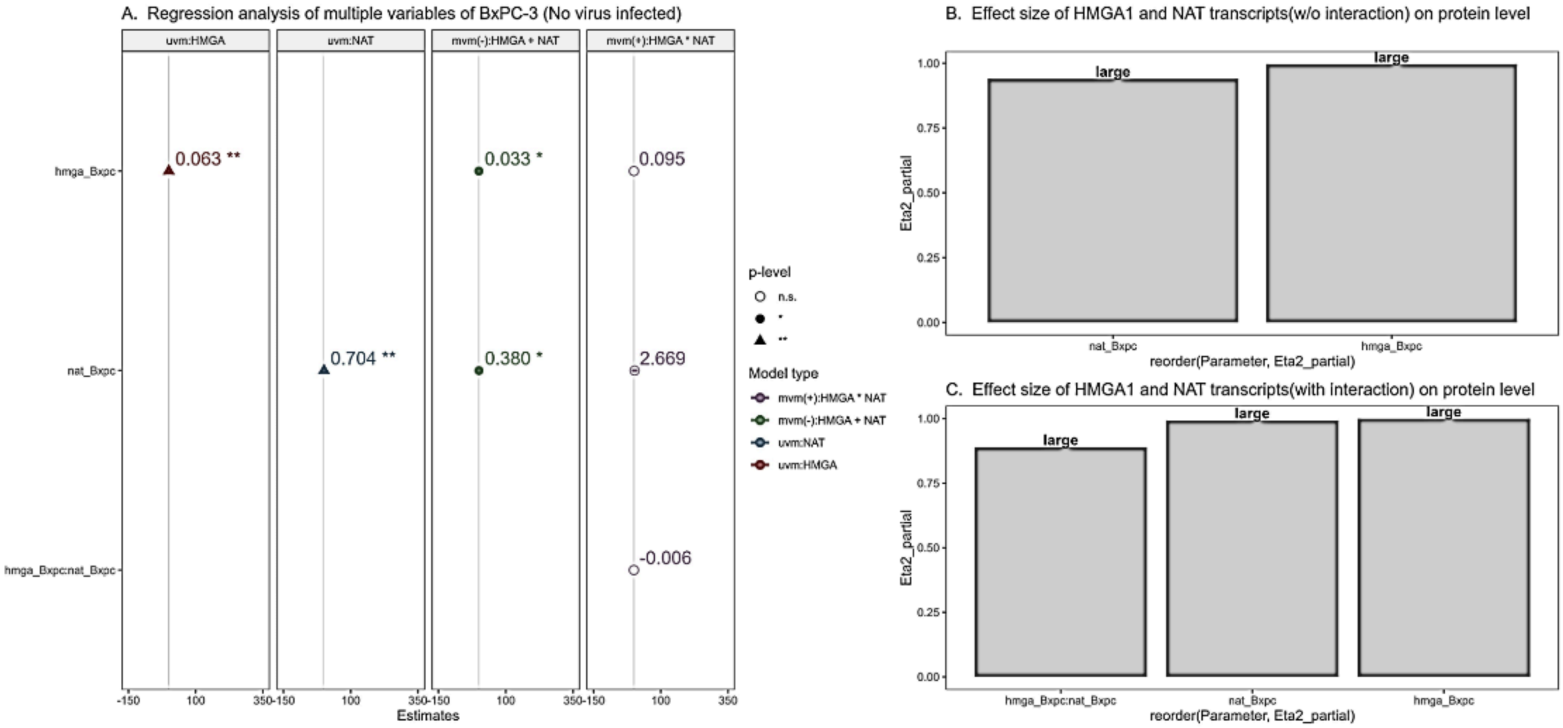

S Fig. 32: Regression analysis and effect size calculation of BxPC-3 cells (No infection). According to AIC values, with interaction model works best for this cell line. Effect of HMGA1 and NAT transcripts and their interactions showed almost equal impact on maintaining high protein level (C).

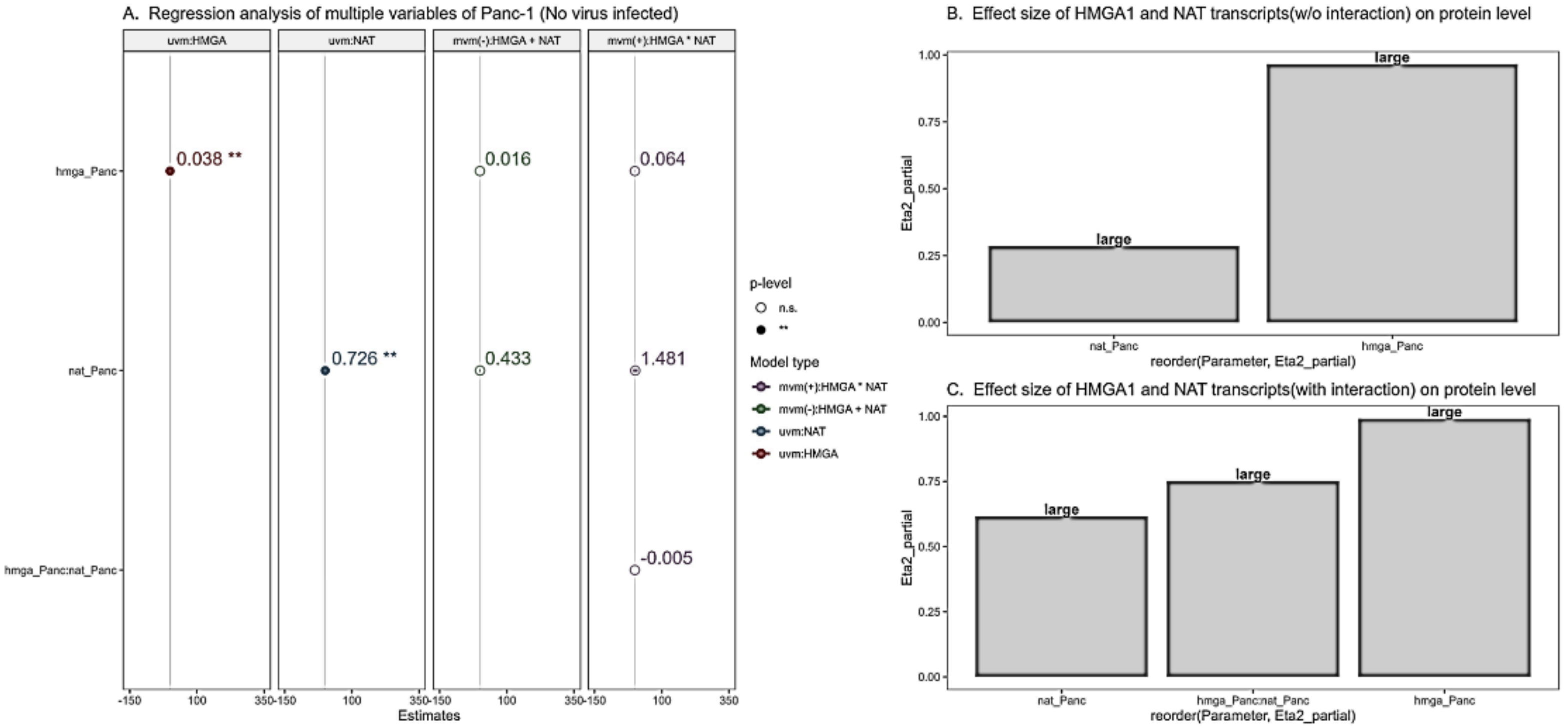

S Fig. 33: Regression analysis and effect size calculation of PANC-1 cells (No infection). According to AIC values, with interaction model works best for this cell line. Effect of HMGA1 and NAT transcripts and their interactions showed different impact on maintaining high protein level (C).

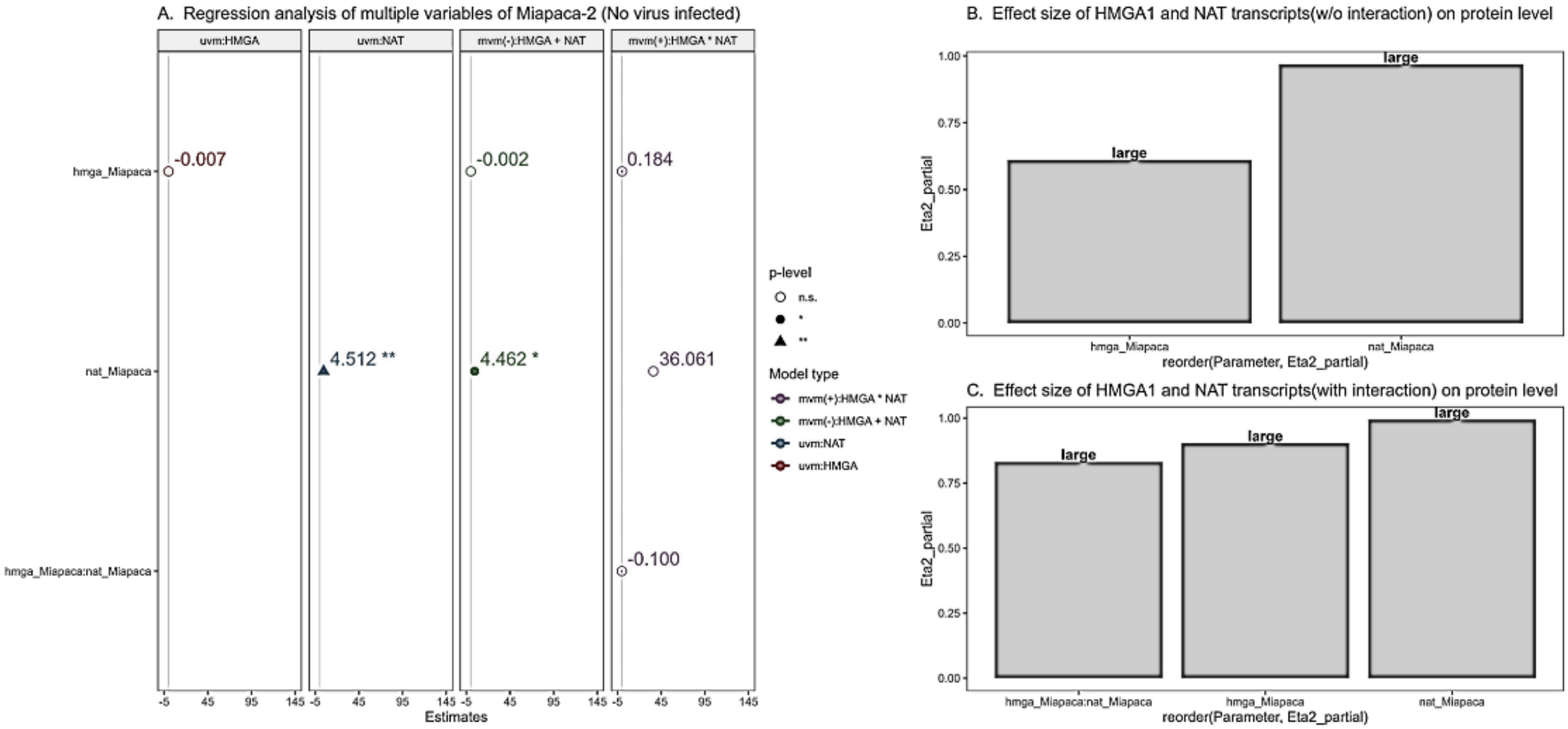

S Fig. 34: Regression analysis and effect size calculation of MiaPaCa-2 cells (No infection). According to AIC values, with interaction model works best for this cell line. Effect of HMGA1 and NAT transcripts and their interactions showed lower impact on maintaining high protein level (C).

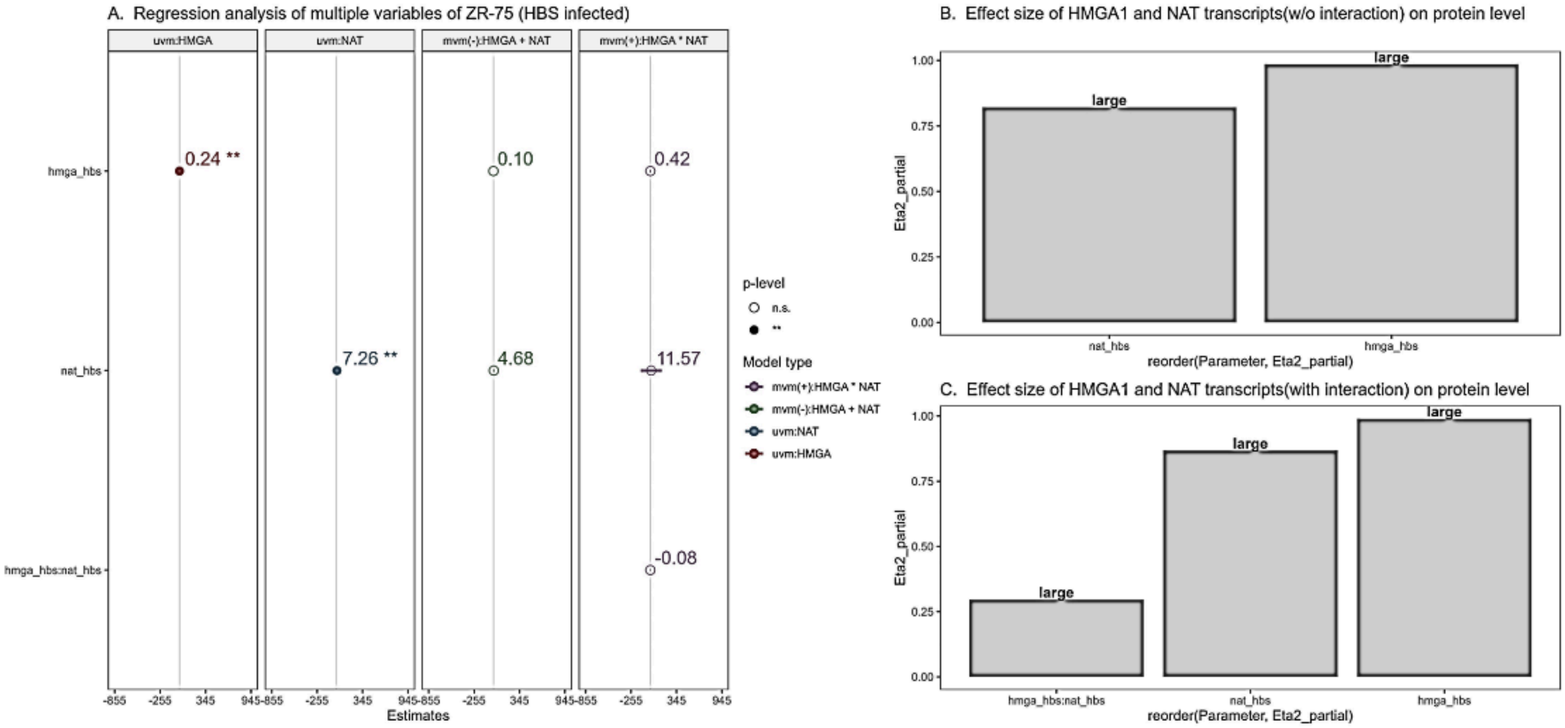

S Fig. 35: Regression analysis and effect size calculation of ZR-75 (HBS infected). According to AIC values, without interaction model works best for this cell line. Effects of NAT transcripts showed higher impact than ZR-75 cells (no infection) on reducing protein level (B).

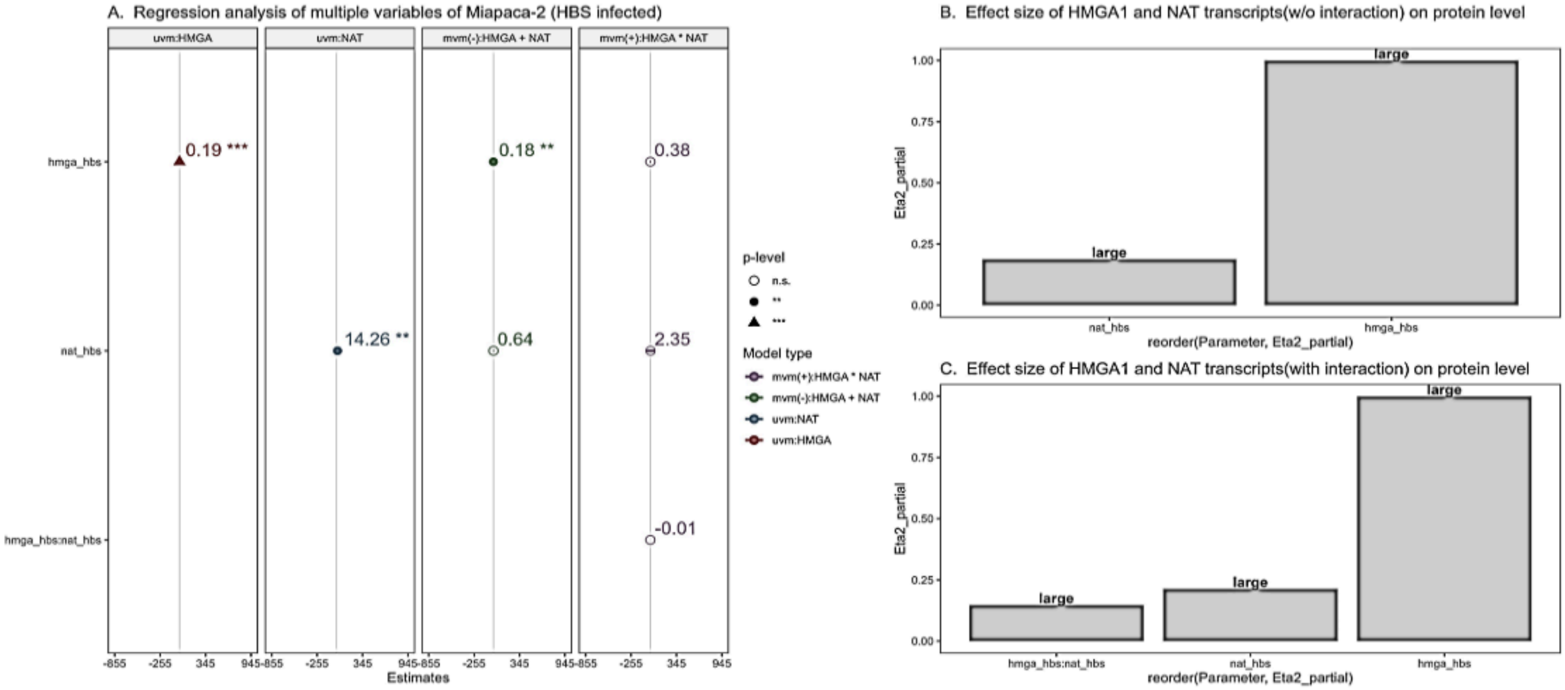

S Fig. 36: Regression analysis and effect size calculation of MiaPaCa-2 cells (HBS infected). According to AIC values, without interaction model seems better but due to VIF value interaction exists. Effect of NAT transcripts and their interactions showed very low impact on reduced protein level (C).

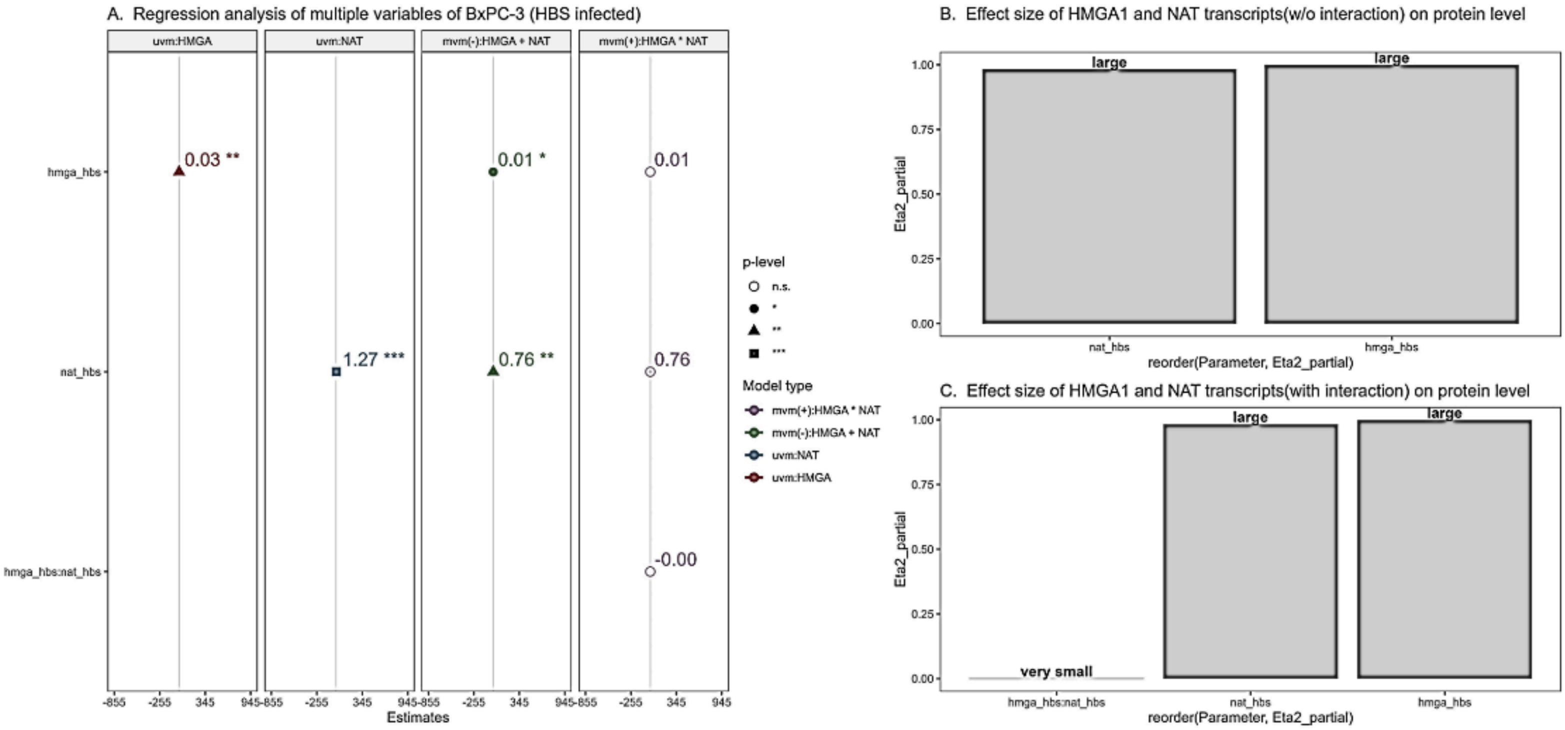

S Fig. 37: Regression analysis and effect size calculation of BxPC-3 cells (HBS infected). According to AIC values, without interaction model seems better but due to VIF value interaction exists. Effect of HMGA1 and NAT transcripts showed equal impact on maintaining reduced protein level (C).

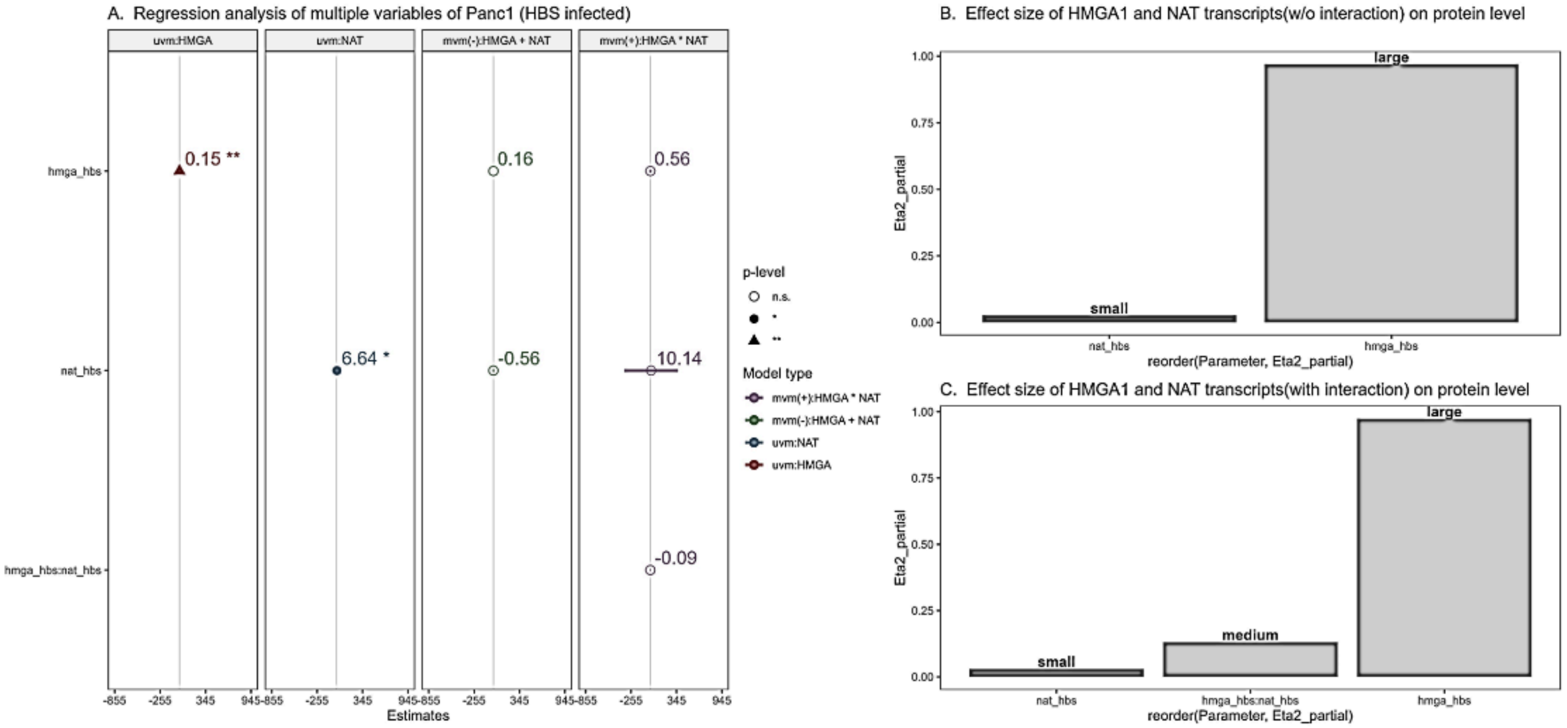

S Fig. 38: Regression analysis and effect size calculation of PANC-1 cells (HBS infected). According to AIC values, without interaction model works best for this cell line. Effect of HMGA1 showed impact on reduced protein level (B).

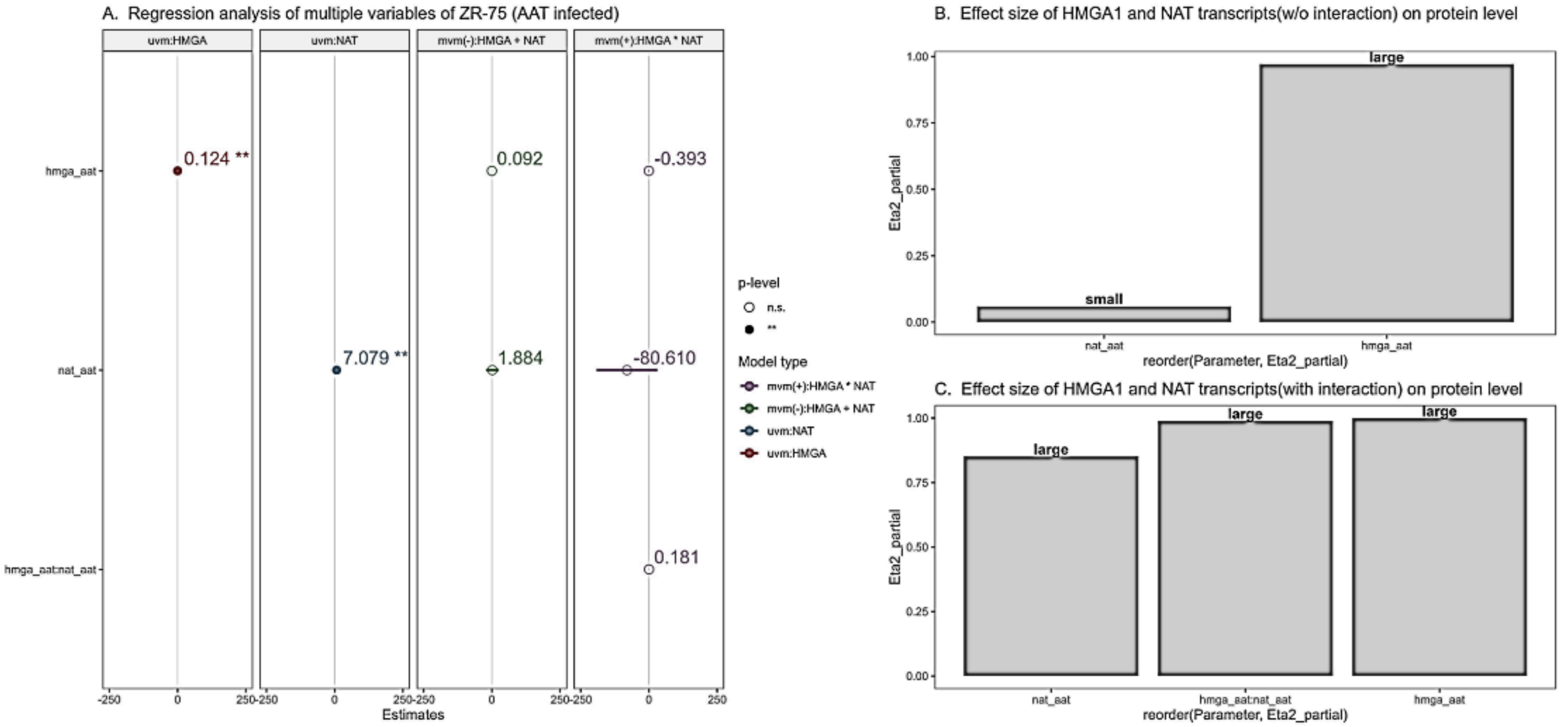

S Fig. 39: Regression analysis and effect size calculation of ZR-75 (AAT infected). According to AIC values, with interaction model works best for this cell line. Effect of HMGA1 and NAT transcripts and their interactions showed almost equal impact on reducing protein level (C).

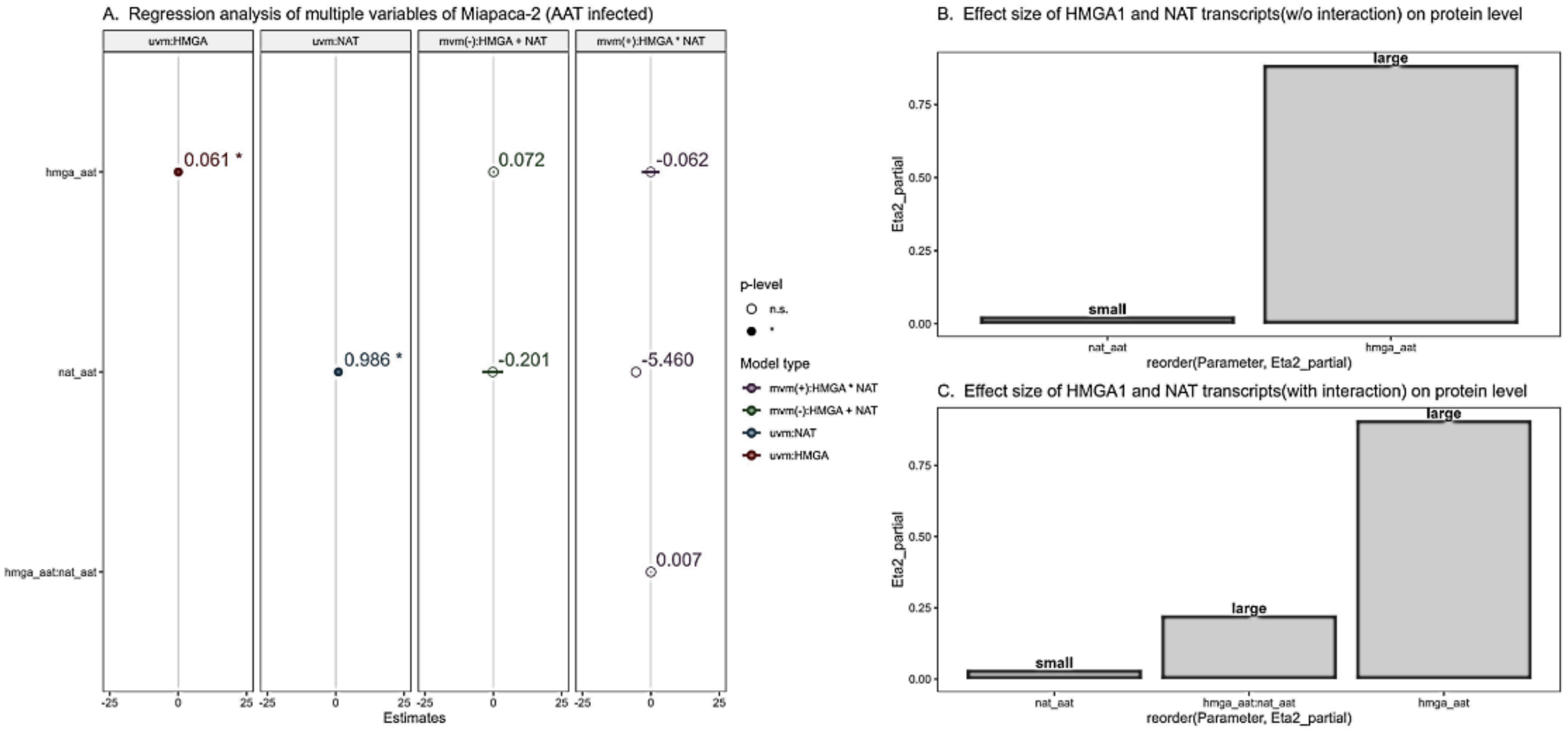

S Fig. 40: Regression analysis and effect size calculation of MiaPaCa-2 cells (AAT infected). According to AIC values, without interaction model seems better but due to VIF value interaction exists. Effect of NAT transcripts and their interactions showed very lower impact on reduced protein level (C).

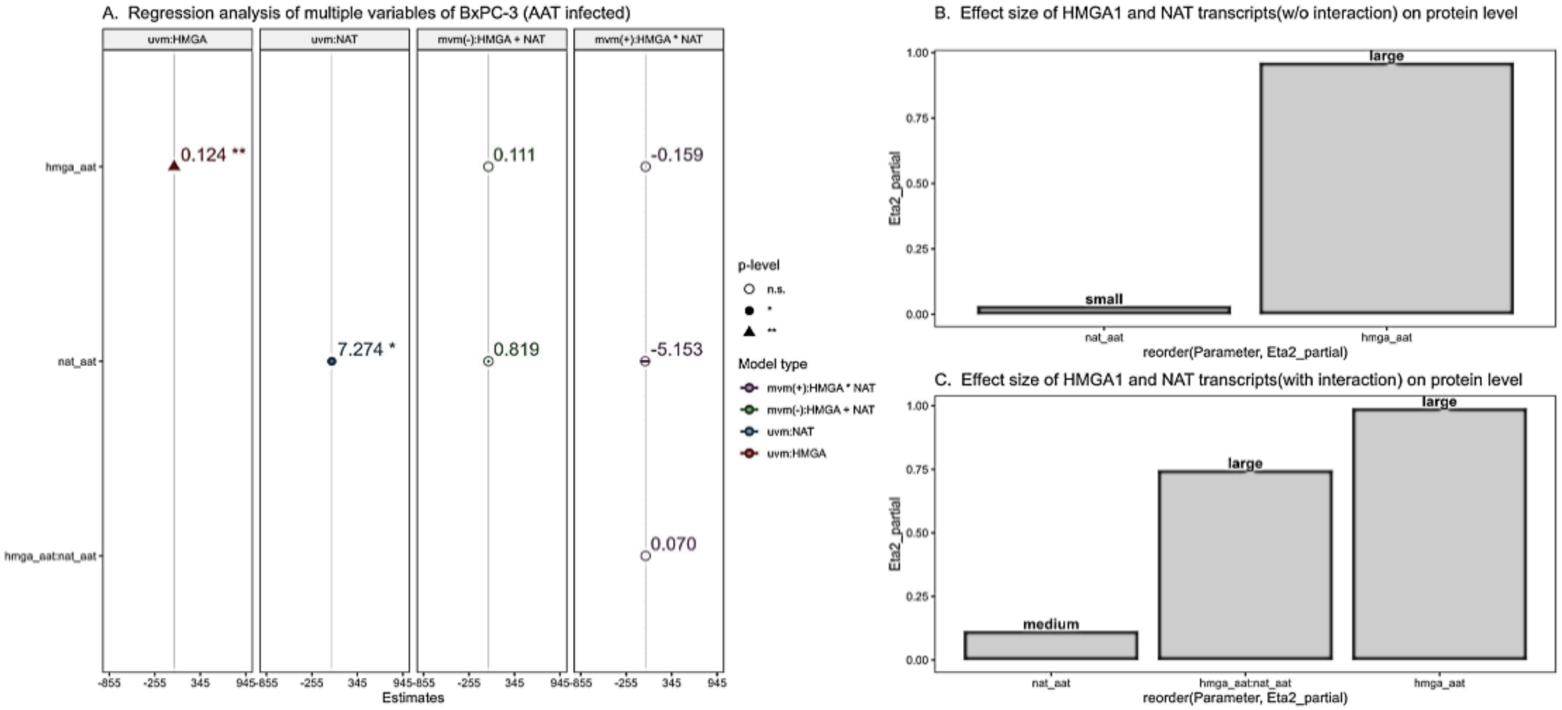

S Fig. 41: Regression analysis and effect size calculation of BxPC-3 cells (AAT infected). According to AIC values, with interaction model works best for this cell line. Effect of HMGA1 and NAT transcripts and their interactions showed different impact on reduced protein level (C).

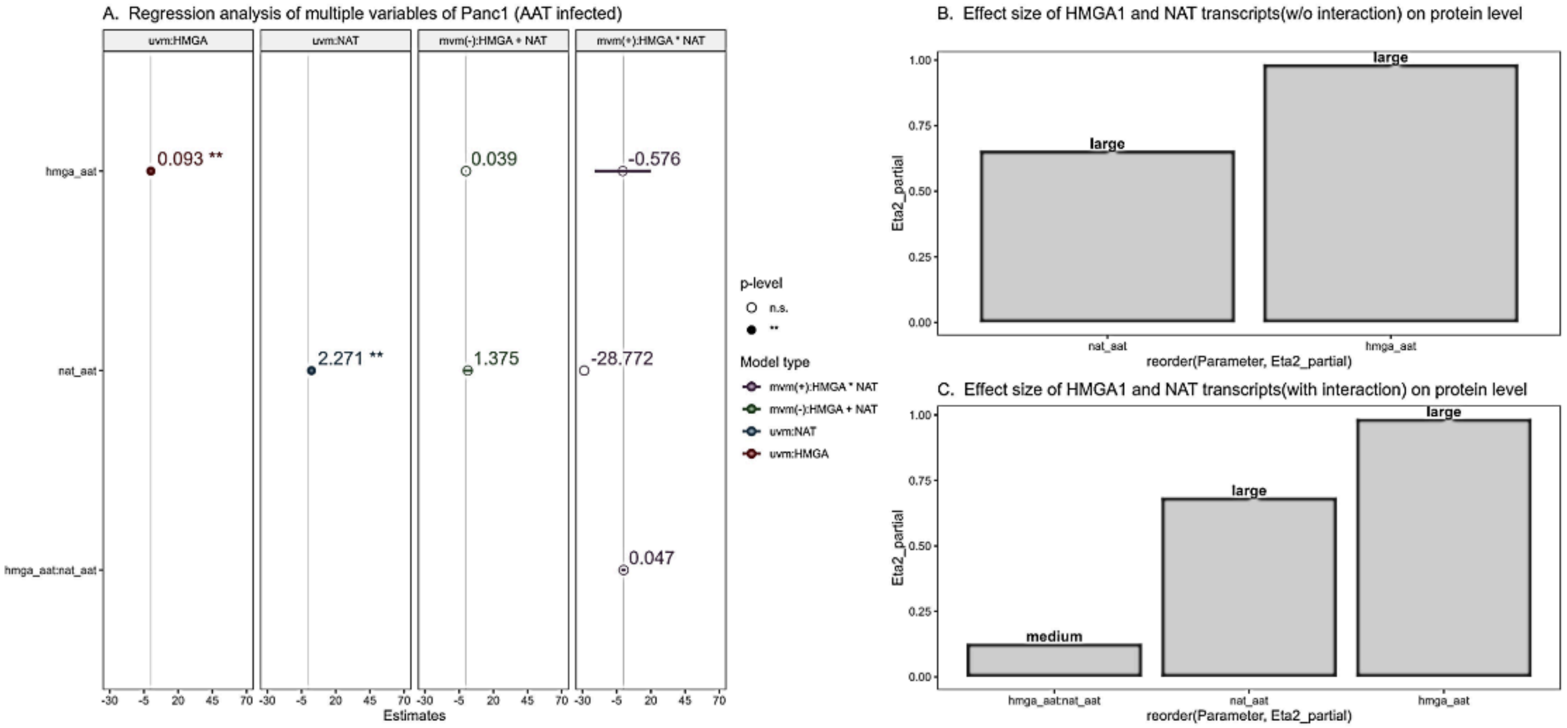

S Fig. 42: Regression analysis and effect size calculation of PANC-1 cells (AAT infected). According to AIC values, without interaction model seems better but due to VIF value interaction exists. Effect of HMGA1 and NAT transcripts and their interactions showed different impact on reduced protein level (C).

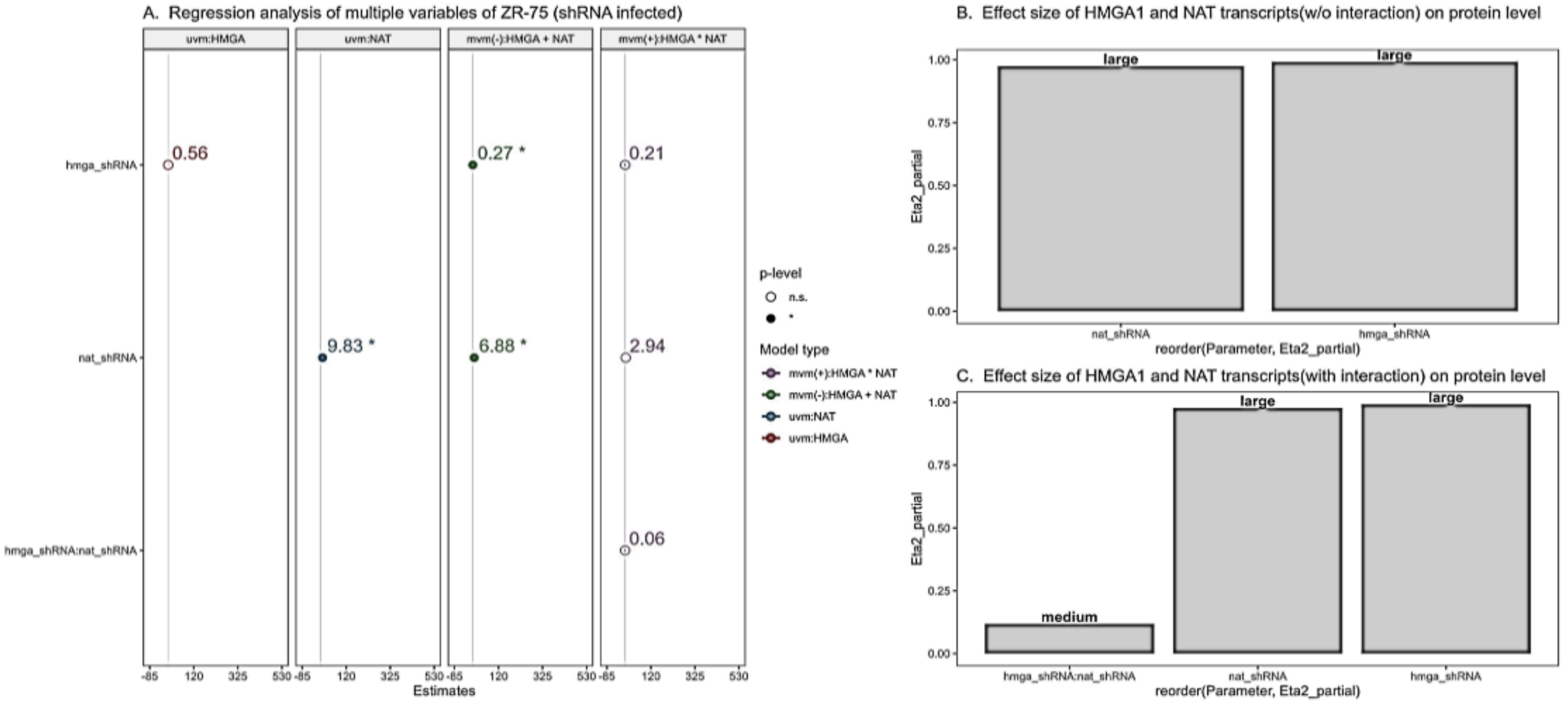

S Fig. 43: Regression analysis and effect size calculation of ZR-75 (shRNA infected). According to AIC values, without interaction model works best for this cell line. Effect of HMGA1 and NAT transcripts showed equal impact on reducing protein level (C).

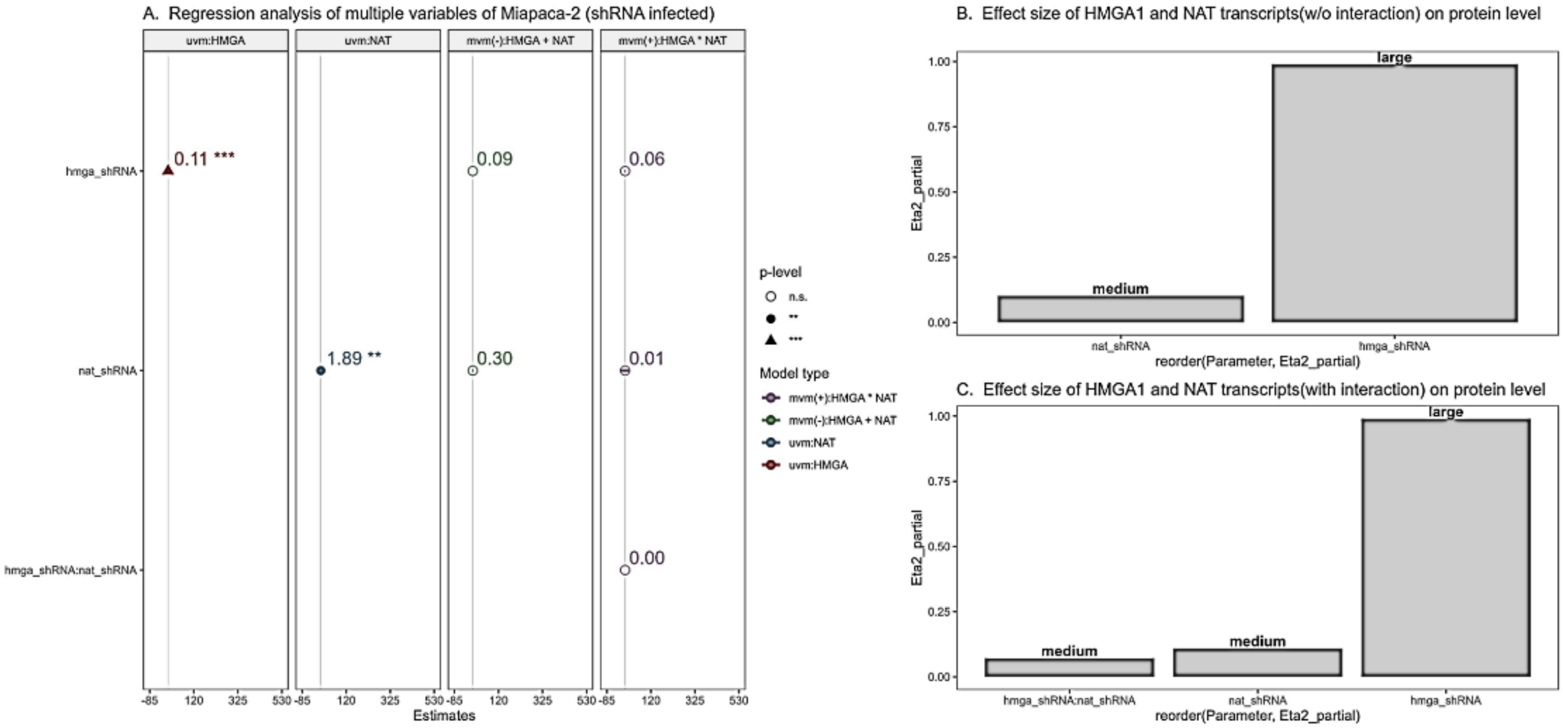

S Fig. 44: Regression analysis and effect size calculation of MiaPaCa-2 cells (shRNA infected). According to AIC values, without interaction model seems better but due to VIF value interaction exists. Effect of HMGA1 and NAT transcripts and their interactions showed lower impact on reduced protein level (C).

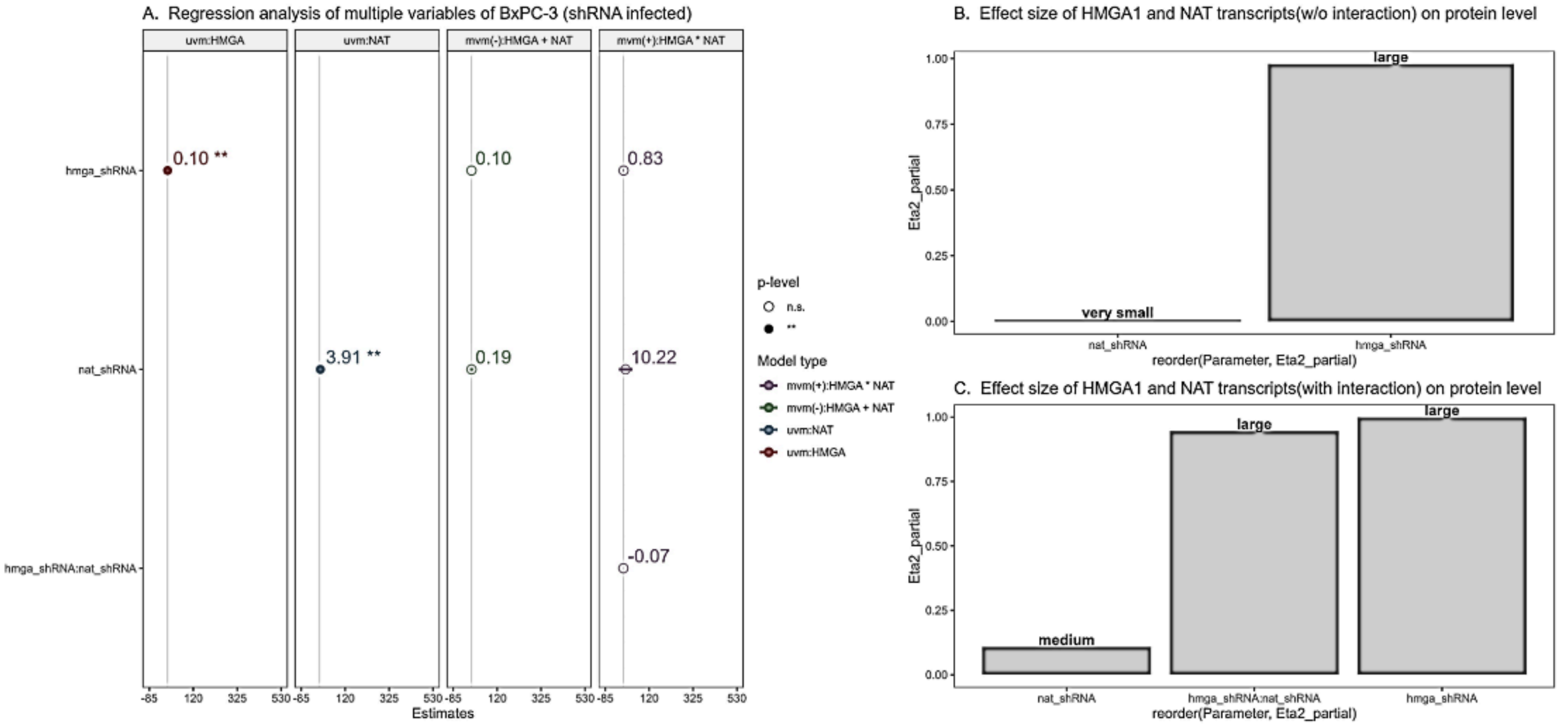

S Fig. 45: Regression analysis and effect size calculation of BxPC-3 cells (shRNA infected). According to AIC values, with interaction model works best for this cell line. Effect of HMGA1 and NAT transcripts and their interactions showed impact on reduced protein level (C).

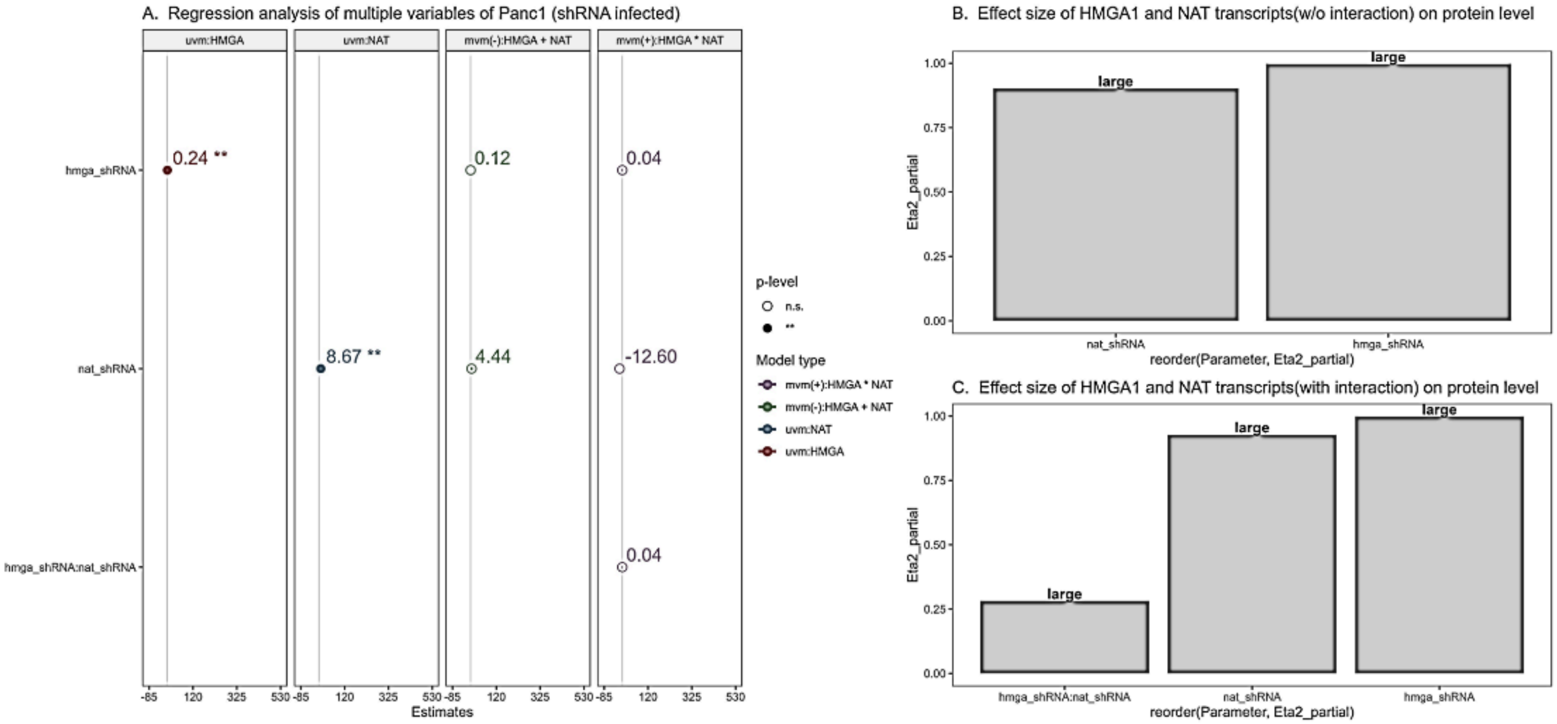

S Fig. 46: Regression analysis and effect size calculation of PANC-1 cells (shRNA infected). According to AIC values, without interaction model seems better but due to VIF value interaction exists. Effect of HMGA1 and NAT transcripts and their interactions showed different impact on reduced protein level (C).

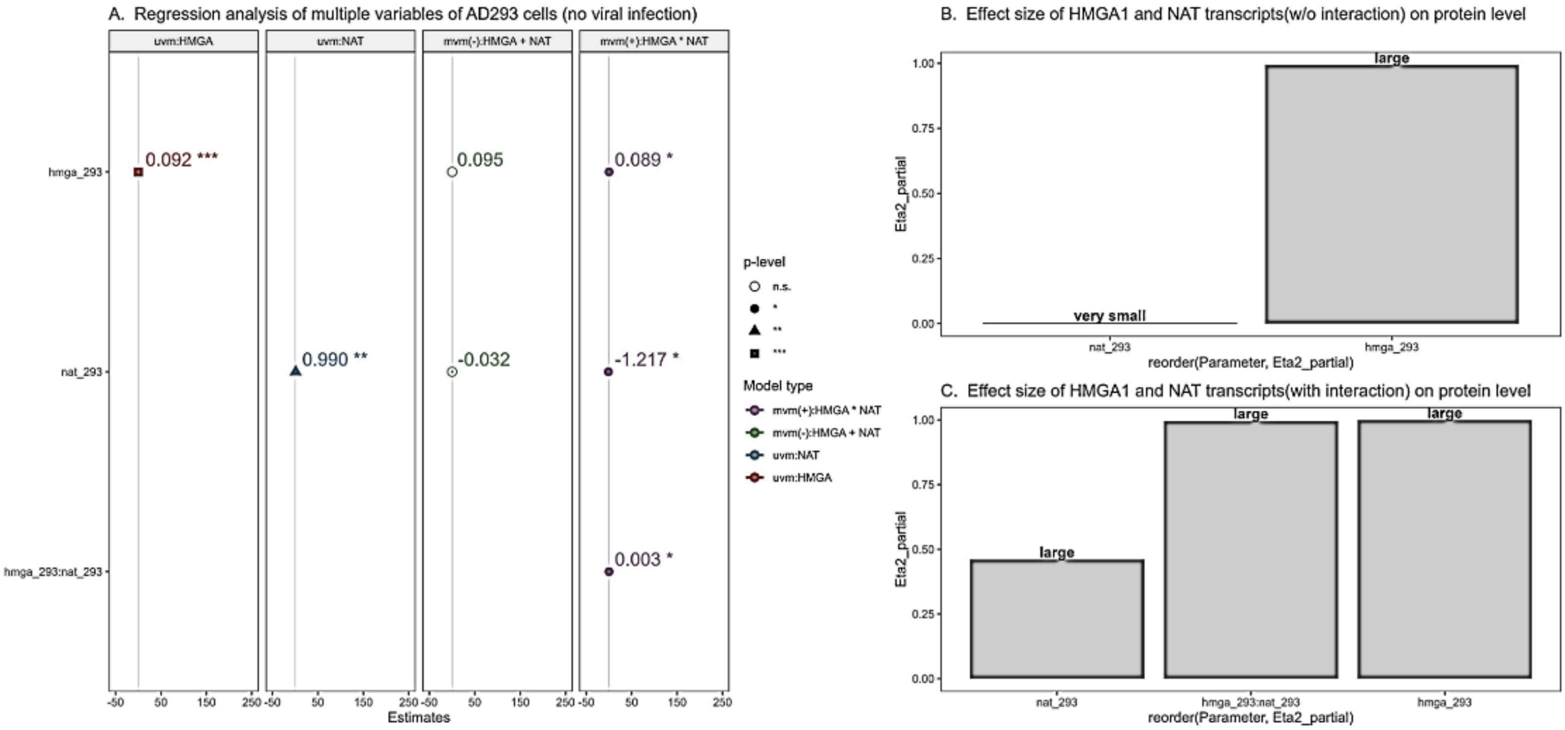

S Fig. 47: Regression analysis and effect size calculation of AD293 cells (not infected). According to AIC values, with interaction model works best for this cell line. Effect of HMGA1 and NAT transcripts and their interactions showed different impact on higher protein level (C).

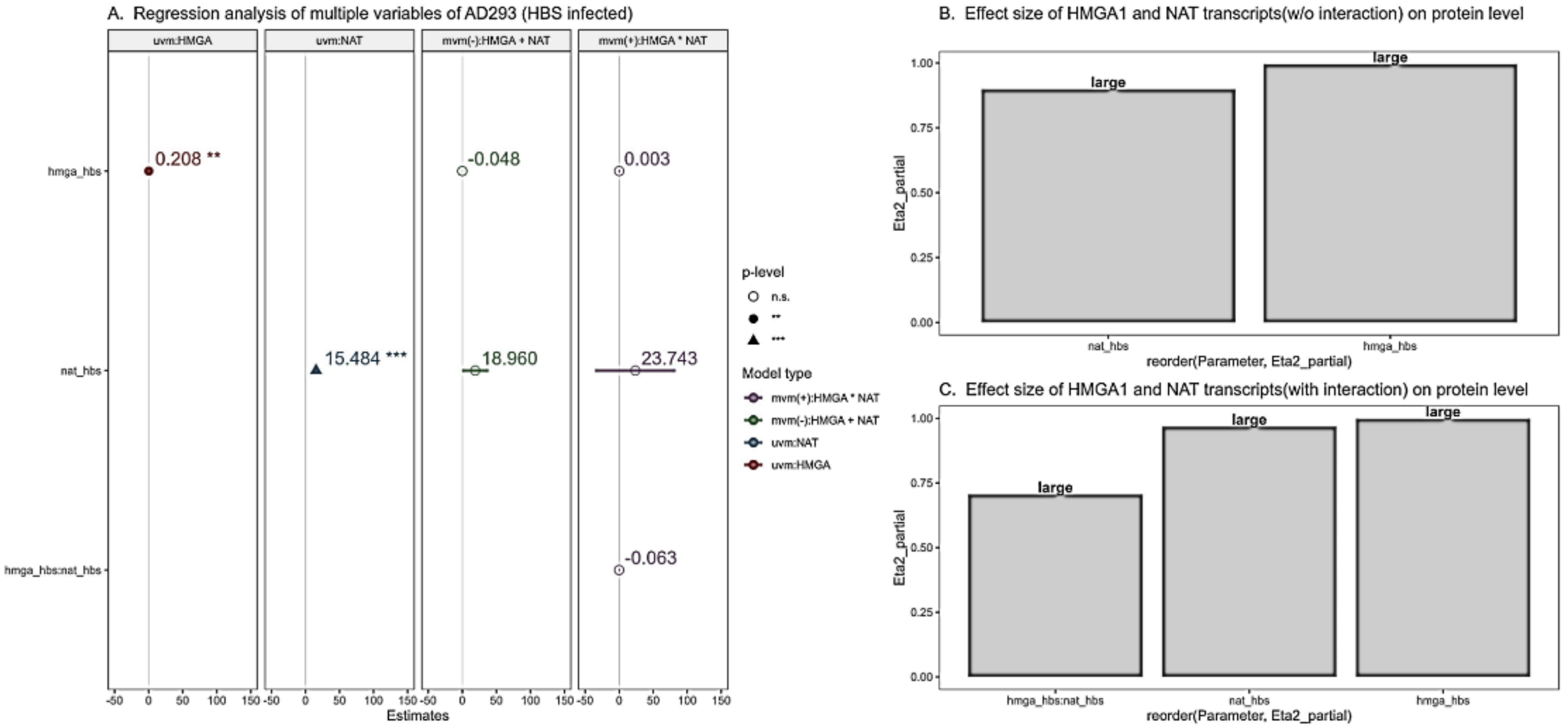

S Fig. 48: Regression analysis and effect size calculation of AD293 cells (HBS infected). According to AIC values, with interaction model works best for this cell line. Effect of HMGA1 and NAT transcripts and their interactions showed higher impact on reduced protein level (C).

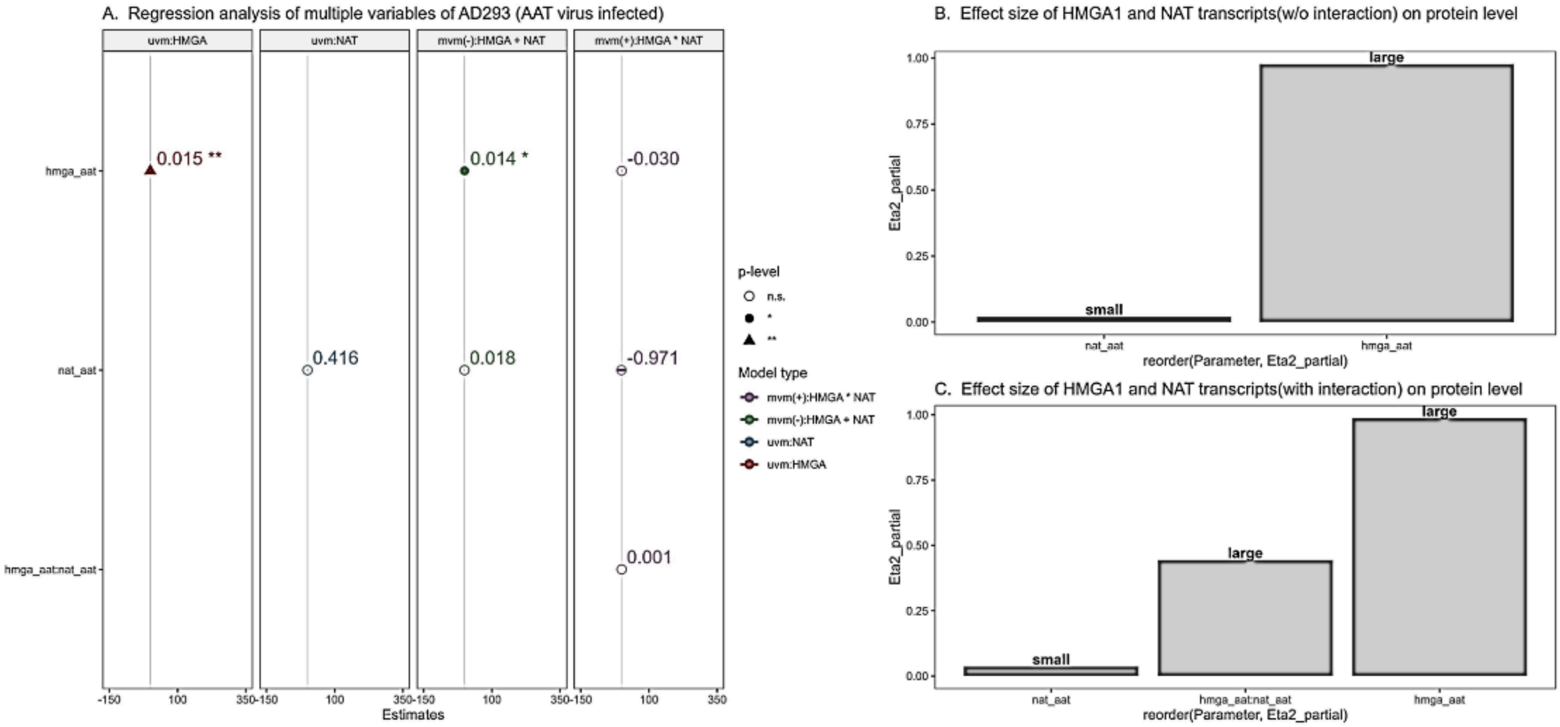

S Fig. 49: Regression analysis and effect size calculation of AD293 cells (AAT infected). According to AIC values, with interaction model works best for this cell line. Effect of HMGA1 and NAT transcripts and their interactions showed lower impact on reduced protein level (C).

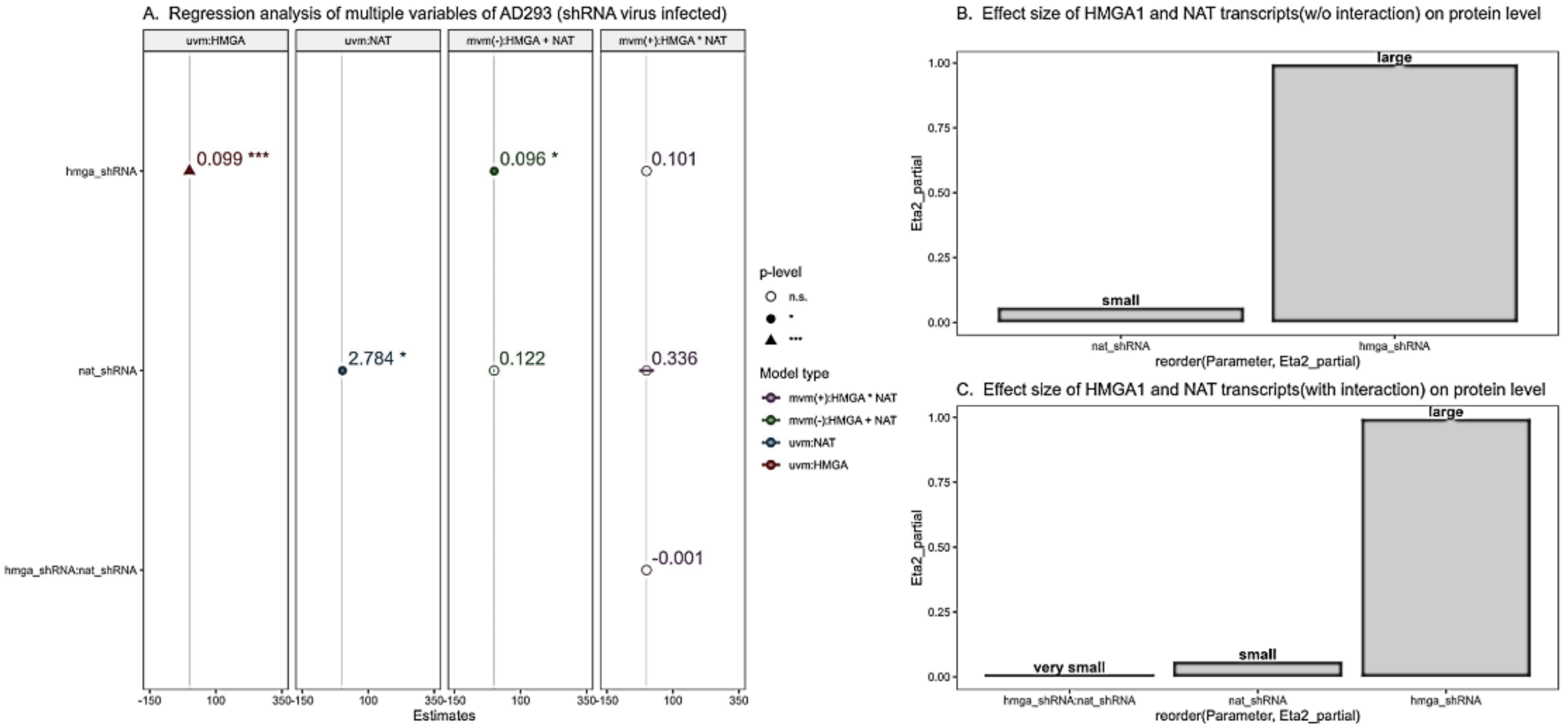

S Fig. 50: Regression analysis and effect size calculation of AD293 cells (shRNA infected). According to AIC values, without interaction model works best for this cell line. Effect of NAT transcripts showed lower impact on reduced protein level (B).

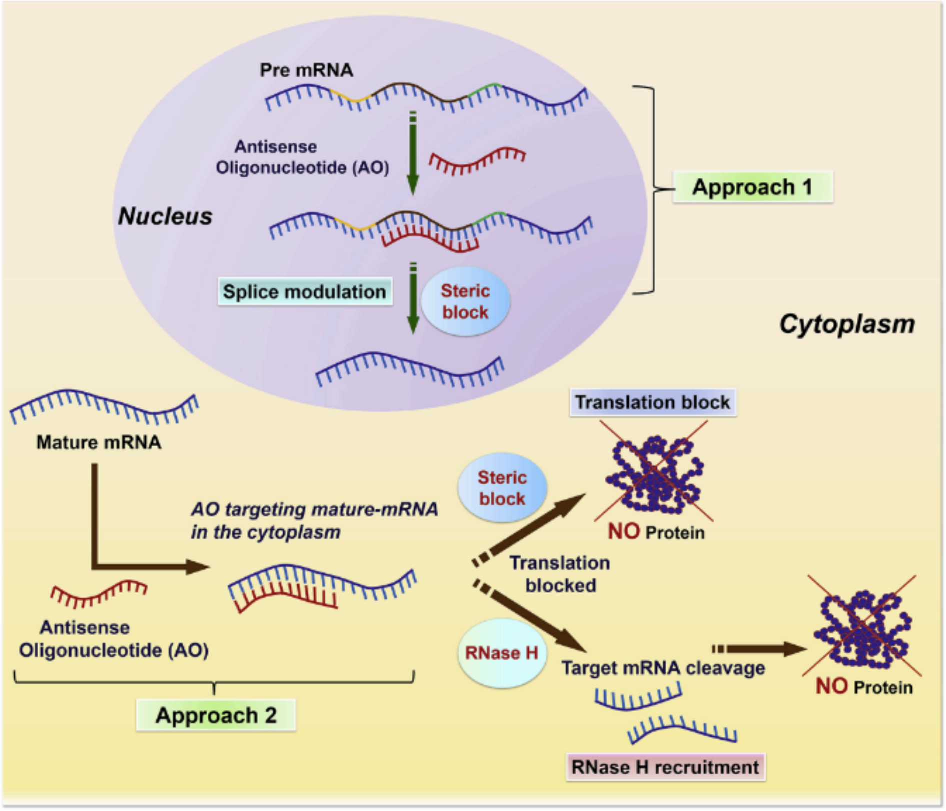

S Fig. 51: Different types of antisense mechanisms that could lead to target mRNA degradation. One of them works within nucleus (by altering splicing) and the other two in the cytoplasm (one is Rnase-H pathway, another is to create steric blocking to stop translation).

Image source: https://www.cell.com/molecular-therapy-family/nucleic-acids/fulltext/S2162-2531(18)30301-9

Supplementary Table 1: List of primer pairs designed for NAT detection.

Target position	Forward primer	Reverse primer
2	AGGCTCTCTGGGATTCCGG	AATACGGTGGGACTCCCATGTCTA
3.1	CCACTGAACTGCGTAGGCTGGAT	GGCTGGACTTCGAGCTCG
3.2	TCCAAGCAGGAAAAGGACGG	GGAAGTACAAGAATCGACCATAAGTATTCAGC
4.1	TGTCCAGCATAGGTCATCTGAGAGG	AGGTGTTGGCACTTCGCT
4.2	GACTTAACCTTGTTGAGGAGGCA	TGTGAATCTGGCCTTTGCTGCC
5.1	GGCGATGACTAAGGTCTTACTTCTGG	TCCTTCCTGGAGTTGTGGTG
5.2	TCCTTCCTGGAGTTGTGGTG	TGTGGTGGGTCACATCAGAACTC
6	ACCAGCGGGCTCTTGCAC	AGAAGGAAGCTGCTCCTCCAGT

Supplementary Table 2: qPCR primer pairs and probes designed for NAT quantification.

Target	Forward primer	Reverse primer	Probe
HMGA1	GCCCTCCTCTTCCTCCTT	GAAGTGCCAACACCT AAGAG	/56- FAM/AGACCCGGA/ZEN/AAACCACCACAACT/ 3IABkFQ/
NAT 3.1	TTCTGCAGTGTGCCTTG AA	GGCTGGACTTCGAGC TC	/5Cy5/TCTCCCAGC/TAO/ATCCCAGCCATC/3I AbRQSp/
NAT 4.1	CTTAACCTTGTTGAGGA GGCA	AGGTGTTGGCACTTC GC	/56- TexRed/TCTTTACAG/ZEN/AAGGAGCCCAGC GAAG/3IABkFQ/
NAT 5.1	CTGAACAGGCAGGTAG GTC	TCTGCTTCTTCACCTA CCAG	/5HEX/ACCCTCTAG/ZEN/AAAACCACCACAA CTCC/3IABkFQ/
AAT	AGCTCGGATCCACTAGT AACGG	ACCACCACAACTCCA GG	/56- FAM/TGTGCTGGA/ZEN/ATTCTCACTGCTCCT CCTCCG/3IABkFQ/

Supplementary Table 3-9: Linear Regression analysis of one independent variable (either HMGA1 or NAT transcript).

Left and right column indicate "HMGA1" and "NAT" independent variable, respectively. In linear regression, the effect of NAT is assumed constant while analyzing relation between HMGA1 transcript and protein level. Similarly, HMGA1 remains constant during NAT vs protein level analysis. Green box provides AIC values. Lowest AIC value indicates the best fitted model. In these models, p values are lower than 0.05, indicating statistical significance. These models do not consider any other variables/factors present in the biological system.

S Table 3: Regression analysis of breast epithelial and ZR-75 (not infected) cells. Yellow box shows p-value which indicate significant differences.

	UVM: HMGA			UVM: NAT		
**Predictor**	**Beta**[1]	**95% CI**[2]	**p-value**	**Beta**[1]	**95% CI**[2]	**p-value**
Breast Epithelial cells						
**hmga_br_epi**	0.50**	0.25, 0.75	**0.008**			
R^2	0.931			0.843		
Adjusted R^2	0.908			0.791		
AIC	6.33			10.4		
**nat_br_epi**				16*	3.4, 29	**0.028**
ZR-75 breast cancer cells(no virus infected)						
**hmga_ZR**	0.12**	0.07, 0.18	**0.006**			
R^2	0.944			0.935		
Adjusted R^2	0.925			0.914		
AIC	9.87			10.6		
**nat_ZR**				4.6**	2.4, 6.8	**0.007**

[1] *p<0.05; **p<0.01; ***p<0.001

[2] CI = Confidence Interval

Supplementary Table 4: Regression analysis of ZR-75 cells (virus infected)

	UVM: HMGA			UVM: NAT		
**Predictor**	**Beta**[1]	**95% CI**[2]	**p-value**	**Beta**[1]	**95% CI**[2]	**p-value**
HBS virus infected						
**hmga_hbs**	0.24**	0.12, 0.37	**0.009**			
R^2	0.925			0.964		
Adjusted R^2	0.900			0.953		
AIC	21.4			17.7		
**nat_hbs**				7.3**	4.7, 9.8	**0.003**
AAT virus infected						
**hmga_aat**	0.12**	0.08, 0.16	**0.002**			
R^2	0.969			0.957		
Adjusted R^2	0.959			0.943		
AIC	17.4			19.1		
**nat_aat**				7.1**	4.3, 9.8	**0.004**
shRNA virus infected						
**hmga_shRNA**	0.56	-0.03, 1.2	0.057			
R^2	0.752			0.895		
Adjusted R^2	0.669			0.860		
AIC	30.6			26.3		
**nat_shRNA**				9.8*	3.6, 16	**0.015**

[1] *p<0.05; **p<0.01; ***p<0.001

[2] CI = Confidence Interval

Supplementary Table 5: Regression analysis of pancreatic epithelial cells and cancer cells (not infected)

	UVM: HMGA			UVM: NAT		
**Predictor**	**Beta**[1]	**95% CI**[2]	**p-value**	**Beta**[1]	**95% CI**[2]	**p-value**
Pancreatic Epithelial cells						
**hmga_pan_epi**	1.0	-1.3, 3.3	0.3			
R^2	0.390			0.776		
Adjusted R^2	0.186			0.701		
AIC	20.9			15.9		
**nat_pan_epi**				32*	0.38, 64	**0.049**
Miapaca-2 cancer cells(no virus infected)						
**hmga_Miapaca**	-0.01	-0.06, 0.05	0.7			
R^2	0.047			0.965		
Adjusted R^2	-0.271			0.953		
AIC	18.3			1.82		
**nat_Miapaca**				4.5**	2.9, 6.1	**0.003**
BxPC-3 cancer cells(no virus infected)						
**hmga_Bxpc**	0.06**	0.03, 0.09	**0.007**			
R^2	0.938			0.942		
Adjusted R^2	0.917			0.923		
AIC	6.75			6.41		
**nat_Bxpc**				0.70**	0.38, 1.0	**0.006**
Panc-1 cancer cells(no virus infected)						
**hmga_Panc**	0.04**	0.02, 0.05	**0.005**			
R^2	0.952			0.959		
Adjusted R^2	0.936			0.945		
AIC	6.03			5.21		
**nat_Panc**				0.73**	0.45, 1.0	**0.004**

[1] *p<0.05; **p<0.01; ***p<0.001

[2] CI = Confidence Interval

Supplementary Table 6: Regression analysis of MiaPaCa-2 cells (virus infected)

	UVM: HMGA			UVM: NAT		
**Predictor**	**Beta**[1]	**95% CI**[2]	**p-value**	**Beta**[1]	**95% CI**[2]	**p-value**
HBS virus infected						
**hmga_hbs**	0.19***	0.18, 0.20	**<0.001**			
R^2	1.00			0.960		
Adjusted R^2	0.999			0.947		
AIC	-1.85			20.6		
**nat_hbs**				14**	8.9, 20	**0.003**
AAT virus infected						
**hmga_aat**	0.06*	0.02, 0.10	**0.018**			
R^2	0.883			0.772		
Adjusted R^2	0.844			0.696		
AIC	23.1			26.5		
**nat_aat**				0.99*	0.00, 2.0	**0.050**
shRNA virus infected						
**hmga_shRNA**	0.11***	0.08, 0.13	**<0.001**			
R^2	0.988			0.953		
Adjusted R^2	0.984			0.938		
AIC	15.8			22.7		
**nat_shRNA**				1.9**	1.1, 2.7	**0.004**

[1] *p<0.05; **p<0.01; ***p<0.001

[2] CI = Confidence Interval

Supplementary Table 7: Regression analysis of BxPC-3 cells (virus infected)

	UVM: HMGA			UVM: NAT		
**Predictor**	**Beta**[1]	**95% CI**[2]	**p-value**	**Beta**[1]	**95% CI**[2]	**p-value**
HBS virus infected						
**hmga_hbs**	0.03**	0.02, 0.04	**0.002**			
R^2	0.969			0.984		
Adjusted R^2	0.959			0.979		
AIC	16.3			13.0		
**nat_hbs**				1.3***	0.97, 1.6	**<0.001**
AAT virus infected						
**hmga_aat**	0.12**	0.08, 0.17	**0.003**			
R^2	0.961			0.877		
Adjusted R^2	0.948			0.836		
AIC	14.5			20.3		
**nat_aat**				7.3*	2.3, 12	**0.019**
shRNA virus infected						
**hmga_shRNA**	0.10**	0.08, 0.13	**0.001**			
R^2	0.979			0.922		
Adjusted R^2	0.972			0.896		
AIC	12.4			18.9		
**nat_shRNA**				3.9**	1.8, 6.0	**0.009**

[1] *p<0.05; **p<0.01; ***p<0.001

[2] CI = Confidence Interval

Supplementary Table 8: Regression analysis of PANC-1 cells (virus infected)

	UVM: HMGA			UVM: NAT		
**Predictor**	**Beta**[1]	**95% CI**[2]	**p-value**	**Beta**[1]	**95% CI**[2]	**p-value**
HBS virus infected						
**hmga_hbs**	0.15**	0.10, 0.20	**0.002**			
R^2	0.969			0.817		
Adjusted R^2	0.959			0.756		
AIC	17.7			26.6		
**nat_hbs**				6.6*	0.86, 12	**0.035**
AAT virus infected						
**hmga_aat**	0.09**	0.06, 0.13	**0.004**			
R^2	0.955			0.971		
Adjusted R^2	0.940			0.961		
AIC	17.0			14.8		
**nat_aat**				2.3**	1.5, 3.0	**0.002**
shRNA virus infected						
**hmga_shRNA**	0.24**	0.17, 0.32	**0.002**			
R^2	0.973			0.973		
Adjusted R^2	0.964			0.963		
AIC	22.0			22.0		
**nat_shRNA**				8.7**	6.0, 11	**0.002**

[1] *p<0.05; **p<0.01; ***p<0.001

[2] CI = Confidence Interval

Supplementary Table 9: Regression analysis of AD-293 cells (virus infected)

	UVM: HMGA			UVM: NAT		
**Predictor**	**Beta**[1]	**95% CI**[2]	**p-value**	**Beta**[1]	**95% CI**[2]	**p-value**
AD293 cells(no viral infection)						
**hmga_293**	0.09***	0.08, 0.11	**<0.001**			
R^2	0.994			0.973		
Adjusted R^2	0.991			0.964		
AIC	4.20			11.3		
**nat_293**				0.99**	0.69, 1.3	**0.002**
AD293 cells(HBS virus infected)						
**hmga_hbs**	0.21**	0.12, 0.29	**0.005**			
R^2	0.952			0.994		
Adjusted R^2	0.936			0.991		
AIC	18.6			8.45		
**nat_hbs**				15***	13, 18	**<0.001**
AD293 cells(AAT virus infected)						
**hmga_aat**	0.01**	0.01, 0.02	**0.002**			
R^2	0.976			0.467		
Adjusted R^2	0.968			0.290		
AIC	16.3			31.8		
**nat_aat**				0.42	-0.40, 1.2	0.2
AD293 cells(shRNA virus infected)						
**hmga_shRNA**	0.10***	0.09, 0.11	**<0.001**			
R^2	0.995			0.819		
Adjusted R^2	0.993			0.758		
AIC	9.48			27.3		
**nat_shRNA**				2.8*	0.38, 5.2	**0.035**

[1] *p<0.05; **p<0.01; ***p<0.001

[2] CI = Confidence Interval

Supplementary Table 10-17: Multilinear Regression analysis of multiple variables in different cells.

Left and right column indicate "no interaction" and "with interaction" models respectively. Green and red box provide AIC and VIF values respectively. Lowest AIC value indicates the best fitted model than another when VIF value is less than 10. If VIF value is more than 10 then "with interaction" is the best fitted model. In these multilinear models, p values are much higher than 0.05, indicating no statistical significance. However, in liner models (S Table 3-9), most of the variables showed significant p-values (<0.05) while analyzed as individual independent variable.

	MVM(-): HMGA + NAT				MVM(+): HMGA * NAT			
**Predictor**	**Beta**[1]	**95% CI**[2]	**p-value**	**VIF**[2]	**Beta**[1]	**95% CI**[2]	**p-value**	**VIF**[2]
Breast Epithelial cells								
**hmga_br_epi**	0.34	-0.19, 0.88	0.11	3.5	1.1	-1.0, 3.3	0.094	77
**nat_br_epi**	6.3	-12, 25	0.3	3.5	51	-69, 170	0.12	205
R^2	0.967				0.999			
Adjusted R^2	0.935				0.994			
AIC	4.59				-9.25			
**hmga_br_epi * nat_br_epi**					-5.6	-21, 9.4	0.13	481
ZR-75 breast cancer cells(no virus infected)								
**nat_ZR**	1.9	-1.9, 5.6	0.2	13	4.8	-78, 87	0.6	430
**hmga_ZR**	0.05	-0.11, 0.21	0.3	13	0.05	-0.57, 0.68	0.5	14
R^2	0.987				0.989			
Adjusted R^2	0.973				0.956			
AIC	4.55				5.57			
**hmga_ZR * nat_ZR**					-0.03	-0.95, 0.88	0.7	459

[1] *p<0.05; **p<0.01; ***p<0.001

[2] CI = Confidence Interval, VIF = Variance Inflation Factor

Supplementary Table 10: Regression analysis of multiple variables in breast epithelial and ZR-75 cells (not infected). Yellow box shows p-value which indicate no significant differences. While compared with S Table 3, we found that the significant effects of HMGA1 and NAT on protein level were disappeared when using multilinear regression.

HMGA+NAT=without interaction model (Left column)

HMGA*NAT=with interaction model (right column).

Supplementary Table 11: Regression analysis of multiple variables in ZR-75 cells (virus infected)
HMGA+NAT=without interaction model (Left column)
HMGA*NAT=with interaction model (right column)

	MVM(-): HMGA + NAT				MVM(+): HMGA * NAT			
**Predictor**	**Beta**[1]	**95% CI**[2]	**p-value**	**VIF**[2]	**Beta**[1]	**95% CI**[2]	**p-value**	**VIF**[2]
HBS virus infected								
**nat_hbs**	4.7	-2.0, 11	0.094	6.5	12	-125, 148	0.5	224
**hmga_hbs**	0.10	-0.13, 0.32	0.2	6.5	0.42	-6.0, 6.9	0.6	429
R^2	0.987				0.991			
Adjusted R^2	0.973				0.962			
AIC	14.8				15.0			
**hmga_hbs * nat_hbs**					-0.08	-1.7, 1.5	0.6	1,199
AAT virus infected								
**hmga_aat**	0.09	-0.30, 0.49	0.4	37	-0.39	-1.1, 0.28	0.086	558
**nat_aat**	1.9	-21, 25	0.8	37	-81	-192, 31	0.069	4,605
R^2	0.971				1.00			
Adjusted R^2	0.942				0.999			
AIC	19.1				-1.43			
**hmga_aat * nat_aat**					0.18	-0.06, 0.43	0.067	8,132
shRNA virus infected								
**nat_shRNA**	6.9*	3.5, 10	**0.013**	1.8	2.9	-134, 140	0.8	198
**hmga_shRNA**	0.27*	0.07, 0.48	**0.030**	1.8	0.21	-2.0, 2.4	0.4	13
R^2	0.994				0.995			
Adjusted R^2	0.988				0.978			
AIC	14.1				15.5			
**hmga_shRNA * nat_shRNA**					0.06	-1.9, 2.0	0.8	272

[1] *p<0.05; **p<0.01; ***p<0.001

[2] CI = Confidence Interval, VIF = Variance Inflation Factor

Supplementary Table 12: Regression analysis of multiple variables in pancreatic epithelial and cancer cells (not infected)
HMGA+NAT=without interaction model (Left column)
HMGA*NAT=with interaction model (right column)

	MVM(-): HMGA + NAT				MVM(+): HMGA * NAT			
**Predictor**	**Beta**[1]	**95% CI**[2]	**p-value**	**VIF**[2]	**Beta**[1]	**95% CI**[2]	**p-value**	**VIF**[2]
Pancreatic Epithelial cells								
**nat_pan_epi**	37	-45, 119	0.2	2.6	113*	73, 154	**0.018**	24
**hmga_pan_epi**	-0.28	-3.9, 3.3	0.8	2.6	2.8*	1.1, 4.4	**0.030**	20
R^2	0.787				1.00			
Adjusted R^2	0.575				0.999			
AIC	17.6				-12.6			
**hmga_pan_epi * nat_pan_epi**					-32*	-48, -16	**0.025**	69
Miapaca-2 cancer cells(no virus infected)								
**nat_Miapaca**	4.5*	2.0, 6.9	**0.016**	1.0	36	-144, 217	0.2	1,898
**hmga_Miapaca**	0.00	-0.02, 0.01	0.6	1.0	0.18	-0.88, 1.2	0.3	1,440
R^2	0.970				0.995			
Adjusted R^2	0.940				0.980			
AIC	3.05				-3.87			
**hmga_Miapaca * nat_Miapaca**					-0.10	-0.67, 0.47	0.3	2,842
BxPC-3 cancer cells(no virus infected)								
**nat_Bxpc**	0.38*	0.09, 0.67	**0.030**	4.7	2.7	-7.6, 13	0.2	3,021
**hmga_Bxpc**	0.03*	0.01, 0.06	**0.033**	4.7	0.10	-0.19, 0.38	0.15	289
R^2	0.996				1.00			
Adjusted R^2	0.993				0.996			
AIC	-5.33				-14.3			
**hmga_Bxpc * nat_Bxpc**					-0.01	-0.03, 0.02	0.2	4,945
Panc-1 cancer cells(no virus infected)								
**nat_Panc**	0.43	-1.7, 2.5	0.5	25	1.5	-7.3, 10	0.3	102
**hmga_Panc**	0.02	-0.09, 0.13	0.6	25	0.06	-0.36, 0.49	0.3	85
R^2	0.966				0.991			
Adjusted R^2	0.931				0.966			
AIC	6.35				1.39			
**hmga_Panc * nat_Panc**					-0.01	-0.05, 0.03	0.3	271

[1] *p<0.05; **p<0.01; ***p<0.001

[2] CI = Confidence Interval, VIF = Variance Inflation Factor

Supplementary Table 13: Regression analysis of multiple variables in MiaPaCa-2 cells (virus infected)
HMGA+NAT=without interaction model (Left column)
HMGA*NAT=with interaction model (right column)

	MVM(-): HMGA + NAT				MVM(+): HMGA * NAT			
**Predictor**	**Beta**[1]	**95% CI**[2]	**p-value**	**VIF**[2]	**Beta**[1]	**95% CI**[2]	**p-value**	**VIF**[2]
HBS virus infected								
**hmga_hbs**	0.18**	0.13, 0.24	**0.005**	23	0.38	-5.6, 6.3	0.6	19,390
**nat_hbs**	0.64	-3.4, 4.7	0.6	23	2.4	-52, 57	0.7	285
R^2	1.00				1.00			
Adjusted R^2	0.999				0.999			
AIC	-0.893				0.312			
**hmga_hbs * nat_hbs**					-0.01	-0.38, 0.35	0.7	24,037
AAT virus infected								
**hmga_aat**	0.07	-0.15, 0.29	0.3	11	-0.06	-3.3, 3.2	0.9	177
**nat_aat**	-0.20	-4.0, 3.6	0.8	11	-5.5	-131, 120	0.7	872
R^2	0.886				0.911			
Adjusted R^2	0.772				0.646			
AIC	25.0				25.8			
**hmga_aat * nat_aat**					0.01	-0.15, 0.16	0.7	1,748
shRNA virus infected								
**hmga_shRNA**	0.09	-0.06, 0.24	0.12	20	0.06	-1.6, 1.7	0.7	148
**nat_shRNA**	0.30	-2.4, 3.0	0.7	20	0.01	-17, 17	>0.9	49
R^2	0.989				0.990			
Adjusted R^2	0.979				0.961			
AIC	17.2				18.9			
**hmga_shRNA * nat_shRNA**					0.00	-0.06, 0.06	0.8	279

[1] *$p<0.05$; **$p<0.01$; ***$p<0.001$

[2] CI = Confidence Interval, VIF = Variance Inflation Factor

Supplementary Table 14: Regression analysis of multiple variables in BxPC-3 cells (virus infected)

HMGA+NAT=without interaction model (Left column)

HMGA*NAT=with interaction model (right column)

	MVM(-): HMGA + NAT				MVM(+): HMGA * NAT			
**Predictor**	**Beta**[1]	**95% CI**[2]	**p-value**	**VIF**[2]	**Beta**[1]	**95% CI**[2]	**p-value**	**VIF**[2]
HBS virus infected								
**nat_hbs**	0.76**	0.45, 1.1	**0.009**	11	0.76	-5.1, 6.7	0.3	247
**hmga_hbs**	0.01*	0.01, 0.02	**0.017**	11	0.01	-0.23, 0.26	0.6	705
R^2	1.00				1.00			
Adjusted R^2	0.999				0.998			
AIC	-1.99				0.011			
**hmga_hbs * nat_hbs**					0.00	-0.01, 0.01	>0.9	1,702
AAT virus infected								
**hmga_aat**	0.11	-0.11, 0.34	0.2	9.1	-0.16	-2.2, 1.9	0.5	171
**nat_aat**	0.82	-13, 15	0.8	9.1	-5.2	-58, 48	0.4	30
R^2	0.962				0.990			
Adjusted R^2	0.925				0.962			
AIC	16.3				11.5			
**hmga_aat * nat_aat**					0.07	-0.45, 0.59	0.3	294
shRNA virus infected								
**hmga_shRNA**	0.10	-0.08, 0.28	0.14	16	0.83	-1.4, 3.1	0.13	2,443
**nat_shRNA**	0.19	-6.9, 7.2	>0.9	16	10	-21, 42	0.2	325
R^2	0.979				0.999			
Adjusted R^2	0.958				0.995			
AIC	14.3				1.80			
**hmga_shRNA * nat_shRNA**					-0.07	-0.28, 0.14	0.2	4,414

[1] *p<0.05; **p<0.01; ***p<0.001

[2] CI = Confidence Interval, VIF = Variance Inflation Factor

Supplementary Table 15: Regression analysis of multiple variables in PANC-1 cells (virus infected)
HMGA+NAT=without interaction model (Left column)
HMGA*NAT=with interaction model (right column)

	MVM(-): HMGA + NAT				MVM(+): HMGA * NAT			
**Predictor**	**Beta**[1]	**95% CI**[2]	**p-value**	**VIF**[2]	**Beta**[1]	**95% CI**[2]	**p-value**	**VIF**[2]
HBS virus infected								
**hmga_hbs**	0.16	-0.06, 0.39	0.086	7.3	0.56	-12, 14	0.7	1,636
**nat_hbs**	-0.56	-11, 9.9	0.8	7.3	10	-342, 362	0.8	546
R^2	0.970				0.974			
Adjusted R^2	0.940				0.896			
AIC	19.6				20.9			
**hmga_hbs * nat_hbs**					-0.09	-3.1, 2.9	0.8	3,910
AAT virus infected								
**nat_aat**	1.4	-1.7, 4.4	0.2	12	-29	-1,031, 974	0.8	86,445
**hmga_aat**	0.04	-0.09, 0.16	0.3	12	-0.58	-21, 20	0.8	21,164
R^2	0.984				0.986			
Adjusted R^2	0.969				0.946			
AIC	13.7				15.0			
**hmga_aat * nat_aat**					0.05	-1.5, 1.6	0.8	189,481
shRNA virus infected								
**hmga_shRNA**	0.12	0.00, 0.25	0.050	10	0.04	-1.8, 1.8	0.8	172
**nat_shRNA**	4.4	-0.01, 8.9	0.050	10	-13	-359, 334	0.7	5,016
R^2	0.997				0.998			
Adjusted R^2	0.995				0.992			
AIC	12.4				12.7			
**hmga_shRNA * nat_shRNA**					0.04	-0.71, 0.78	0.6	6,880

[1] *p<0.05; **p<0.01; ***p<0.001

[2] CI = Confidence Interval, VIF = Variance Inflation Factor

Supplementary Table 16: Regression analysis of multiple variables in AD293 cells (virus infected)

HMGA+NAT=without interaction model (Left column)

HMGA*NAT=with interaction model (right column)

	MVM(-): HMGA + NAT				MVM(+): HMGA * NAT			
**Predictor**	**Beta**[1]	**95% CI**[2]	**p-value**	**VIF**[2]	**Beta**[1]	**95% CI**[2]	**p-value**	**VIF**[2]
AD293 cells(no viral infection)								
**hmga_293**	0.10	-0.07, 0.26	0.13	52	0.09*	0.05, 0.13	**0.023**	53
**nat_293**	-0.03	-1.8, 1.7	>0.9	52	-1.2*	-2.2, -0.21	**0.041**	270
R^2	0.994				1.00			
Adjusted R^2	0.987				1.00			
AIC	6.19				-20.0			
**hmga_293 * nat_293**					0.00*	0.00, 0.01	**0.038**	242
AD293 cells(HBS virus infected)								
**nat_hbs**	19	-0.41, 38	0.052	34	24	-35, 83	0.12	62
**hmga_hbs**	-0.05	-0.31, 0.22	0.5	34	0.00	-0.73, 0.74	>0.9	52
R^2	0.995				0.999			
Adjusted R^2	0.990				0.994			
AIC	9.11				5.00			
**hmga_hbs * nat_hbs**					-0.06	-0.58, 0.46	0.4	89
AD293 cells(AAT virus infected)								
**hmga_aat**	0.01*	0.00, 0.02	**0.022**	1.8	-0.03	-0.65, 0.59	0.7	850
**nat_aat**	0.02	-0.37, 0.40	0.9	1.8	-0.97	-15, 13	0.5	257
R^2	0.977				0.987			
Adjusted R^2	0.953				0.948			
AIC	18.2				17.2			
**hmga_aat * nat_aat**					0.00	-0.02, 0.02	0.5	1,732
AD293 cells(shRNA virus infected)								
**hmga_shRNA**	0.10*	0.05, 0.14	**0.014**	5.2	0.10	-0.60, 0.80	0.3	63
**nat_shRNA**	0.12	-1.4, 1.6	0.8	5.2	0.34	-28, 29	>0.9	107
R^2	0.995				0.995			
Adjusted R^2	0.990				0.981			
AIC	11.2				13.1			
**hmga_shRNA * nat_shRNA**					0.00	-0.11, 0.10	>0.9	299

[1] *$p<0.05$; **$p<0.01$; ***$p<0.001$

[2] CI = Confidence Interval, VIF = Variance Inflation Factor

Supplementary Table 17: Regression analysis of HMGA1 and adenoviral CMV transcript interaction analysis.

S Table 17-A: ZR-75 cells

Table: Regression analysis of Adenoviral CMV-AAT in ZR-75

	MVM(-): HMGA + ad_cmv_AAT				MVM(+): HMGA * ad_cmv_AAT			
**Predictor**	**Beta**[1]	**95% CI**[2]	**p-value**	**VIF**[2]	**Beta**[1]	**95% CI**[2]	**p-value**	**VIF**[2]
**hmga_aat**	0.12	-0.87, 1.1	0.7	216	-0.34	-6.4, 5.7	0.6	1,004
**ad_cmv_AAT**	0.01	-0.59, 0.60	>0.9	216	0.07	-1.8, 1.9	0.7	258
R^2	0.969				0.986			
Adjusted R^2	0.939				0.944			
AIC	19.4				17.5			
**hmga_aat * ad_cmv_AAT**					0.00	-0.01, 0.01	0.5	469

[1] *p<0.05; **p<0.01; ***p<0.001

[2] CI = Confidence Interval, VIF = Variance Inflation Factor

S Table 17-B: MiaPaCa-2 cells

Table: Regression analysis of Adenoviral CMV-AAT in Miapaca-2

	MVM(-): HMGA + ad_cmv_AAT				MVM(+): HMGA * ad_cmv_AAT			
**Predictor**	**Beta**[1]	**95% CI**[2]	**p-value**	**VIF**[2]	**Beta**[1]	**95% CI**[2]	**p-value**	**VIF**[2]
**ad_cmv_AAT**	0.15*	0.04, 0.26	**0.026**	22	0.13	-1.6, 1.9	0.5	335
**hmga_aat**	-0.04	-0.11, 0.03	0.2	22	-0.05	-1.0, 0.90	0.6	221
R^2	0.994				0.994			
Adjusted R^2	0.988				0.976			
AIC	10.4				12.2			
**hmga_aat * ad_cmv_AAT**					0.00	0.00, 0.00	0.9	1,002

[1] *p<0.05; **p<0.01; ***p<0.001

[2] CI = Confidence Interval, VIF = Variance Inflation Factor

S Table 17-C: BxPC-3 cells

Table: Regression analysis of Adenoviral CMV-AAT in BxPC-3

	MVM(-): HMGA + ad_cmv_AAT				MVM(+): HMGA * ad_cmv_AAT			
**Predictor**	**Beta**[1]	**95% CI**[2]	**p-value**	**VIF**[2]	**Beta**[1]	**95% CI**[2]	**p-value**	**VIF**[2]
**hmga_aat**	0.21	-0.24, 0.67	0.2	49	0.01	-27, 27	>0.9	9,563
**ad_cmv_AAT**	-0.08	-0.51, 0.35	0.5	49	-0.08	-2.0, 1.9	0.7	57
R^2	0.971				0.971			
Adjusted R^2	0.942				0.886			
AIC	15.0				17.0			
**hmga_aat * ad_cmv_AAT**					0.00	-0.12, 0.12	>0.9	8,962

[1] *p<0.05; **p<0.01; ***p<0.001

[2] CI = Confidence Interval, VIF = Variance Inflation Factor

S Table 17-D: PANC-1 cells

Table: Regression analysis of Adenoviral CMV-AAT in Panc-1

	MVM(-): HMGA + ad_cmv_AAT				MVM(+): HMGA * ad_cmv_AAT			
**Predictor**	**Beta**[1]	**95% CI**[2]	**p-value**	**VIF**[2]	**Beta**[1]	**95% CI**[2]	**p-value**	**VIF**[2]
**ad_cmv_AAT**	0.08	-0.10, 0.26	0.2	20	-0.16	-1.7, 1.4	0.4	428
**hmga_aat**	0.02	-0.14, 0.18	0.6	20	-0.08	-0.83, 0.66	0.4	117
R^2	0.984				0.997			
Adjusted R^2	0.968				0.987			
AIC	13.8				7.84			
**hmga_aat * ad_cmv_AAT**					0.00	0.00, 0.00	0.3	895

[1] *p<0.05; **p<0.01; ***p<0.001

[2] CI = Confidence Interval, VIF = Variance Inflation Factor

S Table 17-E: AD293 cells

Table: Regression analysis of Adenoviral CMV-AAT in AD-293

	MVM(-): HMGA + ad_cmv_AAT				MVM(+): HMGA * ad_cmv_AAT			
**Predictor**	**Beta**[1]	**95% CI**[2]	**p-value**	**VIF**[2]	**Beta**[1]	**95% CI**[2]	**p-value**	**VIF**[2]
**hmga_aat**	0.01	-0.01, 0.04	0.2	14	0.03	-0.43, 0.49	0.6	324
**ad_cmv_AAT**	0.00	-0.02, 0.02	0.8	14	0.00	-0.09, 0.10	0.7	17
R^2	0.977				0.981			
Adjusted R^2	0.955				0.926			
AIC	18.0				19.0			
**hmga_aat * ad_cmv_AAT**					0.00	0.00, 0.00	0.7	381

[1] *p<0.05; **p<0.01; ***p<0.001

[2] CI = Confidence Interval, VIF = Variance Inflation Factor
