# Supplementary material for: Sequestration and suppressed synthesis of oncogenic HMGA1 using engineered adenoviruses decreases human pancreatic and breast cancer cell characteristics
Source: PLoS One. 2025 Nov 3;20(11):e0335934. doi: 10.1371/journal.pone.0335934 (PMC12582467; doi:10.1371/journal.pone.0335934)
Supplement: S1 Raw images — (PDF) [file pone.0335934.s002.pdf]

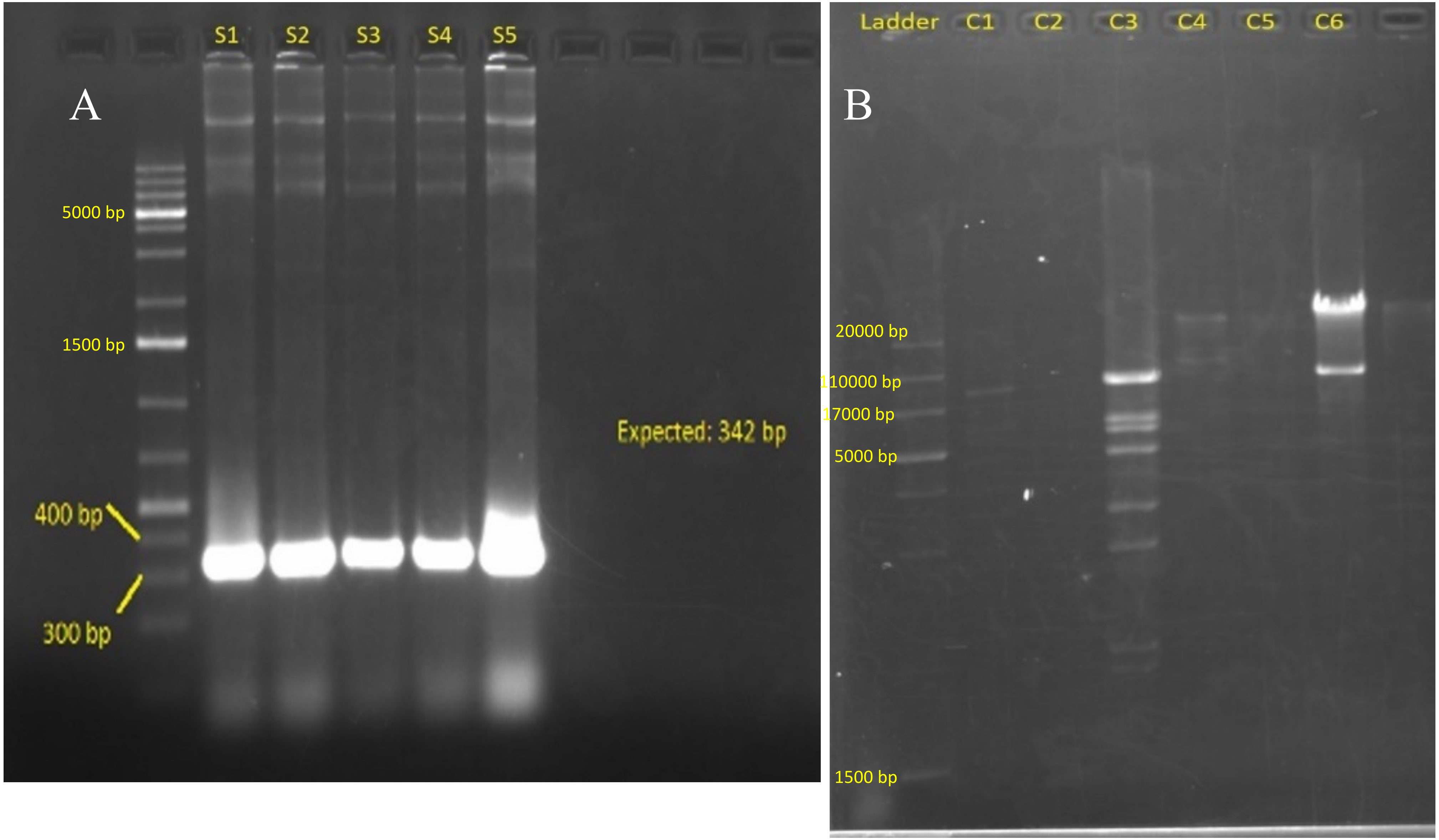

S Fig. 3: (A) PCR amplification of AAT insert sequence from pAd1127-07 plasmids extracted from E.coli. Expected band size of the product was 342 bp. Column S1 to S5 indicate different plasmid samples. (B) Characterization of cosmid plasmid digestion by HindIII restriction enzyme. C1 to C6 indicate different colony types. Only C3 shows corrected band pattern which was confirmed by the manufacturer, OD260 Inc., which shared that the HindIII digestion of the cosmid should give us the characteristic 9 fragments, 2 of them ~8-9 kb range (these 2 can overlap), 2 bands within ~6-7 kb range, one band ~5kb, 2 bands within 3-4 kb, 2 bands around ~2kb range. Only the 3rd lane (C3) match all band requirement, clearly other colonies did not have such a band pattern.

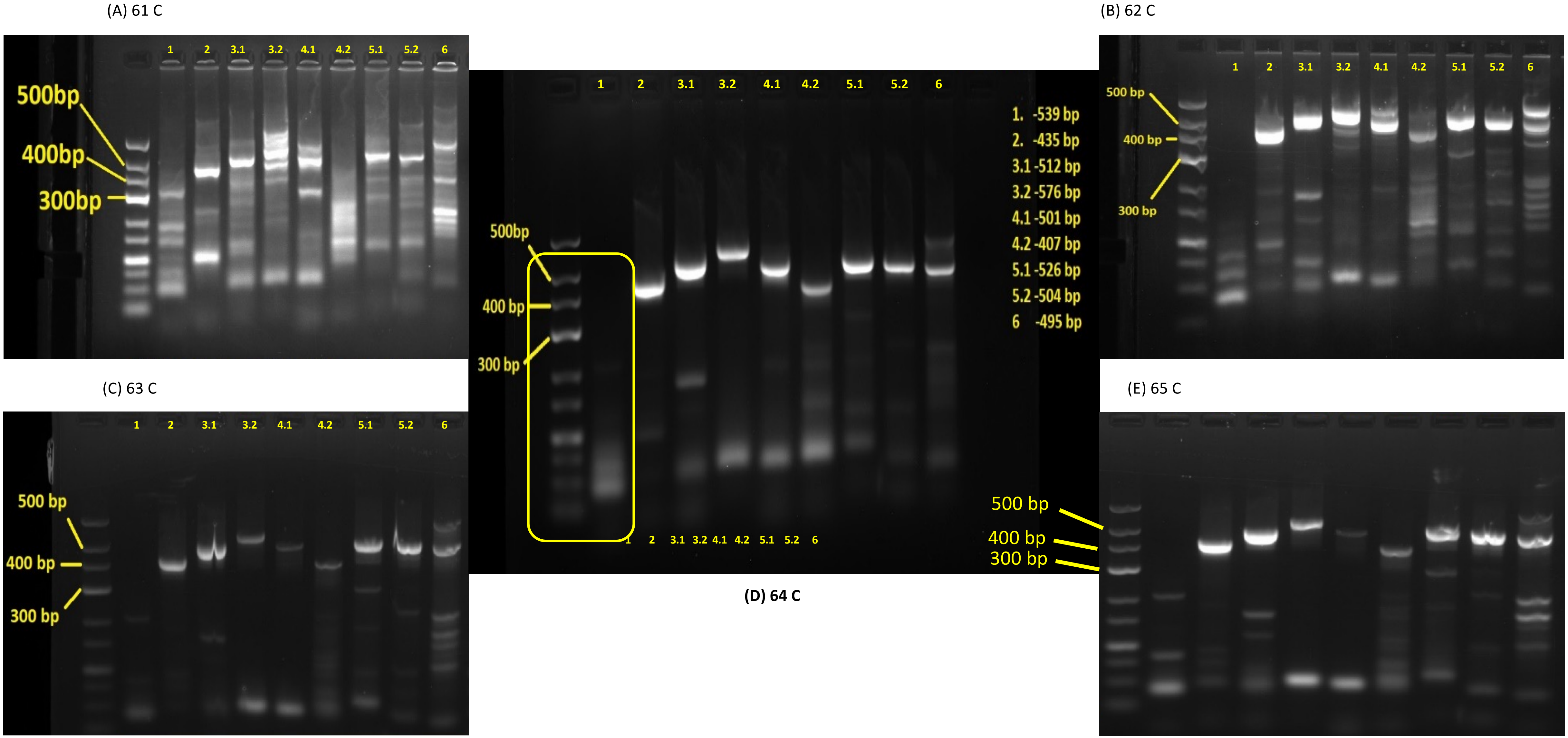

S Fig. 5: Optimization of NAT primers using gDNA as template. Annealing temperature was selected by performing PCR with gradual temperature increase. (A) Annealing temperature was 61C, (B) Annealing temperature was 62C, (C) Annealing temperature was 63C, (D) Annealing temperature was 64C, (E) Annealing temperature was 65C. Data showed that 64C temperature worked best than any other condition. Target band size (in bp) was shown in (D) inset.

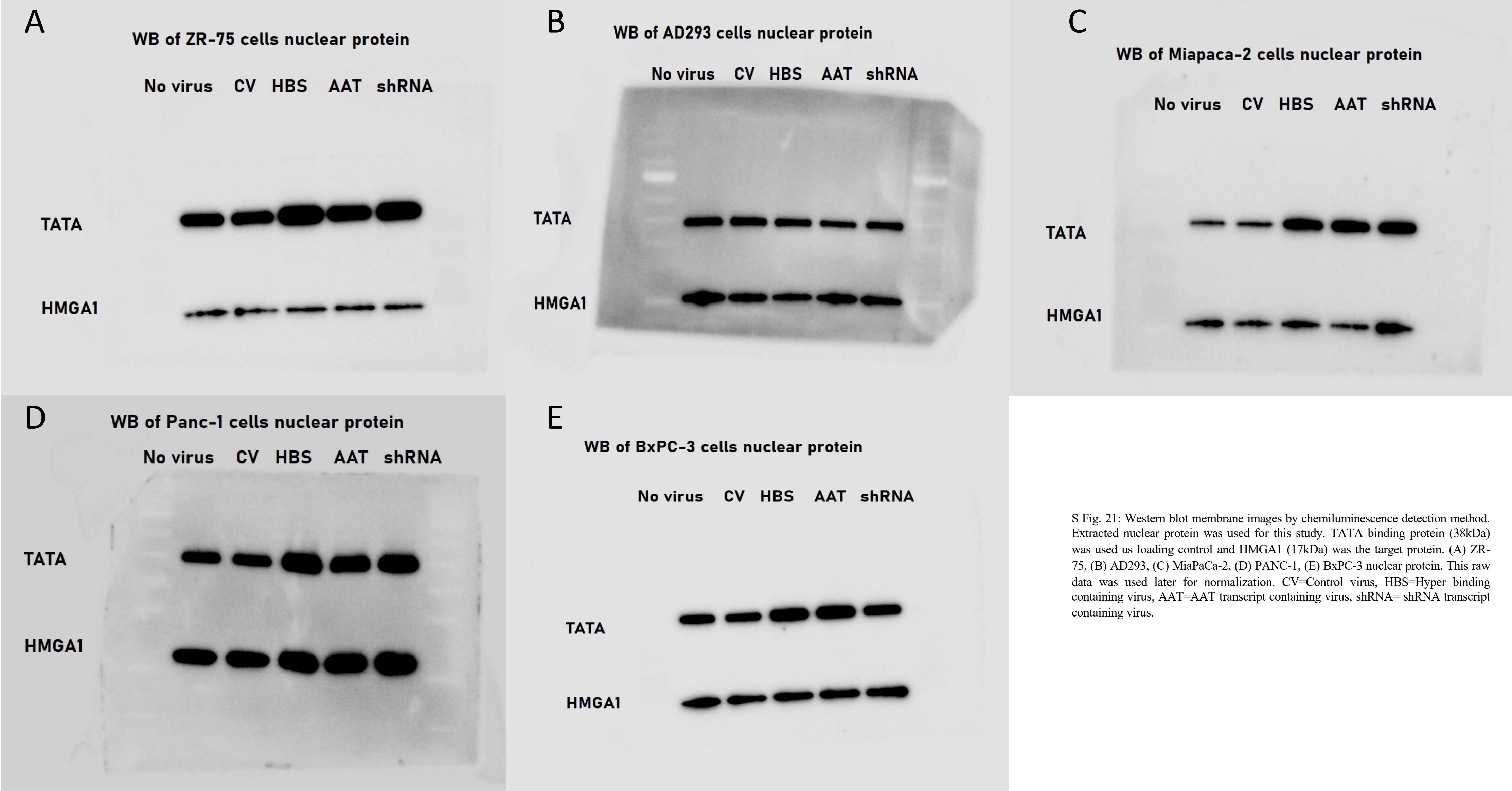

S Fig. 21: Western blot membrane images by chemiluminescence detection method. Extracted nuclear protein was used for this study. TATA binding protein (38kDa) was used us loading control and HMGA1 (17kDa) was the target protein. (A) ZR-75, (B) AD293, (C) MiaPaCa-2, (D) PANC-1, (E) BxPC-3 nuclear protein. This raw data was used later for normalization. CV=Control virus, HBS=Hyper binding containing virus, AAT=AAT transcript containing virus, shRNA= shRNA transcript containing virus.

A

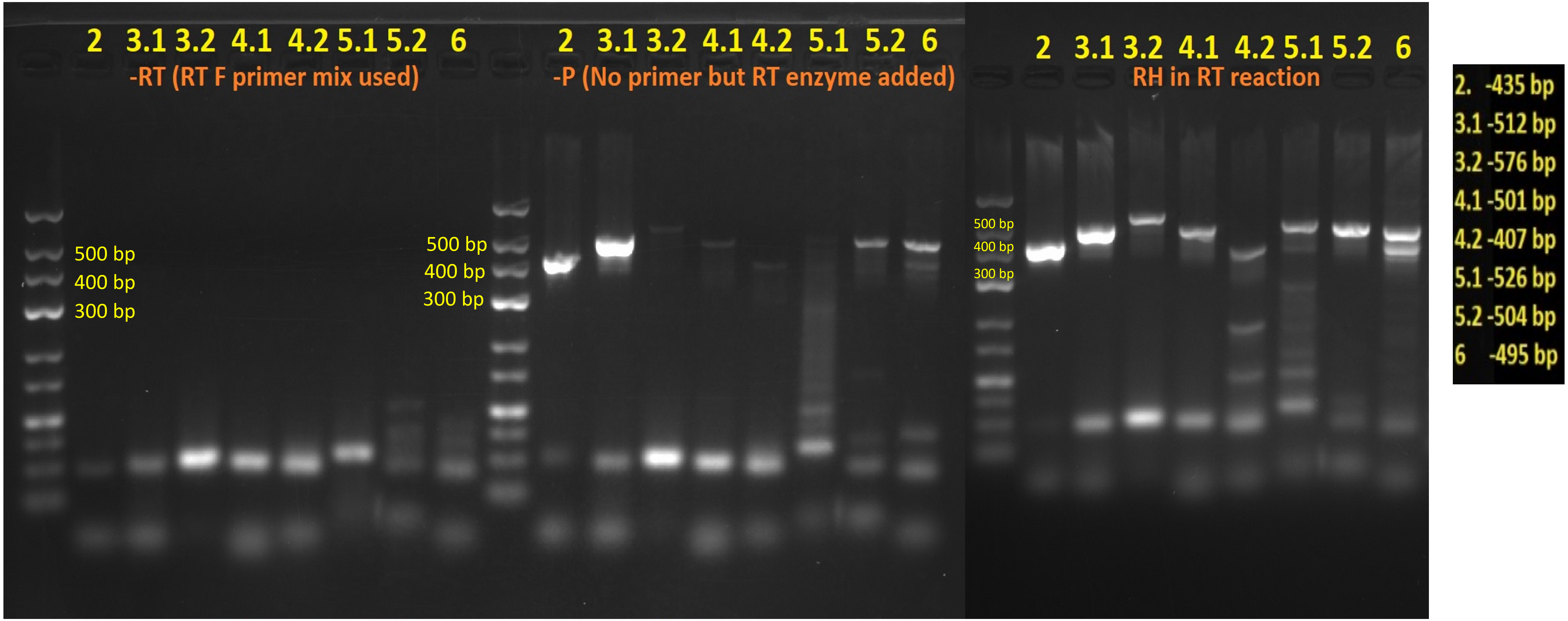

S Fig. 23: Detection of NATs from total RNA samples in different cancer cells and AD293. Total RNA was extracted from the cells followed by cDNA synthesis and PCR amplification with the NAT primers. (A) ZR-75, (B) MiaPaCa-2, (C) BxPC-3, (D) AD293. Column number indicated the position of the exon intron junctions. For all A,B,C,D, the left gel image indicated negative control (no RT enzyme), middle gel image indicated another negative control (no RT primer) and the right gel image indicated samples with all RT components. All target bands were separated by gel cut, purified and sequenced to confirm identity.

B

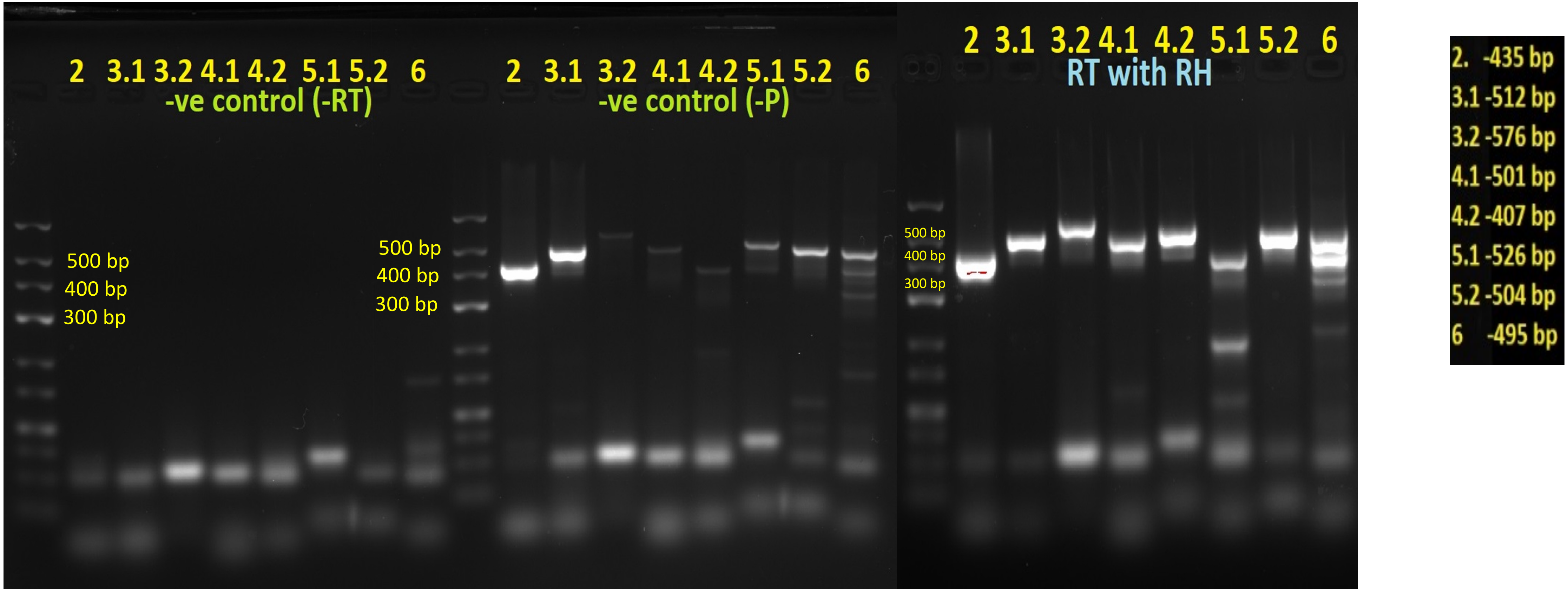

S Fig. 23: Detection of NATs from total RNA samples in different cancer cells and AD293. Total RNA was extracted from the cells followed by cDNA synthesis and PCR amplification with the NAT primers. (A) ZR-75, (B) MiaPaCa-2, (C) BxPC-3, (D) AD293. Column number indicated the position of the exon intron junctions. For all A,B,C,D, the left gel image indicated negative control (no RT enzyme), middle gel image indicated another negative control (no RT primer) and the right gel image indicated samples with all RT components. All target bands were separated by gel cut, purified and sequenced to confirm identity.

C

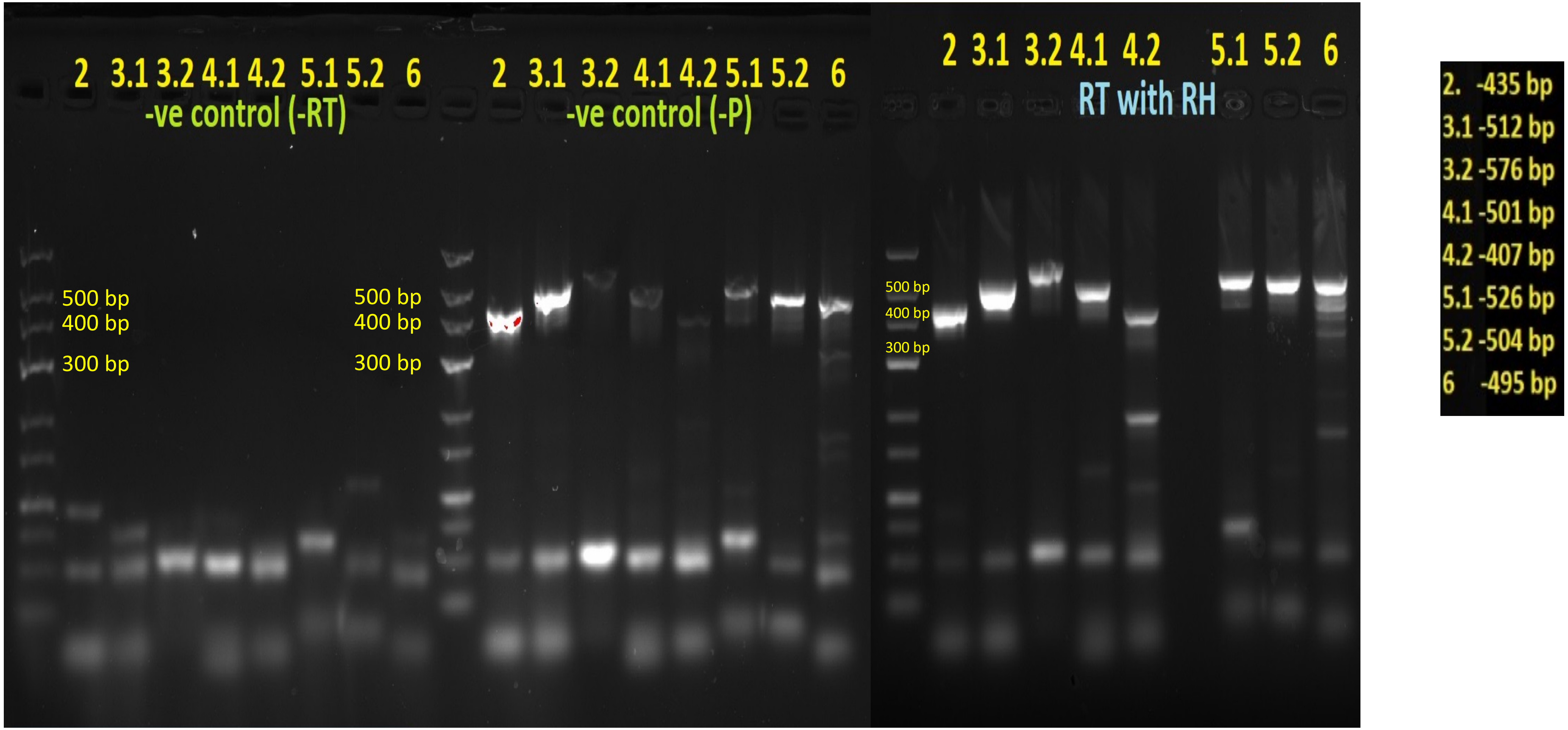

S Fig. 23: Detection of NATs from total RNA samples in different cancer cells and AD293. Total RNA was extracted from the cells followed by cDNA synthesis and PCR amplification with the NAT primers. (A) ZR-75, (B) MiaPaCa-2, (C) BxPC-3, (D) AD293. Column number indicated the position of the exon intron junctions. For all A,B,C,D, the left gel image indicated negative control (no RT enzyme), middle gel image indicated another negative control (no RT primer) and the right gel image indicated samples with all RT components. All target bands were separated by gel cut, purified and sequenced to confirm identity.

D

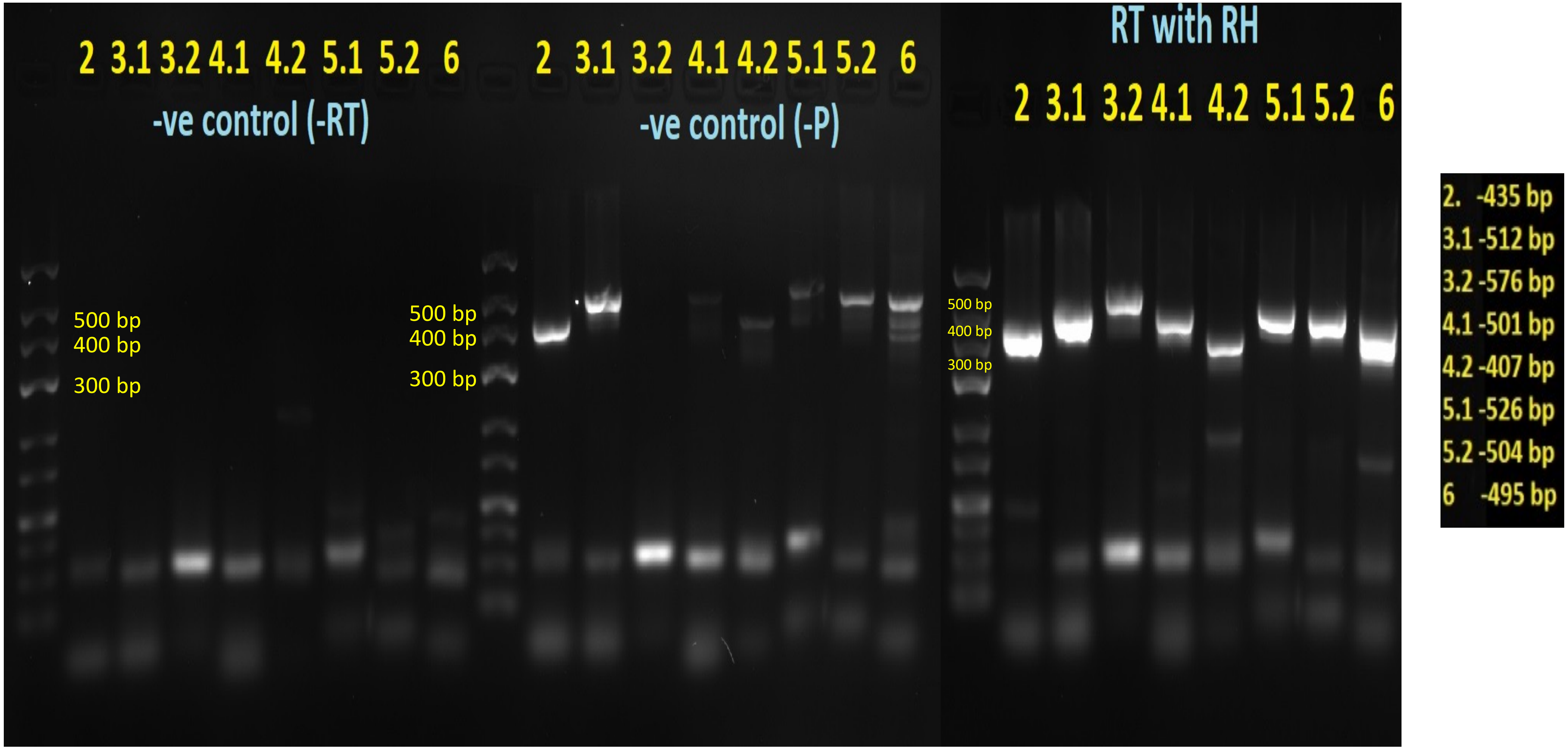

S Fig. 23: Detection of NATs from total RNA samples in different cancer cells and AD293. Total RNA was extracted from the cells followed by cDNA synthesis and PCR amplification with the NAT primers. (A) ZR-75, (B) MiaPaCa-2, (C) BxPC-3, (D) AD293. Column number indicated the position of the exon intron junctions. For all A,B,C,D, the left gel image indicated negative control (no RT enzyme), middle gel image indicated another negative control (no RT primer) and the right gel image indicated samples with all RT components. All target bands were separated by gel cut, purified and sequenced to confirm identity.
