# Supplementary material for: Sequestration and suppressed synthesis of oncogenic HMGA1 using engineered adenoviruses decreases human pancreatic and breast cancer cell characteristics
Source: PLoS One. 2025 Nov 3;20(11):e0335934. doi: 10.1371/journal.pone.0335934 (PMC12582467; doi:10.1371/journal.pone.0335934)
Supplement: Attachment — Submitted filename: response-letter-5-7-25.pdf [file pone.0335934.s004.pdf]

MIAMI UNIVERSITY
OXFORD, OH • EST. 1809

May 7, 2025

Editor, PLOS One

Dear Editor,

Please consider our revised manuscript entitled "Sequestration and suppressed synthesis of oncogenic HMGA1 using engineered adenoviruses decreases human pancreatic and breast cancer cell characteristics" for publication in PLOS One.

We have considered and addressed each of the reviewer's comments, as detailed below.

Responses to Reviewer's Comments

Comments Requiring Author Changes

1. Mechanistic Clarity:

- The study suggests that HMGA1 sequestration by the HBS virus initiates a negative feedback mechanism, but the exact molecular pathway remains unclear. Additional experiments (e.g., transcriptomic or proteomic analysis of downstream targets) could elucidate how HMGA1 sequestration leads to apoptosis.

Response – Agreed. Unfortunately, we are not in a position to conduct these additional experiments, which are outside our capabilities. However, to address this comment and to share this information with the readers, we have added the following text to the first paragraph in the "Overview of trends between HMGA1 protein levels, HMGA1 mRNA transcript levels and HMGA1 NAT levels" section:

"The molecular mechanism or pathway response for a negative feedback mechanism could be probed with additional experiments such as transcriptomic or proteomic analysis of downstream targets to elucidate how HMGA1 sequestration leads to apoptosis."

- For the AAT virus, the observed increase in HMGA1 mRNA levels without a corresponding rise in protein is intriguing. The authors propose mRNA stabilization via duplex formation, but direct evidence (e.g., RNA immunoprecipitation to detect AAT-mRNA hybrids) would strengthen this claim.

Response – Agreed. Again, however, these additional suggested experiments are beyond our capabilities. To address this comment, and to share this information with the reader, we have added the following sentence at the end of the paragraph in the "Overview of trends between HMGA1 protein levels, HMGA1 mRNA transcript levels and HMGA1 NAT levels" section:

"Direct evidence for such an interaction could be obtained experimentally, for example, by using RNA immunoprecipitation to detect AAT-mRNA hybrids."

2. In Vivo Validation:

- While the in vitro results are compelling, the therapeutic potential of these vectors would benefit from in vivo validation, such as xenograft mouse models, to assess efficacy, biodistribution, and toxicity in a physiological context. The authors previously demonstrated safety in mice (Hassan et al., 2018), but efficacy data in tumors are lacking.

Response – Acknowledged. However, the suggested xenograft mouse model study would represent an entirely new and distinct study that is beyond the scope of this manuscript. To capture this idea and share with the readers, we have added the following sentence to the last paragraph of the Conclusions section:

"Regarding potential translation to clinical use, the next steps would include in vivo validation, for example, the use of xenograft mouse model studies to assess the efficacy, biodistribution and toxicity in the context of a physiological study."

3. Comparative Analysis:

- The HBS virus outperforms AAT and shRNA viruses, but the reasons for this difference are not fully explored. Does HBS disrupt HMGA1's interaction with chromatin more effectively than AAT/shRNA block translation? A side-by-side comparison of chromatin accessibility or HMGA1-DNA binding (e.g., ChIP-seq) could provide mechanistic insights.

Response – This is a good suggestion. Again, we are not in a position to conduct these additional experiments. To convey this suggestion to the readers, we have added the following sentence to the fourth paragraph of the Conclusions section:

"A side-by-side comparison of chromatin accessibility or HMGA1-DNA binding using a ChIP-seq experiment could provide mechanistic insight into whether the HBS disrupts HMGA1's interaction with chromatin more effectively than the ATT/shRNA viruses blocked HMGA1 mRNA translation."

4. NAT Functional Role:

- The detection of a putative HMGA1 NAT is novel, but its functional role in regulating HMGA1 expression is speculative. Knockdown or overexpression of the NAT in cancer cells could clarify whether it stabilizes HMGA1 mRNA or modulates its translation.

Response – Agreed. However, the suggested knockdown or overexpression experiments would represent a substantial expansion of the study that we are unable to perform at this time. In order to convey the suggestion to the readers, we have added the following sentence to the end of the paragraph entitled "The Role of NATs in regulating HMGA1 transcript stability":

"Further HMGA1 knock-down or overexpression experiments could be conducted to clarify whether the HMGA1 NATs stabilize the HMGA1 mRNA or modulate its translation."

5. Statistical Reporting:
o Some figures (e.g., Figure 1E, 3F) summarize complex datasets but lack detailed statistical annotations. Clarify which comparisons are significant (e.g., HBS vs. AAT/shRNA) and adjust for multiple testing where applicable.

Response – The data presented in Figure 1E and Figure 3F are meant to illustrate trends in the data and facilitate comparisons across the multiple experiments, treatments and across the various cell lines tested. We are concerned that a comprehensive statistical comparison of all the data sets included in the figure would become unwieldy. Alternatively, we have removed the error bars from the plot, and instead, direct the readers to the supplementary material figures that contain all of the rigorous statistical analysis for each data set presented in the composite figures, as shown in the following revised figure captions for Figures 1E and 3F:

*“**Figure 1E)** A combined plot enabling comparison of viability, proliferation and wound healing assay for all engineered viral infections of ZR-75, MiaPaCa-2, BxPC-3 and PANC-1 cells. Detailed statistical calculations of each of these experiments were shown in supplementary figures (all viability assays in S Fig. 7-14, wound healing assay in S Fig. 15 and proliferation assay in S Fig. 16 A-C).”*

*“**Figure 3F)** A combined plot enabling comparison of the HMGA1 protein levels (right y-axis) and HMGA1 mRNA transcript and NAT transcript levels (left y axis) for all virus infected cell lines. Since the NAT copy numbers were much lower than mRNA copy numbers, the NAT values were multiplied by 10 (for 5.1 NAT position) or 100 (for 3.1 and 4.1 NAT positions) before plotting to facilitate comparison. Detailed statistical calculation of each experiment of this combined plot is shown in **S Fig. 22, 25-28**.”*

6. Introduction:

- To further strengthen the context of this work within current advances in gene therapy and viral vector engineering, the authors may wish to cite the following recent study in the Introduction or Discussion sections:
  Nature Biotechnology (2024). 10.1038/s41587-024-02453-3
  Including this reference would help readers appreciate the broader landscape of viral vector applications in oncology."

Response – To address this point, we have added the following sentence at the end of the 2nd paragraph in the Introduction section and added the suggested citation:

“It is worth noting that recent advances in viral vector engineering are providing powerful new tools for studying gene therapy approaches and its application in oncology research [9].”

- Briefly define "conditional replication" of adenoviruses for non-specialist readers.

Response – To address this point, we have added the following text in the first paragraph of the Conclusions section

“It should be noted that the background for the engineered viruses examined in this manuscript are adenovirus strains that were previously engineered to be “conditionally replicative” so that they can only replicate in cancer cells. This conditional replication activity originates from deletion of the 19-kDa and 55-kDa E1B genes [23-25]. The 55-kDa E1B protein inactivates the tumor suppressor protein p53, which is often mutated or disabled in cancer cells, but functional in normal healthy cells. Therefore, adenovirus strains lacking the 55-kDa E1B gene are

incapable of replicating in normal healthy cells but capable of replicating and lysing cancer cells that lack functional p53. Deletion of the 19-kDa E1B protein, which blocks the downstream effects of p53 to further prevent apoptosis, provides a secondary stringency for conditional replication in cancer cells only."

Minor Comments:

1. Figure Clarity:

- Figure 1B: Label wound healing images with time points (0h, 24h, 48h) for clarity.

Response – These labels have been added as suggested.

- Figure 3C-D: Include a schematic of the NAT detection strategy in the main text to aid interpretation.

Response – It wasn't clear how to add this schematic in the absence of using a figure, so we kept the schematic in the figure as Fig 3C, but we added the following text to clarify the explanation of the schematic and to clarify how the PCR-based NAT detection scheme was designed and used.

*"Unlike coding strand mRNA that has its introns spliced out, NATs may also contain the intron regions. The HMGA1 gene contains six introns and exons (**Fig 3C**). To probe for a potential cis-acting HMGA1 NAT, potentially including its introns, we designed a strand-specific RT-primer walk strategy with PCR primers spanning each intron-exon junction (**Fig 3C**). As the HMGA1 NAT mRNA would be oriented in the opposite direction as the HMGA1 coding strand, the strand-specific RT primers were designed downstream (on the putative NAT mRNA strand) of each intron-exon junction. The PCR primers used to detect the intron-exon junctions are listed in **S Table 1** and indicated in **Fig 3C**. In the absence of genomic DNA (gDNA), an experiment conducted with the PCR primers but in the absence of the RT enzyme would be expected to produce no PCR products. A primer walk experiment using PANC-1 cells is shown in **Fig 3D**. The negative control (NC) experiment without reverse transcriptase but including the intron-exon junction PCR primer pairs produced no bands indicating that the isolated total RNA was free from gDNA contamination. After this observation, random hexamer RT primers were used to maximize PCR products. A second negative control experiment was performed in which the RT primers were included in the reaction, but the PCR primers were omitted. In this case some PCR primer-independent DNA synthesis was observed for some fragments, apparently as a result of self-priming, which has been reported previously in the literature [15]. Finally, the RT-PCR experiment was performed with product-specific RT primers and with random hexamer RT primers, and strong RT-PCR products were detected at all intron-exon junctions indicating the presence a putative cis-acting HMGA1 NAT."*

2. Discussion Limitations:

- Address potential off-target effects of the adenoviral vectors (e.g., immune responses, unintended gene disruption) and how these might impact clinical translation.

Response – To address this point, we added the following sentence and new citation at the end of the last paragraph in the Conclusions section:

"Several limitations for translation of use of engineered adenovirus vectors to treat human cancers to clinical application have been discussed, including pre-existing immunity in humans, off-target effects such as inflammatory responses and off-target gene expression effects, sequestering to the liver and spleen and immunodominance of the vector genes over transgenes [27]."

3. Writing Edits:

- Abstract: Replace "~25% & 50" with "approximately 25% &50" for consistency.

Response – Replaced as suggested.

Additional Editor Comments:

1. The study is entirely in vitro (cell lines only). While the in vitro results are promising, no in vivo experiments (e.g., mouse tumor models) were performed to validate the efficacy or safety of the adenoviral treatments in an organismal context. This limits the translational relevance of the findings.

Response – Acknowledged. To address this point, the following text has been added to the final paragraph of the Conclusions section:

"While the in vitro results presented here are promising, no in vivo experiments have yet been performed to validate the efficacy or safety of the treatments in an organismal context, which limits the translational relevance of the findings at this point. Next steps for potential translation to clinical use would include in vivo validation, for example, the use of xenograft mouse model studies to assess the efficacy, biodistribution and toxicity in the context of a physiological study."

2. While decreased HMGA1 protein levels correlate with impaired cancer cell viability and migration, the precise molecular pathways downstream of HMGA1 sequestration or suppression are not investigated. The following related reference could be added to strengthen the manuscript: doi: 10.3389/fmed.2021.756988.

Response – Good suggestion. We have added the following sentence to the first paragraph of the Conclusions section to address this point:

"An example of how downstream pathway activation can be correlated to cancer cell behavior was reported in a recent of a related HMGB1 protein, where it was shown stimulation of exogenous HMGB1, which is ubiquitous in the tumor microenvironment and plays a vital role in tumor recurrence, was directly related to up-regulation of pathways promoting tumorigenesis and down-regulation of pathways suppressing tumorigenesis [23]."

3. In the introduction part, the authors should emphasize the dire need of diagnosis pancreatic cancer at an early stage and following references could be added: doi: 10.4251/wjgo.v16.i4.1256; doi: 10.2147/IJN.S335588.

Response –Agreed. To address this point, we have added the following text at the beginning of the 2nd paragraph of the introduction and added the two suggested references:

"Elevated HMGA1 levels play a critical role in pancreatic cancer tumorigenesis [6]. Pancreatic cancer remains a devastating disease with poor survival statistics and there remains a dire need for improved diagnostic techniques for early detection [7, 8]."

4. Although the viruses were said to have minimal cytotoxicity in healthy epithelial cells (H6c7, HMEC), more rigorous tests (such as assessing normal cell proliferation and gene expression post-treatment) would be needed to confirm safety.

Response –Acknowledged. To address this point, the following text has been added to 1st paragraph of the Conclusions section:

"The assays indicated that all three engineered viruses reduced cancer cell properties, including cell viability, cell proliferation and cell migration capability, all while causing minimal cell toxicity, which is an important factor when considering the safety for potential downstream clinical application. However, more rigorous testing of cell proliferation and gene expression effects post-treatment would be necessary to confirm safety."

5. The manuscript suggests that the HBS virus is "most effective" without fully acknowledging that all findings are in vitro and that translation to clinical

relevance is still speculative. Strong claims about therapeutic application are premature without animal model data.

Response – Acknowledged. To emphasize this point, we modified the last sentence of the abstract as follows:

"The HBS virus designed to sequester HMGA1 proved most effective in suppressing HMGA1 oncogenic activity in these in vitro cell-based studies compared to the AAT and shRNA viruses designed to suppress new synthesis of HMGA1 translation."

Further, we had already added the following text to the final paragraph of the Conclusions section, which also addresses this point:

"While the in vitro results presented here are promising, no in vivo experiments have yet been performed to validate the efficacy or safety of the treatments in an organismal context, which limits the translational relevance of the findings at this point. Next steps for potential translation to clinical use would include in vivo validation, for example, the use of xenograft mouse model studies to assess the efficacy, biodistribution and toxicity in the context of a physiological study."

6. Treatment with the AAT virus increased HMGA1 mRNA levels but still decreased HMGA1 protein levels.

Response – Acknowledged. This point had been raised by the reviewer, and we had addressed the point by including by adding the last sentence to this paragraph in the Overview of trends between HMGA1 protein levels, HMGA1 mRNA transcript levels and HMGA1 NAT levels section:

"Finally, a rather surprising result was observed following treatment with the AAT virus, in which the amount of HMGA1 mRNA levels increased by nearly three-fold in all of the cell lines, with a commensurate increase in the HMGA1 NAT levels, but the HMGA1 protein levels decreased to roughly the same levels as observed for treatment with the shRNA viruses. This result is consistent with the HMGA1 mRNA transcripts being protected from degradation when hybridized with the HMGA AATs, but not being available for translation, thus leading to reduced HMGA1 levels. Direct evidence for such an interaction could be obtained experimentally, for example, by using RNA immunoprecipitation to detect AAT-mRNA hybrids."

We hope that we have adequately addressed all of the reviewers and editors concerns at this point. We look forward to continued correspondence regarding our manuscript.

Sincerely,

Michael A. Kennedy, PhD

Professor
Department of Chemistry and Biochemistry
Miami University
Oxford, Ohio USA
