# Supplementary material for: Sequestration and suppressed synthesis of oncogenic HMGA1 using engineered adenoviruses decreases human pancreatic and breast cancer cell characteristics
Source: PLoS One. 2025 Nov 3;20(11):e0335934. doi: 10.1371/journal.pone.0335934 (PMC12582467; doi:10.1371/journal.pone.0335934)
Supplement: Attachment — Submitted filename: PLOS one HMGA1.doc [file pone.0335934.s005.doc]

The authors describe a novel adenoviral approach to target the oncogenic transcription factor HMGA1, which contributes to growth, migration, and chemoresistance in a variety of cancers. Three recombinant adenoviruses (HBS, AAT, and shRNA) targeting HMGA1 by sequestration, artificial antisense transcript, and shRNA expression, respectively, were evaluated in Pancreatic (MiaPaCa-2, PANC-1, BXPC3) and Breast (ZR-75) cancer derived cell lines. The data demonstrate that the HBS virus most potently downregulates HMGA1 and reduces cancer cell viability and migration with insignificant cytotoxic effects in normal cells. The existence of a putative HMGA1 NAT is a new and exciting finding in the HMGA1 regulation field. The manuscript is well-written, and the experiments are well performed and informative. However, some conceptual and contextual refinement would make it more convincing.

**Major points**

The evidence indicates that apoptosis is triggered by HMGA1 sequestration, but the molecular details of this induction are not known. Downstream targets (p53, BAX, BCL2, SMAD-dependent genes) may be responsible for this effect and may further strengthen the discussion.

The discovery of a cis-acting HMGA1 NAT is a significant result, but any functional consequence is speculation. The authors might discuss whether this NAT functions to stabilize HMGA1 mRNA, prevent miRNA binding or influence translation efficiency, like in other NAT–mRNA systems. A deeper mechanistic insight would be nice they just said in their paper that it will be done in the next paper.

The HBS virus, which was more effective than the AAT and shRNA viruses, sequestered rather than transcriptionally suppressed, yet the discussion does not adequately explain why sequestration is apparently more effective.

Although the study is well done in vitro, it would be nicer if there were a more ambitious statement about how these might be taken forward, perhaps for in vivo or translational work. Specifically, the inclusion of results from xenograft testing, immune response evaluation, and biodistribution studies would have signaled the attention to clinical development problems.

**Minor points**

Ensure that all figures include clear axis labels, time points, and statistical indications.

The manuscript is well written, but could be slightly condensed in the *Results* section by minimizing methodological repetition already described in *Methods*.
