# Supplementary material for: Sequestration and suppressed synthesis of oncogenic HMGA1 using engineered adenoviruses decreases human pancreatic and breast cancer cell characteristics
Source: PLoS One. 2025 Nov 3;20(11):e0335934. doi: 10.1371/journal.pone.0335934 (PMC12582467; doi:10.1371/journal.pone.0335934)
Supplement: Attachment — Submitted filename: response-letter-10-15-25.docx [file pone.0335934.s007.docx]

October 15, 2025

Editor, *PLOS One*

Dear Editor,

Please consider our revised manuscript entitled “**Sequestration and suppressed synthesis of oncogenic HMGA1 using engineered adenoviruses decreases human pancreatic and breast cancer cell characteristics”** for publication as a *Research Article* in the *PLOS One.*

Below, we address the remaining Reviewer’s comments.

Reviewer #1: This reviewer had no further comments or requests for changes.

Reviewer #3: This reviewer made the following comments:

Major points

1. The evidence indicates that apoptosis is triggered by HMGA1 sequestration, but the molecular details of this induction are not known. Downstream targets (p53, BAX, BCL2, SMAD-dependent genes) may be responsible for this effect and may further strengthen the discussion.

**Response: This was an excellent suggestion. We believe that the following revision to the manuscript significantly improves this section. We have added the following text and appropriate references at the end of the “Virus-mediated cell death…” section to discuss how virus-mediated decreases in HMGA1 levels could stimulate increased apoptosis:**

*“While there is evidence for triggering of apoptosis by all three engineered viruses, the molecular details for this induction are not known. All three viruses cause reduction in the cellular levels of HMGA1. Therefore, one can consider the potential downstream effects that lead to increased apoptosis upon reduction of HMGA1 levels. For example, HMGA1 is known to inhibit the p53 tumor suppressor protein [16, 17], thus blocking its ability to trigger apoptosis, therefore reductions in HMGA1 levels could stimulate increased apoptosis. Furthermore, HMGA1 has been shown to interact directly with p53 in a manner that blocks binding to the Bax promoter, which is a p53 and p21^waf1^ effectors, further blocking the tumor suppressor function of p53 [18]. It has also been reported that elevated levels of HMGA1 cause upregulation of Il-15 and Il-15Ra, which causes anti-apoptotic effects by inducing expression of apoptosis inhibitors Bcl2/Bcl1/Bcl-x(L) [19-21].”*

2. The discovery of a cis-acting HMGA1 NAT is a significant result, but any functional consequence is speculation. The authors might discuss whether this NAT functions to stabilize HMGA1 mRNA, prevent miRNA binding or influence translation efficiency, like in other NAT–mRNA systems. A deeper mechanistic insight would be nice they just said in their paper that it will be done in the next paper.

**Response: This point was partly addressed in the section entitled “The role of NATs in regulating HMGA1 transcript stability”. We have elaborated on our previous discussion by adding the following text and appropriate references:**

*“Therefore, cis-NATs may stabilize HMGA1 mRNA transcripts through cytoplasmic interaction of the NAT with the complementary single-stranded region of the mRNA sequence through interactions of the network of mRNAs, AS transcripts, micro-RNAs and RNA-binding proteins [27]. Another study has shown that mRNA stabilization by NATs can be mediated by cytoplasmic interaction of NATs with single-stranded regions of mRNAs that blocks microRNA destabilization of the mRNA [27, 28]. HMGA1 knock-down or overexpression experiments could be conducted to clarify the mechanism by which HMGA1 NATs stabilize the HMGA1 mRNA or modulate its translation.”*

3. The HBS virus, which was more effective than the AAT and shRNA viruses, sequestered rather than transcriptionally suppressed, yet the discussion does not adequately explain why sequestration is apparently more effective.

**Response: This result was surprising to us, but the observation was consistent across all experiments and across multiple cell lines. Determination of the underlying mechanism to understand this observation will require additional follow-up studies designed to probe the relative effectiveness of HMGA1-sequestration and reduced HMGA1 transcription. To address this point, we have added more text discussing potential mechanisms by which HMGA1 sequestration may so effective in the following paragraph in the Conclusions section, including addition of appropriate references:**

*“*	As infection with HBS virus exhibited the largest reduction of HMGA1 protein levels and caused significantly more cell death than the other viruses, we concluded that sequestration of HMGA1 protein had a higher impact on suppressing HMGA1 oncogenesis than blocking new synthesis of HMGA1. The precise mechanism by which HMGA1 sequestration reduced HMGA1 protein levels and caused increased cancer cell death remains unclear. The interpretation of the data presented here is complicated by many factors. For example, cells infected with a MOI of 50 ppc deliver 300 HMGA1 binding sites, a number that significantly outnumbers the copy numbers of the HMGA1 mRNA transcripts per cell for every cancer cell type studied. However, the number of protein molecules per mRNA transcript varies significantly by gene [37] and depends on many factors in addition to the number of mRNA transcripts per cell including mRNA stability, the speed of translation and the rate of protein degradation [38]. Another factor that must be considered is that most of the measurements were made at 72-96 hours after virus infection, which would permit at most a single doubling time (MiaPaCa-2 – 40 hrs, BxPC-3 48-60 hrs, Panc-1: 26-53 hrs and Zr-75: 80 hrs) under standard culture conditions. Therefore, any reduced expression due to shRNA or AAT virus infection would not block that action of the already expressed HMGA1, which would, however, be impacted by the sequestration strategy. Another factor that may have influenced the results is the relative accessibility of HMGA1 to the engineered virus HMGA1 binding sites relative to the accessibility of the genomic target HMGA1 binding sites that may be restricted in the context of the chromatin structure [39]. Further experiments may clarify these points, such as side-by-side comparison of chromatin accessibility or HMGA1-DNA binding using a ChIP-seq experiment could provide mechanistic insight into whether the HBS disrupted HMGA1’s interaction with chromatin more effectively than the ATT/shRNA viruses blocked HMGA1 mRNA translation. Based on the observation that HMGA1 sequestration resulted in reduced HMGA1 protein levels, we consider the possibility that an autoregulatory negative feedback mechanism may be involved in regulating HMGA1 levels in cancer cells. Increased cell death in cells with reduced HMGA1 levels could involve a chromatin remodeling mechanism, which HMGA1 is known to use in regulating gene expression, potentially activating apoptotic pathways that are ineffective in the presence of high HMGA1 levels. Further research is needed to determine the complex process by which HMGA1 sequestration reduces HMGA1 protein levels and causes cancer cell death.”

4. Although the study is well done in vitro, it would be nicer if there were a more ambitious statement about how these might be taken forward, perhaps for in vivo or translational work. Specifically, the inclusion of results from xenograft testing, immune response evaluation, and biodistribution studies would have signaled the attention to clinical development problems.

**Response: We believe that these points have already been addressed in the final paragraph of the Conclusions section, with the text as follows:**

*“While the in vitro results presented here are promising, in vivo experiments have not yet been performed to validate the efficacy or safety of the approach in an organismal context, which limits the translational relevance of the findings at this point. Next steps for potential translation to clinical use include in vivo validation, for example, the use of xenograft mouse model studies to assess the efficacy, biodistribution and toxicity in the context of a physiological study. Further obstacles that must be addressed prior to clinical use include pre-existing immunity in humans, characterization of off-target effects such as inflammatory responses and off-target gene expression effects, and sequestration to the liver and spleen and immunodominance of the vector genes over transgenes [40].”*

Minor points

5. Ensure that all figures include clear axis labels, time points, and statistical indications.

**Response: We believe that this has been addressed.**

6. The manuscript is well written, but could be slightly condensed in the Results section by minimizing methodological repetition already described in Methods.

**Response: Repetition was eliminated as requested, either by eliminating text in the Methods section or in the Results section.**

We hope that our revised manuscript is now considered suitable for publication. We look forward to correspondence concerning publication of our manuscript in the *PLOS One*.

Sincerely,

Michael A. Kennedy, PhD

Eminent Scholar and Professor

Department of Chemistry and Biochemistry

Miami University

Oxford, Ohio 45056
